# Noise in the Vertebrate Segmentation Clock Is Boosted by Time Delays but Tamed by Notch Signaling

**DOI:** 10.1016/j.celrep.2018.04.069

**Published:** 2018-05-15

**Authors:** Sevdenur Keskin, Gnanapackiam S. Devakanmalai, Soo Bin Kwon, Ha T. Vu, Qiyuan Hong, Yin Yeng Lee, Mohammad Soltani, Abhyudai Singh, Ahmet Ay, Ertuğrul M. Özbudak

**Affiliations:** 1 Department of Genetics, Albert Einstein College of Medicine, Bronx, NY 10461, USA; 2 Department of Computer Science, Colgate University, Hamilton, NY 13346, USA; 3 Department of Electrical and Computer Engineering, Biomedical Engineering and Mathematical Sciences, University of Delaware, Newark, DE 19716, USA; 4 Departments of Biology and Mathematics, Colgate University, Hamilton, NY 13346, USA; 5 Department of Pediatrics, University of Cincinnati College of Medicine, Cincinnati, OH 45229, USA; 6 Division of Developmental Biology, Cincinnati Children’s Hospital Medical Center, Cincinnati, OH 45229, USA; 7 Department of Pharmacology and Systems Physiology, University of Cincinnati College of Medicine, Cincinnati, OH 45229, USA

## Abstract

Taming cell-to-cell variability in gene expression is critical for precise pattern formation during embryonic development. To investigate the source and buffering mechanism of expression variability, we studied a biological clock, the vertebrate segmentation clock, controlling the precise spatiotemporal patterning of the vertebral column. By counting single transcripts of segmentation clock genes in zebrafish, we show that clock genes have low RNA amplitudes and expression variability is primarily driven by gene extrinsic sources, which is suppressed by Notch signaling. We further show that expression noise surprisingly increases from the posterior progenitor zone to the anterior segmentation and differentiation zone. Our computational model reproduces the spatial noise profile by incorporating spatially increasing time delays in gene expression. Our results, suggesting that expression variability is controlled by the balance of time delays and cell signaling in a vertebrate tissue, will shed light on the accuracy of natural clocks in multi-cellular systems and inspire engineering of robust synthetic oscillators.

## In Brief

Keskin et al. show that segmentation clock transcription levels display low amplitude and high heterogeneity. Clock gene expression variability is primarily driven by gene extrinsic sources, which is suppressed by Notch signaling. Gene expression noise increases along the posteroanterior axis. Spatial gradients of time delays contribute to the noise gradient along the axis.

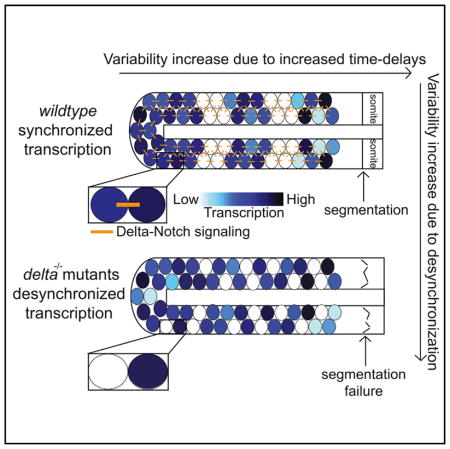

## INTRODUCTION

Gene expression is inevitably a highly stochastic process due to fluctuations in the complex stoichiometry and reaction kinetics of the biochemical reactions, and it leads to substantial cell-to-cell variability (Balázsi et al., 2011; Elowitz et al., 2002; Kaern et al., 2005; Ozbudak et al., 2002). The resulting phenotypic fluctuations can only be detected and quantified at the single-cell level within isogenic populations. One of the most intriguing questions in science is how developmental pattern formation is executed so robustly despite unavoidable fluctuations in gene expression. This precision necessitates several mechanisms buffering stochastic gene expression. Few studies to date have quantified stochastic gene expression in multi-cellular systems during development (Boettiger and Levine, 2013; Ji et al., 2013; Little et al., 2013; Raj et al., 2010), when buffering the process is critical for the precise and reproducible development of an adult organism, mainly because of technical difficulties posed by quantitative single-cell measurements (Boettiger and Levine, 2013; Ji et al., 2013; Little et al., 2013; Raj et al., 2010).

The study of somitogenesis provides us with an opportunity to investigate the regulation of spatiotemporal precision in pattern formation due to the tight coupling of space and time. The anterior-posterior axis of all vertebrates is patterned as a fixed number of repeating units, the vertebrae. The precursors of vertebrae are derived from somite segments that lay adjacent to the neural tube. Segmentation of somites is dictated by the period of a gene-expression oscillator, called the vertebrate segmentation clock, which is active in unsegmented cells. Oscillatory expression of the *Hes*/*her* genes is conserved in vertebrates, and disrupting their oscillatory expression results in vertebral segmentation defects (Pourquié, 2011).

The period of the zebrafish segmentation clock is short: 30 min. At the conclusion of each cycle, a cohort of ~200 cells buds from the unsegmented tissue to form a new somite. It remains unknown how segmentation clock factors accumulate to sufficient levels to orchestrate synchronized segmentation of groups of cells. Not only is this process robust and reproducible, but it also undergoes a set number of cycles in a given organism; in zebrafish, segmentation repeats 33 times to form the full-length body axis. Given these robust characteristics, the amplitude of oscillations should be tightly controlled. There is good understanding of the genetic circuitry of the segmentation clock (Pourquié, 2011). However, the field is lacking quantitative measurements of (1) the amplitude of clock gene oscillations, (2) the variability (noise) in clock gene expression between concurrently oscillating cells, (3) how this noise is suppressed in wild-type embryos but unrestrained in certain mutants, and (4) how the magnitude of variability differs spatially among phase-linked cells across a tissue.

To address these questions, we counted RNA molecules transcribed by two master segmentation clock genes (*her1* and *her7*) using single-molecule fluorescence *in situ* hybridization (smFISH) (STAR Methods) in intact zebrafish embryos. We found low amplitudes and high noise of *her1* and *her7* transcription in wild-type embryos. Cell-to-cell variability of clock gene expression is dominated by gene extrinsic noise. In Notch signaling mutants, amplitudes of oscillations decreased, while variability increased. Strikingly, transcriptional noise increased spatially from the posterior progenitor zone toward the anterior segmentation zone in both wild-type and Notch signaling mutant embryos. By computational modeling, we showed that the spatially increasing profile of gene expression noise can be recapitulated by the spatially increasing gene expression time delays in the clock network.

## RESULTS

### Segmentation Clock Oscillations Have Low Amplitudes

**Figure 1:**
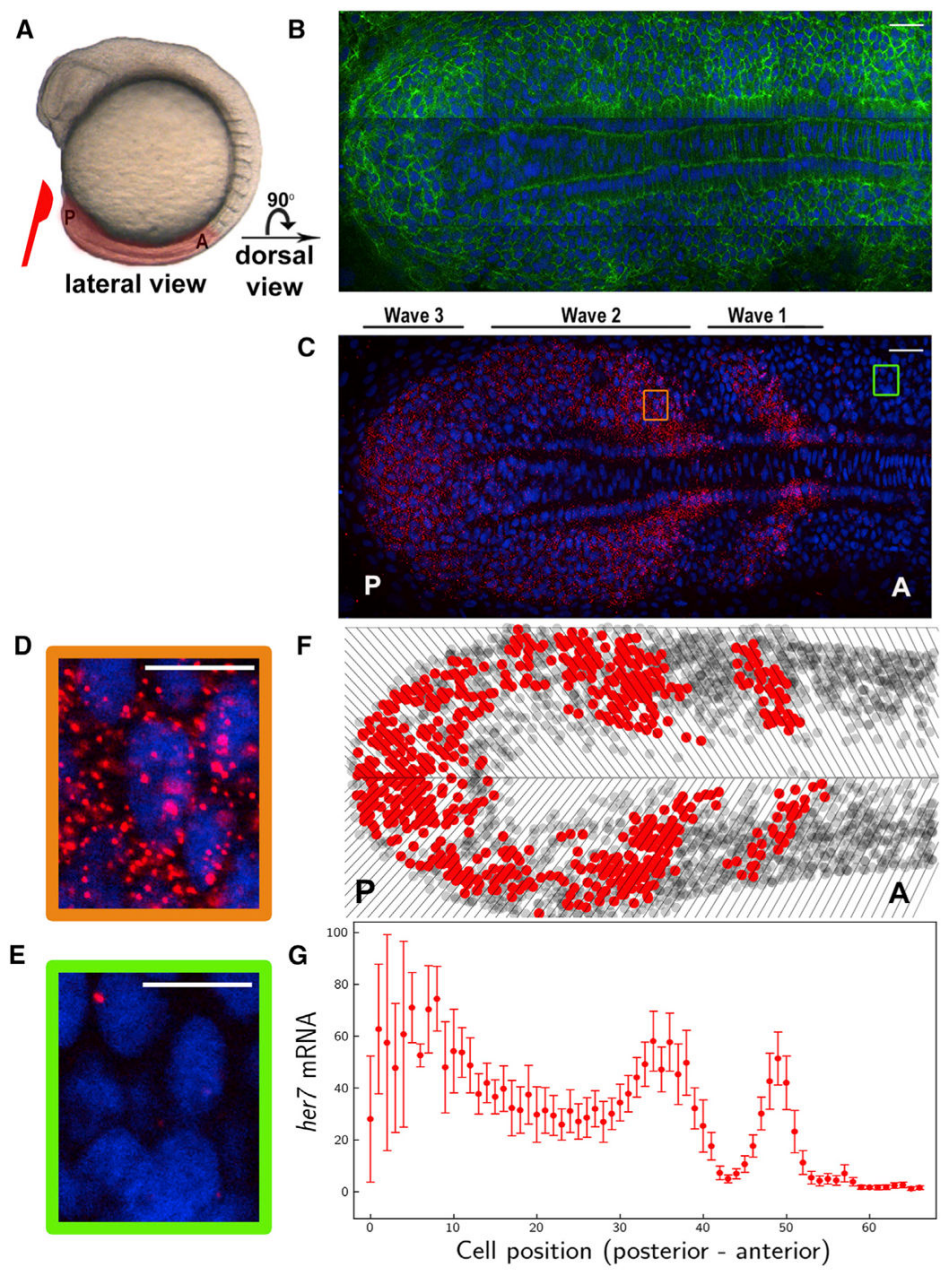
Single RNA Molecules Are Counted in Single-Phase-Binned Cells in the Intact Zebrafish Presomitic Mesoderm (A) A wild-type zebrafish embryo at the 10-somite stage. PSM, highlighted in pink, is dissected from the embryo. Posterior (P) is to the left, and anterior (A) is to the right. (B and C) A single z-section of a smFISH picture in a flat-mounted PSM from a wild-type embryo. Cell membrane (green in B), *her7* mRNA (red in C) and nuclear staining (blue in B and C). PSM images are rotated 90 degree compared to that in (A). (D and E) The images are zoomed on a high-expression stripe (orange square) (D) or a low-expression stripe (green square) (E). (F) Tissue is divided in single-cell-wide disks along the axis corresponding to different oscillation phases. Cells containing RNA higher or lower than an arbitrary threshold are plotted as red or gray circles, respectively. Top is left half of PSM, and bottom is right half of PSM. (G) RNA levels from left half of PSM are plotted along the posterior-to-anterior direction. Each dot corresponds to the average RNA number in a spatial phase-binned cell population; error bars indicate 2 SEs. Scale bars mark 30 and 10 μm in (B) and (C) and in (D) and (E), respectively. See also Figure S1 and Table S1.

We combined two strategies in our approach. First, we grouped cells based on their oscillation phases. The oscillation period of segmentation clock genes increases smoothly along the posterior-to-anterior (tail-to-head) direction of the presomitic mesoderm (PSM) but remains constant along the left-to-right and dorsal-to-ventral axes (Giudicelli et al., 2007; Gomez et al., 2008). The slowdown of oscillations along the posterior-anterior axis causes a phase delay in cells located in the anterior PSM compared to those located in the posterior PSM. As a result, one sees the different phases of the oscillator cycle mapped out in space along the length of the PSM. Hence, two to three kinematic waves of gene expression can be detected at any moment of an oscillation cycle (Figures 1A–1C). In other words, all cells located at the same posterior-anterior position in a 2-dimensional, single-cell-wide cross-section are in the same phase of oscillations (Mara et al., 2007; Ozbudak and Lewis, 2008; Riedel-Kruse et al., 2007). Exploiting this unique spatial-to-temporal correlation of the segmentation clock, we collected precise, quantifiable data describing the system in detail (Figure 1). Second, we quantified mRNA numbers in single cells in an intact tissue. We used smFISH to quantify the number of RNA molecules in phase-grouped cells along the posterior-anterior axis (Gross-Thebing et al., 2014; Wang et al., 2012). Single cells in the intact tissue were distinguished by jointly analyzing nuclear (DAPI) and cell membrane (membrane-localized GFP) markers (Figures 1B and S1). Serial sections of fluorescent images of flat-mounted embryos were captured by using a 63× (numerical aperture [NA] = 1.4) objective at 0.240 μm intervals for up to 30 μm (STAR Methods). Thereby, each RNA molecule was detected as a diffraction-limited bright fluorescent dot in the cell (Figures 1C–1G). The number of clock RNAs per cell did not systematically depend on the z axis, suggesting uniform RNA detection efficiency along the tissue depth (Figure S1F). To calculate background staining, we used segmented somites, in which expression of clock genes switches off, as a negative control. The background staining was very low (3 and 1 dots per cell for *her1* and *her7*, respectively). To assess the efficiency of RNA detection, we compared the total number of *her1* RNA molecules in the PSM of wild-type embryos detected by smFISH to that detected by qRT-PCR (ratio = 1.08 ± 0.37) (STAR Methods). These results validated that a single fluorescent dot in smFISH images corresponds to a single RNA molecule.

**Figure 2:**
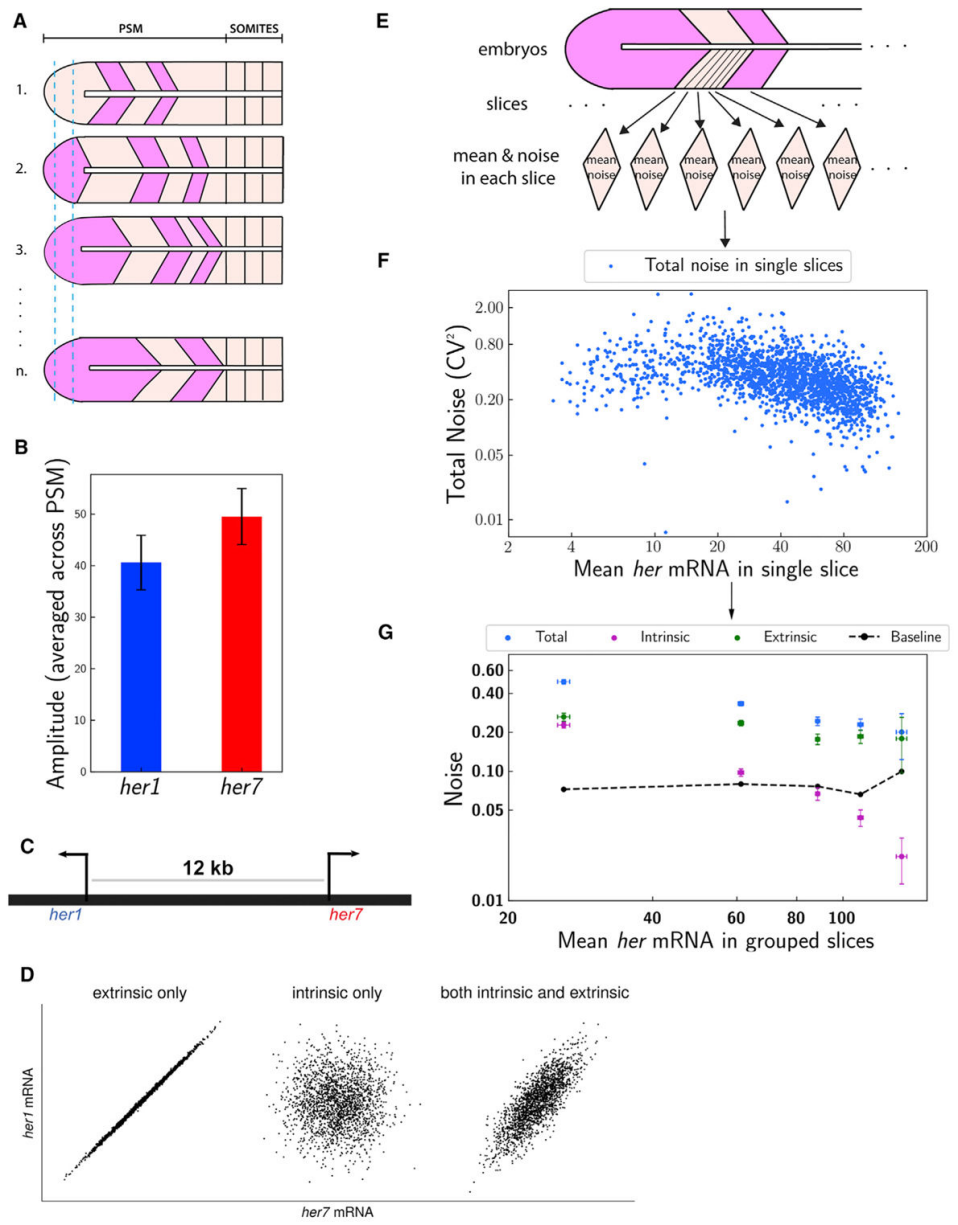
Extrinsic Noise Dominates Cell-to-Cell Expression Variability (A) Amplitudes of oscillations are measured comparing peak (pink) and through (cream) mRNA levels in cells corresponding to same position (such as marked between the vertical blue dashed lines) in the PSM of different embryos. (B) *her1* and *her7* have similar RNA amplitudes (averaged over all positions in the PSM). (C) Duplicated paralogous *her1* and *her7* genes are located in chromosome 5 adjacent to each other; distance between *her1* and *her7* gene loci is about 12 kb. (D) The expected profiles of her1 and *her7* RNA numbers if the variability is dominated by gene extrinsic sources (left panel), intrinsic sources (middle panel), or both. (E) The PSM is divided into single-cell-diameter spatial slices, grouping cells in the same clock phases. Mean expression and noise values are calculated for each slice in each embryo. (F) Total noise (CV^2^ = [SD/mean]^2^) versus mean levels of total *her* (*her1* + *her7*) RNA is plotted. Each blue dot represents the values in a single slice. All slice data from all wild-type embryos are pooled. (G) Total noise split into intrinsic and extrinsic components for each slice and all slice data reported in (F) grouped into five bins based on mean *her* RNA levels. The intrinsic noise decreases with the average RNA levels (magenta). Extrinsic noise (green) is the dominant component of total expression noise (blue), p < 0.001 (low and high expression). Total measurement error due to RNA counting, cell segmentation, and phase grouping of cells is plotted as a baseline (dashed black curve). y axis is noise; x axis is mean levels of total *her* (*her1* + *her7*) RNA in grouped slices. The graph is in log-log scale; error bars indicate 2 SEs. See also Figures S2 and S3.

We carried out a two-color smFISH experiment to count the number of *her1* and *her7* RNAs simultaneously in 10- to 14-somite-staged wild-type embryos. In total, we used 18 wild-type embryos. Each embryo captured a particular snapshot of the kinematic waves of the segmentation clock and hence displayed on-off stripes at different positions. The amplitude of oscillations was calculated by subtracting the RNA counts at each spatial position of embryos with low-expressing cells from that of embryos with high-expressing cells (Figure S2A) and taking the average across spatial positions (Figure 2A). Although prior computational modeling studies assumed larger amplitude oscillations in the segmentation clock (Ay et al., 2013, 2014), our data showed that *her1* and *her7* RNAs have average amplitudes of 41 ± 9 and 49 ± 9, respectively (Figure 2B). The number of clock transcripts is an order of magnitude lower than those of developmental genes controlling patterning and morphogenesis in *Drosophila* and housekeeping genes in mammalian cells (Boettiger and Levine, 2013; Little et al., 2013; Padovan-Merhar et al., 2015). It is remarkable that embryos can robustly accomplish segmentation by transcribing two master oscillating genes at such low levels, where they would be more vulnerable to gene expression noise (Balázsi et al., 2011; Elowitz et al., 2002; Ozbudak et al., 2002). Next, we investigated the magnitude of expression noise in the clock genes.

### Gene Extrinsic Noise Dominates the Total Cell-to-Cell Expression Variability

The two master segmentation clock genes (*her1* and *her7*) are adjacent but separated by a common regulatory sequence and transcribed in opposite directions (Figure 2C). These genes are paralogous duplicates and code for transcriptional repressors that function as hetero- or homo-dimers. Expression of clock genes is repressed by their protein products, which create a negative feedback loop that is thought to be the pacemaker of the segmentation clock (Harima et al., 2013; Lewis, 2003). *her1* and *her7* have similar transcriptional time delays (Hanisch et al., 2013) and RNA half-lives (Giudicelli et al., 2007). Transcription of *her1* and *her7* is therefore concomitant (Gajewski et al., 2003; Oates and Ho, 2002), and our preceding results showed that they have comparable amplitudes (Figure 2B). Intrinsic fluctuations are those due to the randomness inherent to transcription; being random, they should affect the transcription of *her1* and *her7* independently, producing uncorrelated variations in respective mRNA levels (Figure 2D) (Elowitz et al., 2002). Other molecular species in the cell, e.g., RNA polymerases and upstream transcriptional regulators, are gene products and therefore will also vary over time and from cell to cell. These variations cause correlated fluctuations in the expression of clock genes and are defined as extrinsic noise (Elowitz et al., 2002). To separate the contributions of intrinsic and extrinsic sources in the expression variability of these two clock genes, we took an approach similar to earlier works (Figure S3; Supplemental Experimental Procedures) (Elowitz et al., 2002; Rhee et al., 2014).

We first calculated expression variability among phase-grouped cells (single-cell-diameter spatial slices) (Figures 2E and 2F) and then further grouped the slices into five bins based on mean clock mRNA levels (Figure 2G). Our results showed that the total expression variability of segmentation clock genes, as described by CV^2^ ([SD/mean]^2^), is 0.49 (i.e., CV = 0.7) at the lowest transcription state and 0.20 (i.e., CV = 0.45) at the highest transcription state (Figure 2G), which is substantially higher than the measurement error (Figure 2G; Supplemental Experimental Procedures). The total clock gene expression noise (Figure 2G) is as high as the levels observed in single-cell systems (Golding et al., 2005; Raj et al., 2006; Taniguchi et al., 2010) but much higher than those of developmental genes in *Drosophila* (Boettiger and Levine, 2013; Little et al., 2013), likely due to lower transcript levels (Figure 2B). The gene extrinsic noise contributes most to total variability in transcription (p < 0.001), while the gene intrinsic noise is only high at low transcript levels and decreases proportionally to the total clock transcript level (Figure 2G). Slight differences in the promoter strengths of *her1*/*her7* genes and transcriptional time delays (Hanisch et al., 2013) and half-lives of *her1*/*her7* mRNAs (Giudicelli et al., 2007) might have slightly increased gene intrinsic noise in our calculations. However, our conclusion that extrinsic noise dominates clock transcriptional variability holds true. In yeast, noise in gene expression is primarily extrinsic in origin (Becskei et al., 2005; Colman-Lerner et al., 2005; Raser and O’Shea, 2004; Volfson et al., 2006); however, the intrinsic noise is found to dominate for *hb* patterning gene in *Drosophila* (Little et al., 2013). Cell cycle is less likely to contribute to extrinsic noise, because the segmentation clock period is much smaller than cell cycle and only a small population of cells experience mitosis during an oscillation cycle in the zebrafish PSM (Horikawa et al., 2006). We next assessed whether variation in cell size could underlie the dominant extrinsic noise. We reanalyzed our data by normalizing transcript counts by cell volume. The results showed that cell volume has only a minor contribution on the expression noise and extrinsic noise is the dominant portion of clock variability (Figure S2B). Because *her1* and *her7* genes are located adjacent to each other, common upstream factors (Becskei et al., 2005; Volfson et al., 2006) or local chromatin modifications are likely the main factors resulting in dominant extrinsic noise, as shown in yeast (Becskei et al., 2005), but one cannot exclude variations in epigenetic and metabolic states.

### Notch Signaling Buffers Mostly Gene Extrinsic Noise

After finding that extrinsic noise dominates the gene expression variability in the segmentation clock, we investigated the mechanism that might limit its magnitude. In *Drosophila*, noise in nascent transcription of *hb* gene is as high as that of the zebrafish segmentation clock genes, but noise in *Drosophila* patterning genes is averaged out by spatial averaging in syncytial embryo and by temporal averaging due to longer half-life of RNA (Little et al., 2013). However, spatial averaging is not possible in the vertebrate PSM cells, because they are not syncytial, and the short half-lives of RNAs (3–8 min) (Giudicelli et al., 2007) prevent simple temporal averaging.

**Figure 3:**
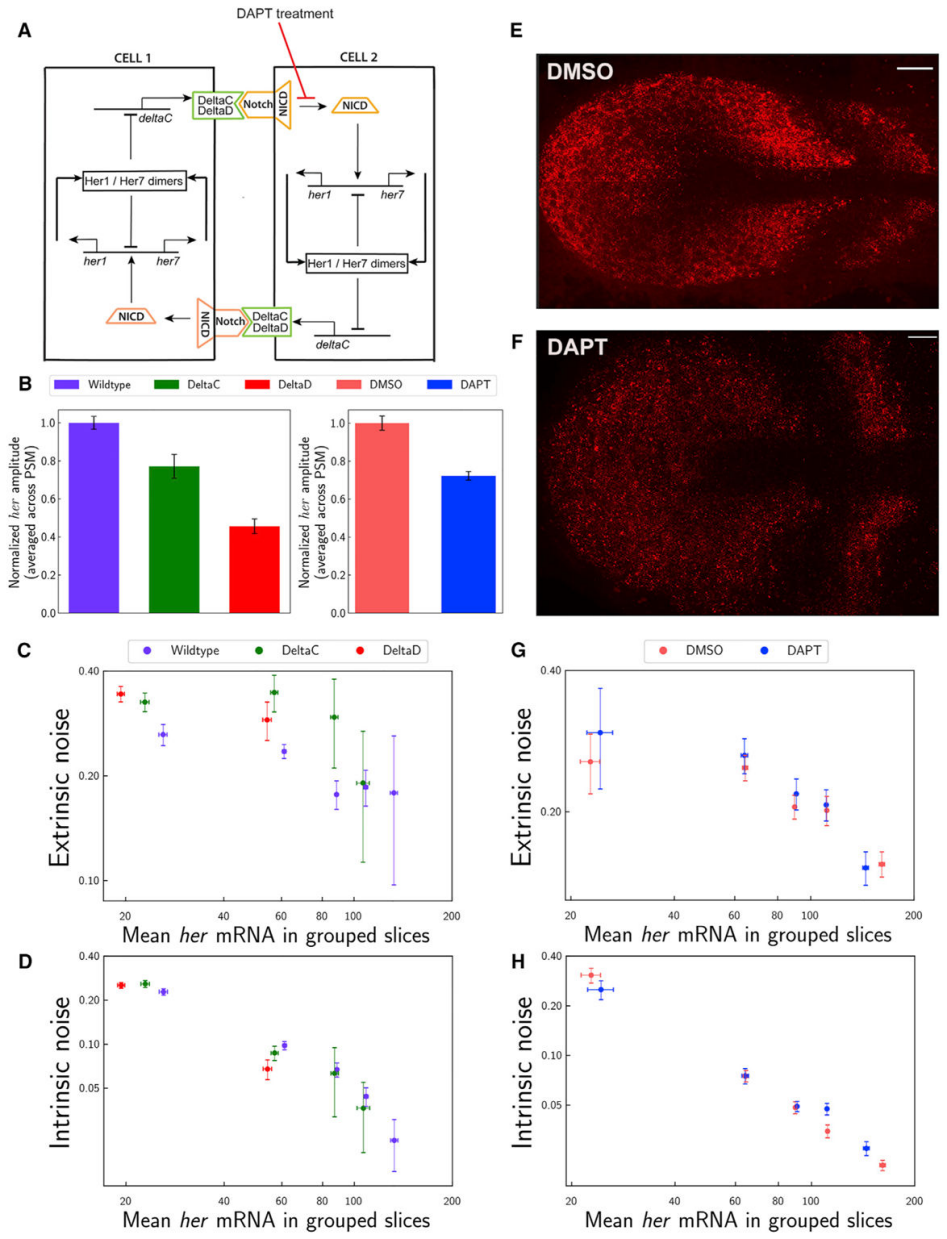
Notch Signaling Tames Extrinsic Noise (A) Cartoon model for Notch-mediated cell-to-cell coupling. Her dimers repress transcription of her-family genes and *deltaC*. DeltaC (and DeltaD) are membrane-bound ligands (green) that bind and activate Notch (orange) receptor in neighboring cells. Intracellular cleaved domain of Notch (NICD) activates transcriptions of her-family genes. (B) Total RNA amplitudes decreased in *deltaC*^−/−^ (green) and *deltaD*^−/−^ (red) mutants compared to wild-type embryos (purple) and in DAPT-treated embryos (blue) compared to DMSO-treated control embryos (pink) (p < 0.001). (C and D) Extrinsic noise (C) increased in *deltaC*^−/−^ (green) and *deltaD*^−/−^ (red) mutants compared to wild-type embryos (purple) (p < 0.001 in both lower and higher groups). Intrinsic noise levels (D) are shown in *deltaC*^−/−^ (green) and *deltaD*^−/−^ (red) mutants and wild-type embryos (purple). x axis is mean levels of total *her* (*her1* + *her7*) RNA in grouped slices. Clock transcription decreased in notch mutants. Therefore, slices could only be grouped for the lower expression bins compared to wild-type data. (E and F) A single z-section of a smFISH picture for *her7* mRNA (red) in DMSO-treated embryos (as a control, E) and DAPT-treated embryos (F). The stripy expression pattern of *her7* mRNA is not disrupted after 1.5 hr DAPT treatment (F). Scale bars mark 30 μm. (G and H) Extrinsic (G) and intrinsic (H) noise levels in DAPT-treated embryos (blue) and DMSO-treated control embryos (pink). Error bars indicate 2 SEs. See also Figure S3.

Oscillatory membrane-bound DeltaC ligands activate Notch receptors in neighboring cells; hence, Notch signaling acts at a short distance (Figure 3A). The activated intracellular domain of the Notch receptor, in turn, activates transcription of *her*-family genes. This short-distance coupling synchronizes oscillation phases among cells located in the same anterior-posterior position in the PSM (Delaune et al., 2012; Horikawa et al., 2006; Jiang et al., 2000; Mara et al., 2007; Ozbudak and Lewis, 2008; Riedel-Kruse et al., 2007). Based on experimental observations and computational modeling, Notch signaling has been hypothesized to minimize gene expression noise (Delaune et al., 2012; Horikawa et al., 2006; Jiang et al., 2000; Mara et al., 2007; Ozbudak and Lewis, 2008; Riedel-Kruse et al., 2007), but gene expression noise has not been quantified so far. To quantify noise, binary descriptions such as “synchronous versus asynchronous (salt-and-pepper)” are insufficient; one must count single molecules (Raj and van Oudenaarden, 2009) and quantify their distributions in cells that are grouped according to their oscillation phases. To investigate the impact of Notch signaling-mediated cell-to-cell coupling on expression noise, we carried out two-color smFISH experiments in two mutant lines—*deltaC*^−/−^ (Jülich et al., 2005) and *deltaD*^−/−^ (Holley et al., 2000)—in which Notch signaling is disrupted and stripy expression patterns are lost due to desynchronized oscillations. The amplitude of oscillations and mean RNA levels decreased (ranging from 23% to 54%) in two mutants compared to wild-type embryos (p < 0.006) (Figures 3B and S3). Because transcriptional noise depends on mean RNA levels (Figure 2G), we compared noise between mutants and wild-type embryos at similar mean RNA levels. The mutants lacked higher expression groups due to reduced clock transcription. Total expression noise increased (more than 30%) in two mutants compared to wild-type embryos due to extrinsic (p < 0.001), but not intrinsic, noise (Figures 3C and 3D). Extrinsic noise was elevated predominantly in the lower expression states. However, the mutant data reflect the steady-state response and might miss the initial outcome of loss of Notch signaling. To investigate this issue further, we transiently blocked Notch signaling by treating embryos with the γ-secretase inhibitor *N*-[N-(3,5-difluorophenacetyl)-L-alanyl]-S-phenylgly cine t-butyl ester (DAPT) or DMSO (as a control) for 1.5 hr. Earlier work showed that long-term DAPT treatment recapitulated segmentation defects in mutants (Mara et al., 2007; Ozbudak and Lewis, 2008; Riedel-Kruse et al., 2007). Two-color smFISH experiments showed that blocking of Notch signaling for only 1.5 hr did not disrupt the stripe expression patterns (Figures 3E and 3F) as reported previously, suggesting that the global phase synchrony is not disrupted with short-term DAPT treatment (Mara et al., 2007; Ozbudak and Lewis, 2008; Riedel-Kruse et al., 2007). However, the amplitude of oscillations decreased (28%, Figures 3B and S3) while the expression noise slightly increased in the DAPT-treated embryos compared to DMSO-treated embryos. As expected, the elevation of noise in DAPT- versus DMSO-treated embryos (Figures 3G and 3H) is much lower than that in mutants versus wild-type embryos (Figures 3C and 3D) due to the transient nature of drug treatment. Increased noise would then accumulate in time to cause both amplitude variability and phase drifting among neighboring cells and result in higher variability in mutant (*deltaC*^−/−^ and *deltaD*^−/−^) embryos. These results affirmed the long-standing hypothesis that the cause of segmentation defects in Notch signaling mutants is untamed gene expression noise in the segmentation clock genes (Jiang et al., 2000) and discovered that the main function of Notch signaling is to dampen extrinsic noise.

Although Notch signaling is an activator of clock transcription (Figure 3B), total noise did not primarily increase due to the decreased clock transcription in Notch mutants for the following reasons. First, gene intrinsic noise displays an inverse relationship with mean expression levels (Elowitz et al., 2002; Kaern et al., 2005) (Figure 2G). However, total noise increased in mutants due to increased extrinsic, but not intrinsic, noise (Figures 3C and 3D). Second, the amplitude of oscillations reduced twice in *deltaD*^−/−^ mutant compared to *deltaC*^−/−^ mutant, but intrinsic noise was not higher in *deltaD*^−/−^ mutant than in *deltaC*^−/−^ mutant. Altogether, these results show that total noise is elevated in Notch pathway mutants due to both desynchronized oscillations and decreased amplitudes, and surprisingly, these two factors increase only extrinsic, not intrinsic, noise. Extrinsic noise is the dominant component of the transcriptional variability in wild-type embryos (Figure 2G), which implies that transcriptions of *her1* and *her7* genes fire nearly simultaneously in each PSM cell. When Notch signaling is disrupted, extrinsic noise is elevated further (Figures 3C and 3D). This result shows that Notch signaling is not the source of co-transcription of two clock genes but rather actively tames gene extrinsic noise. We hypothesize that high extrinsic noise is likely due to the cell-to-cell variability in either the levels of other transcriptional regulators or the epigenetic state of the chromosomal locus.

### Gene Expression Noise Increases from the Progenitor to the Segmentation and Differentiation Zone

**Figure 4:**
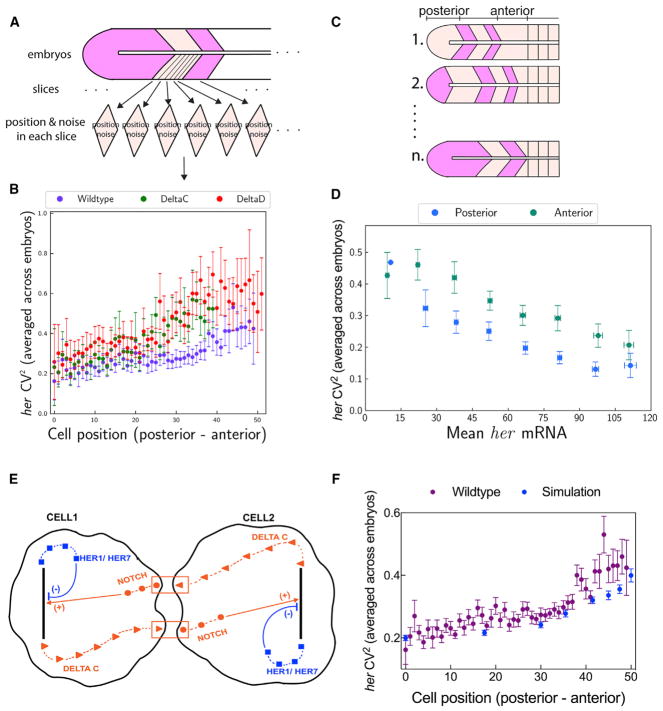
Expression Noise Displays an Increasing Profile along the PSM (A) Expression noise is analyzed in the PSM. (B) The total *her* expression noise increased in *deltaC*^−/−^ (green) and *deltaD*^−/−^ (red) mutants compared to that in wild-type (purple) throughout the PSM, p < 0.001 (wild-type and *deltaC*^−/−^ in posterior and anterior), p < 0.001 (wild-type and *deltaD*^−/−^ in posterior and anterior). x axis is spatial positions. (C) Representative sketches of n embryos at different oscillation snapshots are shown. Noise values from posterior and anterior PSM of all embryos are grouped in the first and second groups, respectively. (D) Expression noise is higher in cells located in the posterior versus the anterior PSM, p < 0.001. Expression noise (y axis) decreases as expression levels (x axis) increases, as shown in Figure 2G. However, at a given expression level, cells located in the anterior PSM have higher expression noise for the clock genes than cells located in the posterior PSM. The spatially increasing trend of expression noise (B) is independent of mean expression levels. (E) A cartoon figure shows intracellular and intercellular time delays. (F) The simulation result (blue) recapitulates increased total expression noise throughout the PSM (posterior to anterior) in wild-type embryos (purple). In (A)–(D) and (F), posterior is to the left and anterior is to the right. Error bars indicate 2 SEs. See also Figures S4 and S5.

Afterward, we assessed how noise in clock gene expression varies in space from the posterior PSM (progenitor zone) to the anterior PSM (where cells initiate segmentation) by pooling the noise values at each spatial position from all wild-type embryos (Figure 4A) (see STAR Methods for details). Strikingly, we found that expression noise increases toward the anterior PSM in wild-type embryos (p < 0.001) (Figure 4B). In Notch signaling mutants (*deltaC*^−/−^ and *deltaD*^−/−^), the spatial profile of gene expression noise does not flatten but rather elevated throughout the PSM (p < 0.001) (Figure 4B). Because total noise (CV^2^) depends on average expression levels (Figure 2G), we assessed the spatial change of noise by grouping data based on mean expression levels (see STAR Methods for details). Transcriptional noise is higher in cells located in anterior PSM than those in posterior PSM at comparable mean RNA levels (p < 0.001) (Figures 4C, 4D, and S4; STAR Methods). The results showed that the total noise is minimized by Notch signaling throughout the PSM in wild-type embryos but is boosted in Notch signaling mutants, and Notch signaling does not play a role in the anteriorly increasing trend of noise in wild-type embryos (Figure 4).

These results could be perceived as unexpected, because cell-to-cell variability should be minimized for cohorts of cells to segment robustly at the end of anterior PSM. This paradox can be explained by our earlier results, in which we have shown that the segmentation clock functions primarily in cells located in the posterior PSM. The segmentation clock relays its periodic information to downstream genes in the middle of the PSM (Giudicelli et al., 2007); thereafter, the clock is not needed for the segmentation process to be completed, although it continues to be expressed in the anterior PSM. It has been puzzling why expression of the segmentation clock became functionally irrelevant for cells located in the anterior PSM. Our results demonstrating increased gene expression variability of clock genes in anterior PSM reveal why cells could not rely on noisy clock expression in the anterior PSM. Collectively, our results show that there is selection pressure to minimize noise in clock gene expression only in the posterior PSM (Figure 4B). These results are the first demonstration of spatial variation of gene expression noise during vertebrate development.

We further investigated the mechanism driving spatial gradient of clock genes’ expression noise. A candidate mechanism is the spatially increasing effective gene expression time delays along the posterior-anterior direction (Ay et al., 2014). We built a simple stochastic computational model to test the impact of time-delay gradients on the expression noise in the tissue (Figures 4E and S5; Supplemental Experimental Procedures). In our model, we simulated synchronized oscillations in two neighboring cells. Cell-autonomous oscillations are generated due to an intracellular negative feedback loop (with an effective time delay of *τ_x_*), and the clocks are synchronized due to an intercellular positive feedback loop (with an effective time delay of *τ_y_*). We then repeated simulations by increasing the time delays gradually up to 4.5-fold—a value that matches to the experimentally quantified increase in effective time delays along the PSM (Ay et al., 2014). The simulations recapitulated the same spatially increasing trend in gene expression noise as time delays are increased along the tissue (Figure 4F).

## DISCUSSION

Many studies of stochastic gene expression have been performed with synthetic regulatory networks or promoter reporters for natural networks by using long-lived *GFP* mRNA and protein. However, many mRNAs coding for critical transcription factors governing embryonic development and adult homeostasis are short lived (Schwanhäusser et al., 2011). Here, by studying short-lived segmentation clock RNAs (t_1/2_ = 3–5 min) (Giudicelli et al., 2007), we investigated gene expression noise for the first time at the fastest dynamic scale (30-min period) in an intact vertebrate tissue.

Earlier work evaluated her/hes-family gene expression in embryos by simple binary scoring (signal present versus absent) (Delaune et al., 2012; Horikawa et al., 2006; Jenkins et al., 2015; Jiang et al., 2000; Mara et al., 2007; Ozbudak and Lewis, 2008; Riedel-Kruse et al., 2007), by arbitrary fluorescence units instead of absolute molecular counting (Webb et al., 2016), or in cell culture in which spatial information and Notch coupling were lost (Phillips et al., 2016; Webb et al., 2016). Here we used single-molecule counting in an intact vertebrate tissue to analyze the amplitude and variability of the segmentation clock genes in different genetic backgrounds and spatial positions. Our results showed that the amplitudes of transcriptional oscillations during somitogenesis are very low compared to RNA levels of developmental genes in other systems (Boettiger and Levine, 2013; Little et al., 2013) and extrinsic noise contributes the most to the high variability of clock gene expression (Figure 2). The period of oscillations is short (~30 min), and transcriptional time delays make up a significant portion of the period (Giudicelli et al., 2007). This would restrict the number of proteins that can be translated from a transcript in a given clock cycle. Unlike constitutively expressed and stable proteins, the segmentation clock proteins are short lived (Ay et al., 2013). Therefore, clock protein levels closely follow mRNA levels. Furthermore, clock RNAs and proteins establish a transcriptional negative feedback loop. All these features make clock mRNA levels the best reporters of clock protein levels. Here, we hypothesize that a major portion of transcriptional extrinsic noise is due to variability in clock protein levels. Although clock proteins have not been counted yet, published data revealed extensive cell-to-cell variability in Her1-Venus reporter levels (Delaune et al., 2012). In this study, we have investigated the precision in the amplitude of oscillations. Investigating the precision in the timing of oscillations (Morelli and Jülicher, 2007) would require carrying out single-molecule RNA or protein counting in real time. The levels of Her1/Her7 proteins should be above a threshold to repress many target genes, including *deltaC*; precision in their levels is therefore important for the successful segmentation of somites.

The timely and precise progression of a genetic program is critical to achieving reproducible embryonic development. Unlike other systems (Little et al., 2013; Shah and Tyagi, 2013), in somitogenesis, gene expression variability is not averaged in time or space. Here, we showed that vertebrate embryos use Notch signaling-mediated cell-to-cell coupling to minimize expression variability throughout the PSM (Figure 3). In the absence of Notch signaling, increased gene expression noise (Figure 3) desynchronizes clock oscillations and results in segmentation defects (Mara et al., 2007; Ozbudak and Lewis, 2008; Riedel-Kruse et al., 2007) and congenital scoliosis in patients (Pourquié, 2011). Lastly, we found that transcription noise increases spatially as cells approach the segmentation zone, likely due to spatially increasing gene expression time delays but independent of Notch signaling (Figure 4). Because time delays are inevitable in the expression of every gene, understanding the role of time delays in expression noise is significant. We showed that the spatial gradient of effective time delays in the PSM (Ay et al., 2014) likely contributes to the spatially increasing expression noise of the segmentation clock genes along the axis (Figure 4F).

Oscillations are widespread in biological systems. Notch signaling plays critical roles in controlling the switch from proliferation to differentiation in almost every tissue throughout the metazoan. *Hes/her* family genes are direct targets of Notch signaling. The segmentation clock was the first discovered developmental oscillator (Palmeirim et al., 1997). However, Hes/Her protein levels also oscillate in neural progenitors, embryonic stem cells, and ovarian cells, thereby controlling the switch from proliferation to differentiation in several tissues (Kobayashi and Kageyama, 2014). Furthermore, Hes/Her proteins are highly expressed in several types of human tumors, while their inhibition has been shown to restore differentiation (Kobayashi and Kageyama, 2014; Liu et al., 2015; Sang et al., 2010). We anticipate that future studies may lead to developing new ways to control stem cell proliferation and differentiation in various tissues, developing therapies against certain cancer types, understanding the precision of other natural oscillators, and engineering synthetic oscillators.

## STAR★METHODS

Detailed methods are provided in the online version of this paper and include the following:

### KEY RESOURCES TABLE

**Table:** 

REAGENT or RESOURCE	SOURCE	IDENTIFIER
Antibodies		
Rabbit anti-GFP primary antibody	Life Technologies	Cat#A6455; RRID:AB_221570
Goat anti-Rabbit IgG Alexa 488 Secondary Antibody	Thermo Fisher Scientific	Cat#A-11034; RRID:AB_2576217
Chemicals, Peptides, and Recombinant Proteins		
RNAscope Fluorescent Multiplex Detection Reagents	Advanced Cell Diagnostics	Cat#320851
Hoechst trihydrochloride, trihydrate	Invitrogen	Cat#33342
ProLong Gold antifade reagent	Life Technologies	Cat#P36934
SP6 mMessage mMachine	Life Technologies	Cat#AM1340
Research Quick RNA MiniPrep Kit	Zymo	Cat#R1054
SuperScript IV Reverse Transcriptase	Invitrogen	Cat#18090010
RNaseOUT	Invitrogen	Cat#10777-019
dNTP	Roche	Cat#05081955001
SYBR Select Master Mix	Life Technologies	Cat#4472908
N-[N-(3,5-Difluorophenacetyl-L-alanyl)]-S-phenylglycine t-Butyl Ester	Calbiochem	Cat#565770
RNAscope Probe-Dr-her1-LE1	Advanced Cell Diagnostics	Cat No. 433191
RNAscope Probe-Dr-her1-LE2-C3	Advanced Cell Diagnostics	Cat No. 433201-C3
RNAscope Probe-Dr-her7	Advanced Cell Diagnostics	Cat No. 428611
Experimental Models: Organisms/Strains		
Zebrafish: Tg(*Ola.Actb:Hsa.HRAS-EGFP*)	Cooper et al., 2005	ZFIN ID: ZDB-ALT-061107-2
Zebrafish: *dlc^tw212b/tw212b^*	Jülich et al., 2005	ZFIN ID: ZDB-FISH-150901-28480
Zebrafish: *dld^tr233/tr233^*	Holley et al., 2000	ZFIN ID: ZDB-ALT-980203-1047
Oligonucleotides		
Reverse Transcriptase primer: (5′-ACG TCT CGA GTC ACC AGG GTC TCC ACA AAG GCTG-3′)	This paper	N/A
q-PCR reactions forward primer: 5′-TGG AAG AAC TGC GAA CGC TT-3′	This paper	N/A
q-PCR reactions reverse primer: 5′-CGGAGGTTTTGG ATCATGCG-3′	This paper	N/A
Software and Algorithms		
Imaris 8.1.2	Bitplane	http://www.bitplane.com/imaris/imaris; RRID:SCR_007370
Python Programming Language, version 2.7.10	Python Software Foundation	http://www.python.org/; RRID:SCR_008394
Matlab_R2016a	Mathworks	http://www.mathworks.com/products/matlab/; RRID:SCR_001622
StochKit2	Sanft et al., 2011	https://sourceforge.net/projects/stochkit/files/
ImageJ		https://imagej.nih.gov/ij/; RRID:SCR_003070
GraphPad Prism 7	GraphPad	http://www.graphpad.com/; RRID:SCR_002798
Image Processing Pipeline	This paper	Data S1
Stochastic Simulations Script	This paper	Data S2

### CONTACT FOR REAGENT AND RESOURCE SHARING

Further information and requests for reagents may be directed to the Lead Contact Ertugrul Ozbudak (Ertugrul.Ozbudak@cchmc.org).

### EXPERIMENTAL MODEL AND SUBJECT DETAILS

#### Fish stocks

All the fish experiments were performed under the ethical guidelines of Albert Einstein College of Medicine (AECOM) and Cincinnati Children’s Hospital Medical Center, and animal protocols were reviewed and approved by the respective Institutional Animal Care and Use Committees (Protocol # 20150704 and Protocol # 2017-0048). Membrane-localized-GFP Tg(*b-actin:mgfp*)*^vu119^* (Cooper et al., 2005) transgenic line was used as wild-type, and *dlc^tw212b/tw212b^* (Jülich et al., 2005) and *dld^tr233/tr233^* (Holley et al., 2000) were used as Notch signaling mutants. Temporal loss of function of Notch signaling was accomplished by treating embryos with 100 μM of the γ-secretase inhibitor, *N*-[N-(3,5-difluorophenacetyl)-L-alanyl]-S-phenylgly cine t-butyl ester (DAPT) while DMSO is used as a control (Ozbudak and Lewis, 2008). Fish were bred and maintained at 28.5°C on a 14–10 hr light/dark cycle.

### METHOD DETAILS

#### smFISH and imaging

We have used the protocol developed by Advanced Cell Diagnostics (Wang et al., 2012), which allows for simultaneous detection of two differentially labeled transcripts in zebrafish (Gross-Thebing et al., 2014). 0.9 nL of membrane-localized GFP RNA was injected in one cell stage mutant embryos. Afterward, embryos were incubated at 23°C until they reach 10–14 somite stage, then embryos are fixed in 4% PFA in PBS for 1 hr at room temperature (RT), washed with 0.1% PBSTw (0.1% Tween-20 in PBS), then with gradually increasing methanol (MeOH) concentration (50% MeOH - 50 PBSTw, 100% MeOH), each wash for 5 min. Dechorionation was performed when they were in 50% MeOH - 50% PBSTw solution. Afterward, embryos were stored in 100% MeOH at −30°C overnight (O/N). The next day, embryos were air-dried for 30 min at RT and processed with Pretreat 3 (Advanced Cell Diagnostic RNAScope Pretreatment Reagents, 320842) for protease digestion for 20 min at RT. Then, they were washed with 0.01% PBSTw (0.01% Tween-20 in PBS) for 3 times each for 5 min. Probe-Dr-her7 and Dr-her1-LE2-C3 probes were mixed in 50:1 ratio, respectively, and probe mix was warmed at 40°C for 10 min in the oven, cooled down to RT, and added on embryos. Embryos were incubated in hybridization oven at 40°C for O/N. The next day all washing and signal amplification steps with RNAscope Fluorescent Multiplex Detection Reagents (Advanced Cell Diagnostics 320851) were performed according the RNAscope Protocol for Zebrafish (Gross-Thebing et al., 2014), with only few changes. Two additional washing steps were added both after the removal of probe and after the pre-amplifier hybridization step to decrease the background signal (5 times for 15 min) and each reagent was used as 1 drop. For labeling Amp4 Alt-B reagent was used. After the labeling step with Amp4, embryos were fixed in 4% PFA in PBS at 4°C for O/N. The following day, embryos were washed with 1% PBSTX (1% Triton X-100 in PBS) for 3 times each for 5 min, and permeabilized in 1.5% Triton X-100 for 1 hr at RT. Embryos were then incubated in blocking buffer (1% Triton X-100, 2%BSA, 5% Goat Serum) for 2 hr at RT. Then, embryos were incubated with Rabbit anti-GFP primary antibody (Life Technologies A6455) in blocking buffer (1:100) at 4°C for O/N. The following day, embryos were washed with 1% PBSTX 3 times for 10 min followed by a wash with blocking buffer for 10 min. Then, embryos were incubated in blocking buffer with 1:400 Hoechst trihydrochloride, trihydrate (Invitrogen 33342) and 1:200 Goat anti-Rabbit IgG Alexa 488 Secondary Antibody (A-11034, Life Technologies) at 4°C in dark for O/N. Then, embryos were washed with 0.2X SSCT (0.01% Tween-20 in 0.2X SSC) for 15 min at 4°C and fixed in 4% PFA in PBS. Embryos were mounted in 0.2X SSCT solution. ProLong Gold antifade reagent (Life Technologies P36934) was used to prepare slides. Images were captured by using a 63X (NA = 1.4) objective at the Zeiss Imager Z2. Serial sections of fluorescent images were taken at 0.240 μm intervals for up to 30 μm with AxioCamMRm camera, apotome and Axiovision software 4.8.2 Release. Images of single embryos were tiled along the x-y axis and stitched by the Axiovision software.

#### Quantifying RNA numbers in single cells

Imaris 8.1.2 Cell Module was used to analyze the images. First, the tissues surrounding the PSM (notochord, neural tube and lateral plate mesoderm were filtered by using the surface tool. Manual surface creation tool was selected to mark the regions covering undesired tissues and the voxels inside were set to zero. Individual PSM cells were identified by using the cell tool. Nuclei diameter threshold was set to 3 μm with smoothing width of 0.3 μm, background subtraction sphere diameter of 1.2 μm and split nuclei by seed points. Then, number of voxels filter was used to select the seed points and an intensity threshold was used for background subtraction, finally nucleus number of voxels was set to select single nucleus. Afterward, detect cell boundary from cell membrane option with 0.25 μm membrane width was selected. The cell volume filter was set between 150–450 μm^3^. Finally, spots tool was used to count the total number of RNA molecules in the PSM. Estimated diameter was set to 0.5 μm and background subtraction option was selected. Quality score filter was set to separate RNA dots from background. The cell-membrane signal (GFP) got weaker in the deepest z-layers, and thus we could not successfully separate cells and collect data on the ventral-most part (Figure S1). Therefore, we have only included data from cells that we could successfully segment by image analysis. The number of clock RNAs per successfully segmented cell do not systematically depend on z axis (Figure S1F).

#### qRT-PCR

Full-length *her1* mRNA standard was synthesized from her1 pCS2+ clone. The plasmid was linearized by Not1 and SP6 mMessage mMachine (Life Technologies AM1340) was used for *in vitro* transcription. RNA was purified with Zymo Research Quick RNA MiniPrep Kit (R1054). The loss of RNA during column purification was calculated by measuring the concentration of RNA by Qubit (Life Technologies) both before and after the column purification. Tails of five 13–14 somite-staged embryos were dissected and pooled in L15 media under a dissecting microscope and purified with Zymo Research Quick RNA MiniPrep Kit. Five biological replicates of this experiment were carried out. RT reactions were performed by using SuperScript IV Reverse Transcriptase (Invitrogen 18090010), RNaseOUT (Invitrogen 10777-019), dNTP (Roche 05 081 955 001) and the reverse primer: (5′-ACG TCT CGA GTC ACC AGG GTC TCC ACA AAG GCTG-3′). q-PCR reactions were performed by using the forward primer: 5′-TGG AAG AAC TGC GAA CGC TT-3′, reverse primer: 5′-CGGAGGTTTTGGATCATGCG-3′ and SYBR Select Master Mix (Life Technologies 4472908) with the Applied Biosystems StepOnePlus Real-Time PCR System. Standard RNA dilution series were prepared by 10-fold dilutions and the efficiency of primers was calculated by fitting a standard curve in the StepOne Software v2.3 (Slope: −3.32, R^2^: 0.98, Y-intercept: 33.423, Efficiency: 100%). The numbers of total *her1* mRNA in tail samples were calculated by comparing their C_T_ values to that of the standard *her1* RNA sample. We have detected 82264 ± 10816 *her1* mRNAs per single embryo.

#### Calculating the efficiency of RNA detection by smFISH

We counted the total RNA numbers in the whole PSM by smFISH and compared the results to that obtained by qRT-PCR. Spots tool was used to count the total number of *her1* RNA molecules in the PSM of 18 wild-type embryos. Estimated diameter was set to 0.5 μm and background subtraction option was selected. Quality score filter was set to above 70. We subtracted the nonspecific smFISH staining as follows: Surface tool was used to count the total number of nuclei (cells) in the PSM by using the DAPI channel data. Smooth tool was selected. Surface area detail level was set to 0.3 μm. Background subtraction with local contrast was used and the diameter of largest sphere which fits into the object was set to 1.2 μm. The threshold for background subtraction was set to 100. Split touching objects was enabled with seed point diameter as 3 μm. The number of voxels filter was used to separate the nuclei from smaller objects. The total number of cells was multiplied with the background smFISH staining and the resultant values were subtracted from that obtained with the spot detection tool to obtain the total number of *her1* mRNA molecules in a single embryo: 75862 ± 24061. The ratio of the number of *her1* mRNA molecules detected by qRT-PCR versus smFISH is: 1.08 ± 0.37.

#### Calculating the measurement error in RNA counting by smFISH

Control smFISH experiments were carried out in wild-type embryos by using two colors of probes (Dr-her1-LE1-C1 and Dr-her1-LE2-C3) directed against the *her1* mRNA. The probe sets are designed in an alternating tiled manner complementary to the *her1* mRNA. In total, we have used 8 wild-type embryos and imaged 21–35 sections in each embryo. The number of RNAs in each cell is measured as described in the above sections. Since binding of one probe set to RNA would be independent of the other probe set, the measurement error in counting single RNA molecules can be calculated by using an equation mimicking intrinsic noise: (1/2)*<*((*her*1*C*1/*<her*1*C*1 *>*) − (*her*1*C*3/*<her*1*C*3 *>*))^2^ *>*. The measurement error in RNA counting is: 0.03.

#### Calculating the measurement error in cell segmentation

One of the sources of measurement error would be incorrectly segmenting cells from each other, i.e., failing to assign image voxels unambiguously to a single cell. To estimate the cell segmentation error, we in total selected 38 image sections from 7 different wild-type embryos. We manually segmented 255 cells from this dataset by ImageJ. For each cell, we compared the manually segmented areas in ImageJ with the automatically segmented areas in Imaris. The measurement error of misassigning mRNAs to single cells is calculated as follows:

Let *x_i_* be an independent and identically distributed (iid) random variable denoting the actual mRNA count in a cell with mean 〈*x*〉 and coefficient of variation squared *CV*^2^. Assuming each cell has a normalized area of 1, the measurement is given by

(1) $$x_{i}(1-a_{i})+c_{i}x_{j},$$

where *a_i_* is an iid random variable denoting the fractional area ignored, *x_j_* is the mRNA count in the neighboring cell, and *c_i_* is an iid random variable denoting the fractional area gained of the neighboring cell. The mean and variance of *a_i_* is given by 〈*a*〉 and $\sigma_{a}^{2}$, respectively. Similarly, the mean and variance of *c_i_* is defined as 〈*c*〉 and $\sigma_{c}^{2}$. Then, the error in the measurement would be

(2) $$x_{i}(1-a_{i})+c_{i}x_{j}-x_{i}=c_{i}x_{j}-a_{i}x_{i}.$$

Taking the variance of (2) and dividing by 〈*x*〉^2^ quantifies the error in terms of the coefficient of variation squared:

(3) $$CV^{2}\left({\langle a\rangle }^{2}+{\langle c\rangle }^{2}\right)+\left({\left(\langle a\rangle -\langle c\rangle \right)}^{2}+\sigma_{a}^{2}+\sigma_{c}^{2}\right).$$

We calculated the segmentation error at different total *her* mRNA levels (Figure 2G). The measurement error values in cell segmentation ranged between 0.02 and 0.03.

#### Grouping cells in space based on oscillation phases

The position of each cell and the number of *her1* and *her7* RNA molecules in each cell were measured in each embryo. Each embryo was divided into left and right halves by visual inspection (Figure 1F). Within each half PSM tissue, cells were grouped (sliced) based on their oscillation phases, which vary smoothly along the axis (Giudicelli et al., 2007). The slice width was set to 8 μm, which corresponds to the diameter of cells in the PSM. The angles of slices were fixed to the angle of expression stripes of the segmentation clock in the PSM. The angle of expression stripes of the segmentation clock gene changes incrementally along the posterioanterior direction in the PSM. We first measured the stripe angles along the axis in all wild-type and DAPT-/DMSO-treated samples. We then fitted an equation to the data (Figure S1G). We obtained the following equations: wild type angle = 0.039 * Distance + 44.23, DMSO angle = 0.057 Distance + 43.45, and DAPT angle = 0.123 Distance + 21.43, where distance (μm) is measured from the tail end of embryos. Later on, we used these equations to incrementally change the angles of slices along the PSM in all embryos in each background. For *deltaC* and *deltaD* mutant embryos, we have used the angle function of the wild-type embryos. We assigned each cell into a slice when the center of the cell is located within a spatial slice.

#### Calculating the measurement error in phase grouping of cells

The calculation of expression noise depends on assignment of cells to correct oscillation phase bins. Part of the extrinsic noise could be due to slight errors in the assessment of cell positions rather than coregulation. To address this issue, the anterior stripe expression angles for wild-type embryos are calculated manually by two different experimentalists. We calculated two total expression noise values at different total *her* mRNA levels by using these two different angle sets. The measurement error is set to be the difference between total expression noise values obtained by using two independent measurements of expression angles. The measurement error in phase grouping of cells ranged between 0.01 and 0.05.

#### Calculating the total measurement error

We added the measurement errors due to RNA counting, cell segmentation and phase grouping of cells to obtain the total measurement error in our experimental and analysis pipeline at five total *her* mRNA levels (Figure 2G). The total measurement errors were between 0.066 and 0.098. We plotted the total measurement error in Figure 2G as a baseline to biological variability (expression noise) that can be unambiguously assayed.

### QUANTIFICATION AND STATISTICAL ANALYSIS

#### Heatmaps

For visual aid, we created heatmaps for each embryo by grouping the cells into two groups: cells with low or high mRNA numbers (Figure 1F). The threshold for this grouping was chosen as follows. First, the minimum (N) and maximum (M) mRNA levels per cell were determined in the embryo. Then the threshold was chosen as N+0.3*(M-N) by visual inspection. The cells below this threshold were assigned as low expression cells, and the rest were assigned as high expression cells. The heatmaps were created by plotting high expression cells with bold colors and low expression cells with light colors. Slice boundaries were shown in each heatmap (as in Figure 1F).

The details of the data analysis and computational modeling can be found in Supplemental Experimental Procedures.

## Supplementary Material

1

2

3

4

## Highlights

- Clock gene transcription levels display low amplitude and high heterogeneity
- Cell-to-cell coupling via Notch signaling tames primarily gene extrinsic noise
- Gene expression noise increases along the posteroanterior axis
- Spatial gradients of time delays contribute to the noise gradient along the axis
