# Supplementary material for: Noise in the Vertebrate Segmentation Clock Is Boosted by Time Delays but Tamed by Notch Signaling
Source: Cell Rep. Author manuscript; Available in PMC 2018 Jun 6. (PMC5989725; doi:10.1016/j.celrep.2018.04.069)
Supplement: 1 [file NIHMS970664-supplement-1.pdf]

Cell Reports, Volume *23*

Supplemental Information

Noise in the Vertebrate Segmentation Clock

Is Boosted by Time Delays

but Tamed by Notch Signaling

Sevdenur Keskin, Gnanapackiam S. Devakanmalai, Soo Bin Kwon, Ha T. Vu, Qiyuan Hong, Yin Yeng Lee, Mohammad Soltani, Abhyudai Singh, Ahmet Ay, and Ertuğrul M. Özbudak

SUPPLEMENTARY FILES

SUPPLEMENTAL METHODS

Subtracting nonspecific staining

In each genetic background, we quantified the nonspecific smFISH staining in the segmented somites, where *her1* and *her7* genes are not expressed. Mean and variance of nonspecific staining were given in Table S1.

Mean nonspecific staining levels were subtracted from each cell's mean *her1* and *her7* mRNA levels. Afterwards, cells with negative *her1* or *her7* mRNA levels were removed from the data. Slices with fewer than three cells after background subtraction were also removed from the data.

Spatial gene expression dynamics

Raw data was processed differently for this analysis. If a slice had negative mean of either *her1* or *her7* mRNA after background subtraction, the negative mean expression level was set to zero and the slice was not eliminated. For left and right halves of each embryo, *her1* and *her7* mean mRNA levels (y-axis) at each spatial location (slice) were plotted (x-axis). Spatial location 0 corresponded to the posterior (tail) end of embryos (Figure 1G).

Amplitude measurement

Slices with negative mean mRNA level were not eliminated but rather mRNA levels were set to zero. Amplitudes of *her1* and *her7* gene expression levels were calculated as follows:

Mean mRNA levels of each slice from all embryos were used to calculate amplitude for each genetic background. In this approach, we first combined the data from individual embryos in each genetic background as follows. First, we aligned the posterior ends of all embryos. Then, we combined the data from the *i*th slices from both left and right halves of all embryos. This iterative process was performed until one of the embryos ran out of slices. By this way, we collected 36, 28, 36, 18 and 24 data points for each cell position from 18 wild-type, 14 *deltaC*$^{-/-}$ mutant, 18 *deltaD*$^{-/-}$ mutant, 9 DAPT-treated, and 12 DMSO-treated embryos, respectively. To reduce the effect of outliers: a) We grouped data from every five consecutive spatial locations. Within each spatial group, mRNA levels of all slices were ranked from largest to smallest. b) Mean of the top 10% of the group was assigned as the peak and mean of the bottom 10% was assigned as the trough. The difference between the peak and trough in each group was defined as the amplitude at that location. We tested the effect of binning on amplitude values by varying the number of spatial groupings from 4 to 8 and top/bottom groupings from 5% to 10%. These modifications did not change the amplitude values more than 16%. The spatial profile of total *her* mRNA amplitude is plotted in Figure S2A. We then averaged the amplitude values across the space and plotted the average amplitudes in Figure 2B.

Noise formulas

The intrinsic and extrinsic noise levels for each slice were computed using the equations below. Total noise was calculated as the sum of intrinsic and extrinsic noise.

$$intrinsic\ noise = \frac{1}{2} < \left(\frac{her1}{< her1 >} - \frac{her7}{< her7 >}\right)^2 >$$

$$extrinsic\ noise = \frac{< her1\ \cdot her7 > - < her1 >< her7 >}{< her1 >< her7 >}$$

$$\mathrm{CV}^2 = total\ noise = intrinsic\ noise + extrinsic\ noise$$

For each genetic background, the three noise levels were plotted at different mean *her* (*her1*+*her7*) mRNA levels. We have reported how gene expression noise varies with respect to mean *her* expression levels among phase grouped cells (single-cell diameter spatial slices) in wild-type embryos in Figure 2F. Later on, we have grouped the noise data of individual slices (Figure 2F) into 5 bins according to their mean *her* RNA numbers (Figure 2G).

The contribution of differences in cell volumes on expression noise

We calculated the concentration of mRNAs in each cell by dividing the number of mRNAs in each cell by the cell volume. Later, we normalized the resulting values to report the number of mRNAs per 4 pl volume. After this normalization, the noise plots reported in Figure 2G and Figure S2B had comparable ranges on the x-axis.

Spatial variation in noise

The CV^2 data from all slices in all embryos in each genetic background was combined as described in the "Amplitude measurement" section. CV^2 values of the *i*th slices from both left and right halves of all embryos were combined. The mean and standard error of CV^2 values were plotted at different spatial positions (Figure 4B).

To control the dependency of spatial CV^2 values on expression levels, we carried out two further analyses:

1) The data were grouped into three categories depending on its expression level: low, medium and high. Slices were first ranked from the highest to the lowest gene expression levels. Then, they were divided into three equally sized groups: bottom 33.33% (low expression), middle 33.33% and top 33.33% (high expression). For each spatial location, three different CV^2 values (at low, medium and high mRNA levels) and their standard errors were calculated by using the sum of *her1* and *her7* mRNA levels at each genetic background (wild-type, *deltaC*$^{-/-}$ and *deltaD*$^{-/-}$; Figure S4A).

2) The embryos' slices are placed into 8 groups based on their mean total *her* expression levels. Slices with expression levels from 0 to 15 formed the first group, and slices from 105 to 120 formed the last group. The slices above 120 are considered to be outliers, and eliminated from the analysis. Afterwards, for each expression group, the data coming from slices located in the posterior 33% and anterior 33% of the PSM are selected. Then, the mean plus two standard error of expression level, and mean plus two standard error of CV^2 in posterior and anterior slices are calculated. The figure summarizing this analysis is provided in Figure 4C, D.

Statistical analysis

One-way ANOVA was conducted to determine the statistical significance of the differences between amplitude and noise in different genetic backgrounds and/or spatial locations. Normality was assessed by visual inspection of histograms and normal Q-Q plots. Homogeneity of variances was assessed by comparing standard deviations of different groups.

Computational Model

We built a computational model to simulate synchronized oscillation in two neighboring cells. In this model, a cell (Cell a) produces a protein Z^a from a constitutive gene as shown in Figure S5A. This protein activates the production of two other proteins X^a and Y^a. The protein X^a inhibits the production of the protein Z^a after a time-delay τ_x, while the protein Y^a activates the production of Z in the neighboring cell (Cell b) after another delay τ_y. The same analogy is true for the neighboring cell. Overall, the production rate of protein Z^a at time t is given by

$$k_z - k_x x^a(t - \tau_x) + k_y y^b(t - \tau_y), \tag{1}$$

where x^a (y^b) denotes the count level of X^a (Y^b) at time $t - \tau_x$ ($t - \tau_y$). Further k_z, k_x, and k_y are basal production rate, negative feedback strength, and positive feedback strength, respectively. Finally, protein Z decays with rate γ.

The biological representation of the aforementioned time-delays is as follows: The time-delays in Figure S5A are implemented by considering that the proteins X and Y will be activated after some time. Hence, we consider that the protein Z^a produces two intermediate molecules X_1^a and Y_1^a. The intermediate molecule X_1^a (Y_1^a) is converted into its active form X_n^a (Y_m^a) after undergoing a series of n (m) first-order conversion reactions with the rate n/τ_x (m/τ_y). Assuming that the timing of first-order reactions are similar and exponentially distributed, the conversion process creates a gamma-distributed delay in the activation of the proteins with mean time-delays τ_x and τ_y. In addition, noise in time-delays quantified by Coefficient of Variation (CV) squared is

$$CV_{\tau_x}^2 = \frac{\langle \tau_x^2 \rangle - \langle \tau_x \rangle^2}{\langle \tau_x \rangle^2} = \frac{1}{n}, \quad CV_{\tau_y}^2 = \frac{\langle \tau_y^2 \rangle - \langle \tau_y \rangle^2}{\langle \tau_y \rangle^2} = \frac{1}{m}, \tag{2}$$

where $\langle . \rangle$ denotes the expected value. The activated protein X^a inhibits the expression of the protein Z^a and the activated protein Y^a enhances the production of protein Z^b in the neighboring cell. It means that now the production rate of protein Z^a is

$$k_z - k_x x_n^a(t) + k_y y_m^b(t), \tag{3}$$

where $x_n^a(t)$ and $y_m^b(t)$ denote the count levels of X_n^a and Y_m^b at time t, respectively. Finally, the protein Z decays with rate γ. The overall model in Cell a consists of the following chemical reactions:

$$\begin{gathered}
\emptyset \xrightarrow{k_z - k_x x_n^a(t) + k_y y_m^b(t)} Z^a, \qquad Z^a \xrightarrow{\gamma} \emptyset, \\
Z^a \xrightarrow{n/\tau_x} Z^a + X_1^a, \\
X_i^a \xrightarrow{n/\tau_x} X_{i+1}^a, \quad i = \{1, \dots, n-1\}, \\
X_n^a \xrightarrow{n/\tau_x} \emptyset, \\
Z^a \xrightarrow{m/\tau_y} Z^a + Y_1^a, \\
Y_i^a \xrightarrow{m/\tau_y} Y_{i+1}^a, \quad i = \{1, \dots, m-1\}, \\
Y_m^a \xrightarrow{m/\tau_y} \emptyset.
\end{gathered} \tag{4}$$

The model in Cell b is identical to that of Cell a.

We run Monte Carlo simulations to test our model numerically. Our code is written based on SSA algorithm (Gillespie, 1976) and we use the software StochKit2 to simulate the model (Sanft et al., 2011). In our code, we checked a wide range of time-delays. We start with time-delays $\tau_x = 12.15\ mins$ and $\tau_y = 24.3\ mins$ and we increased the time-delays up to $\tau_x = 54\ mins$ and $\tau_y = 108\ mins.$, i.e. ≈ 4.5 fold change in time-delays as measured previously (Ay et al., 2014). We considered that $m = n = 10$, which means noise in time-delays is $CV_{\tau_x}^2 = CV_{\tau_y}^2 = 0.1$. Further note that, to maintain the oscillations of two cells in synchrony, we increase the time-delays with the same ratio. The protein degradation rate is $\gamma = 0.2/\ min$, which means the protein halflife is 3.46 $mins$ as measured previously (Ay et al., 2013). Finally, we select the

rates as $k_x = 0.6$ and $k_y = 0.2$, and by changing delay, we also change k_z to keep the mean of protein Z at 55 ± 1.

Figure S5B illustrates that this model can replicate the synchronized oscillations by interplay between intracellular negative feedback and intercellular positive feedback. Our goal is to calculate the noise in a population of synchronized cells, however due to model size, increasing the number of cells results in increasing the complexity of model. Hence, instead of increasing the number of cells in our code, we used the peak values of oscillations in a cell as a proxy of a population of synchronized cells. To do so, after simulating the model using StochKit, we used a MATLAB code to process the data. In this code, we first read the data from the files that StochKit made. In the next step, we smoothened the data to remove the fluctuations contributed from random production and degradation events. The smoothened data was used to find the peak times. The difference between these times gave the period of oscillations. Afterwards, we extracted the peak levels from the unfiltered data associated with these peak times. The expression variability at the peak levels was defined as expression noise. The StochKit and MATLAB codes used in computational modeling are provided in supplementary information.

SUPPLEMENTARY FIGURES and FIGURE LEGENDS:

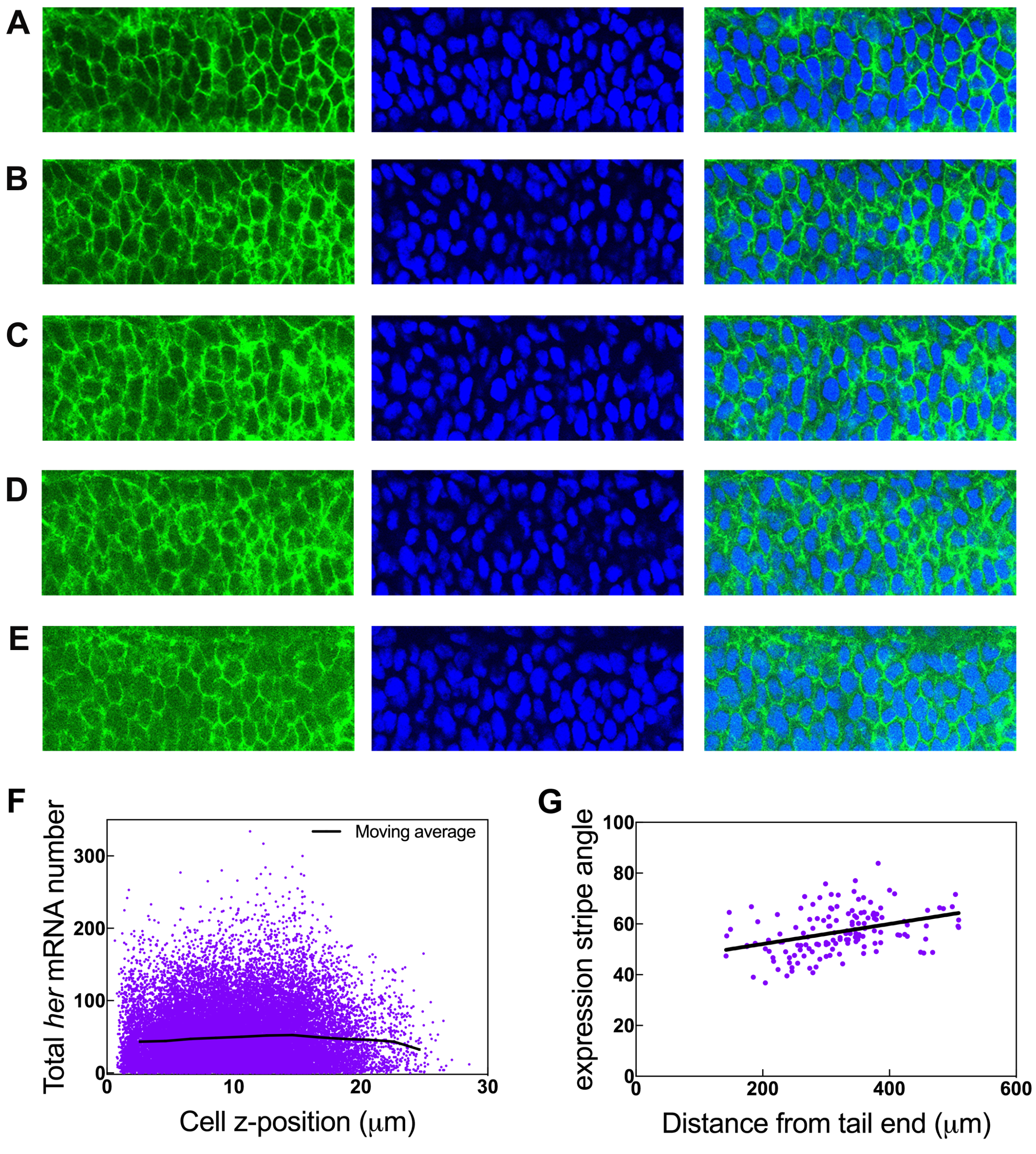

Figure S1. Representative z-sections of an smFISH image and the gradual change in the angle of clock expression stripes, related to Figure 1

(A-E) Different z-sections of an smFISH image show the cell membrane (green), nuclear (DAPI) and merge images in a wild-type embryo, respectively. Images belong to sections 25, 35, 45, 55 and 65 in z-axis, respectively. (F) Total *her* (*her1+her7*) RNA in each cell and the position of each cell are plotted for all cells from all wild-type embryos. Tissue sections further away from the microscopy objective have higher z-values. Successful detection of RNAs does not systematically depend on z-position in microscopy images. The data is first grouped according to z-position with 2μm bin size. Then, a moving average is calculated by using 3 bins. The centered moving average is plotted in the graph. (G) The angle between the clock expression stripes and the AP axis increases incrementally in the posterioanterior direction (away from the tail end of the embryos). The angle data from wild-type embryos is shown here. A linear function ($y = 0.039 * x + 44.23$) fits to the data ($R^2 = 0.14$).

A

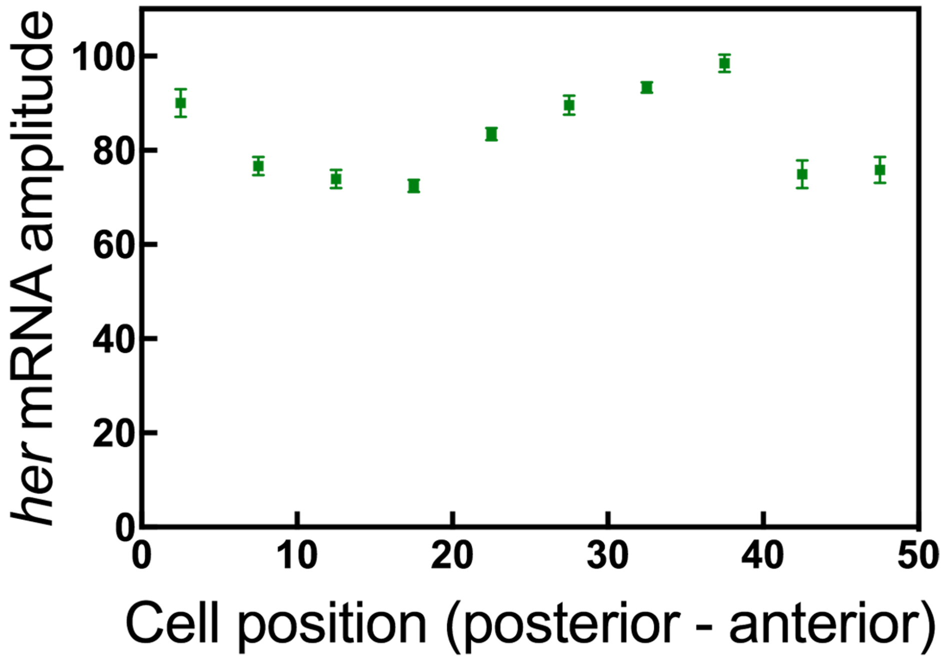

her mRNA amplitude
100
80
60
40
20
0
0
10
20
30
40
50
Cell position (posterior - anterior)

B

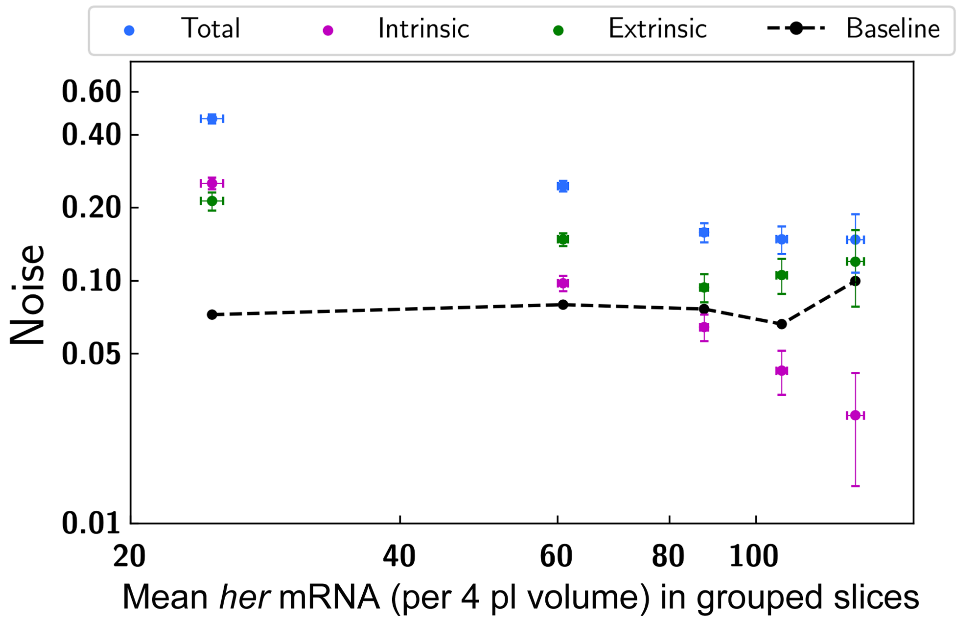

Total
Intrinsic
Extrinsic
Baseline
0.60
0.40
0.20
0.10
0.05
0.01
Noise
20
40
60
80
100
Mean her mRNA (per 4 pl volume) in grouped slices

Figure S2. Spatial profile of total *her* mRNA amplitudes and the effect of cell volume on expression noise, related to Figure 2

(A) Oscillation amplitudes of total *her* RNA (*her1+her7*) do not change drastically along the PSM. Error bars are two standard errors. (B) Differences in cell volumes have a mild contribution in clock gene expression noise. y-axis is noise; x-axis is mean levels of total *her* (*her1+her7*) RNA (per 4 pl volume) in grouped slices.

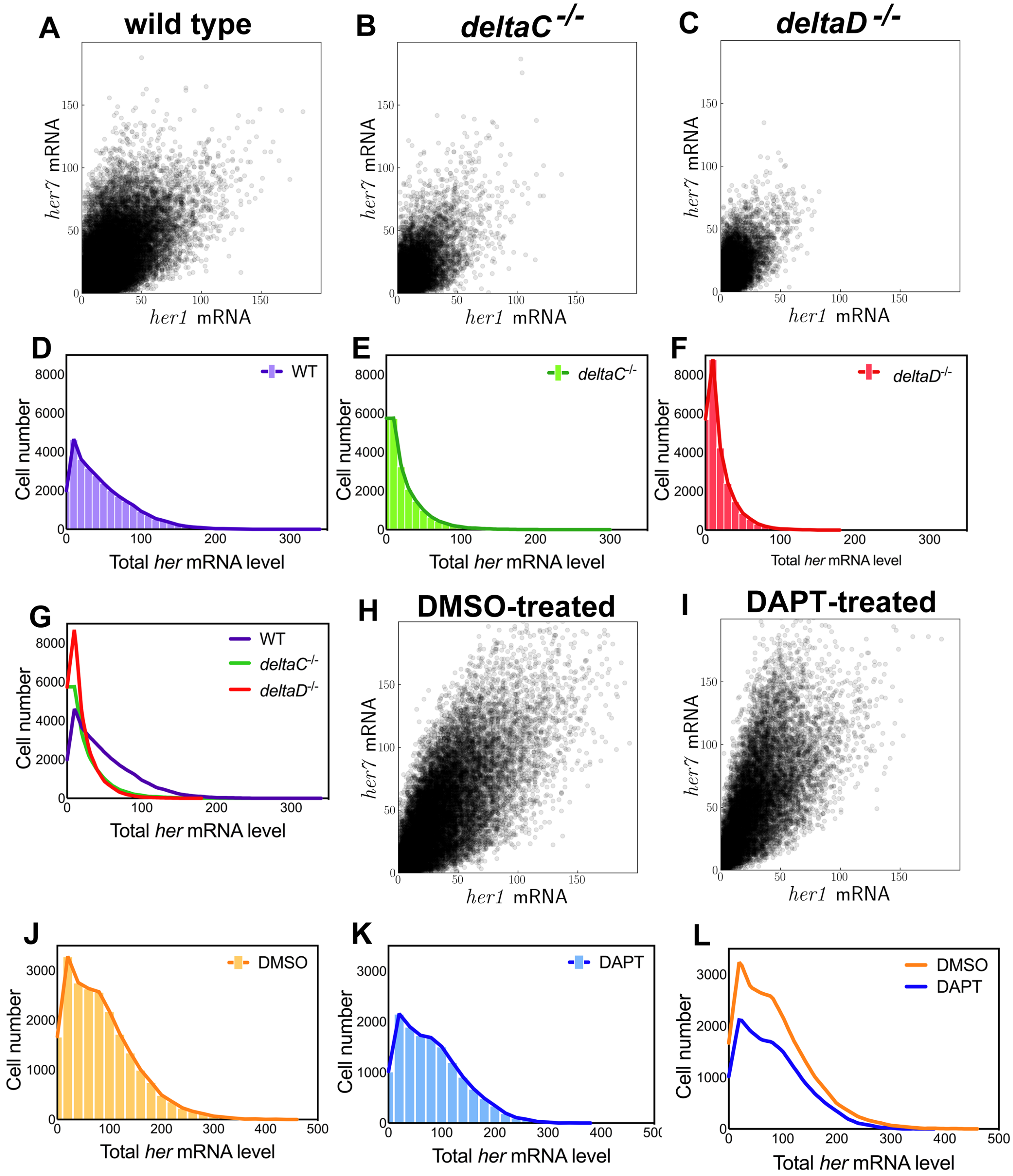

A
wild type
her7 mRNA
her1 mRNA
B
deltaC -/-
C
deltaD -/-
D
WT
Cell number
Total her mRNA level
E
deltaC-/-
F
deltaD-/-
G
WT
deltaC-/-
deltaD-/-
H
DMSO-treated
I
DAPT-treated
J
DMSO
K
DAPT
L
DMSO
DAPT

Figure S3. ***her1*** **versus** ***her7*** **RNA numbers and the distribution of RNA numbers in each cell in different genetic and drug-treated backgrounds, related to Figures 2 and 3**

(A-C) Scatter plot of *her1* versus *her7* RNA levels in each cell in all embryos in wild-type (A), *deltaC*$^{-/-}$ (B) and *deltaD*$^{-/-}$ (C) backgrounds, respectively. (D-G) The distribution of cell numbers with a given total *her* (*her1*+*her7*) RNA number in wild-type (D), *deltaC*$^{-/-}$ (E), *deltaD*$^{-/-}$ (F), and overlaid (G) backgrounds, respectively. The data came from 28904 cells from 18 wild-type, 21560 cells from 14 *deltaC*$^{-/-}$ mutant and 24997 cells from 18 *deltaD*$^{-/-}$ mutant embryos. (H, I) Scatter plot of *her1* versus *her7* RNA levels in each cell in all DMSO-treated and DAPT-treated embryos. (J-L) The distribution of cell numbers with a given total *her* (*her1*+*her7*) RNA number in DMSO-treated embryos (J), in DAPT-treated embryos (K) and overlaid (G), respectively. The data came from 14091 cells from 9 DAPT-treated and 21497 cells from 12 DMSO-treated embryos.

A

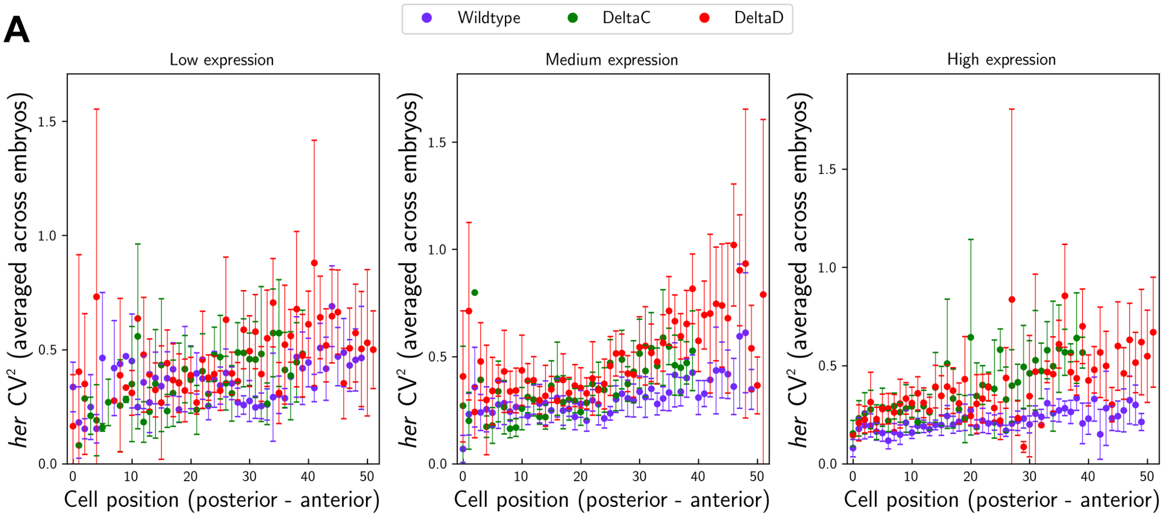
Wildtype
DeltaC
DeltaD
Low expression
Medium expression
High expression
her CV² (averaged across embryos)
Cell position (posterior - anterior)

B

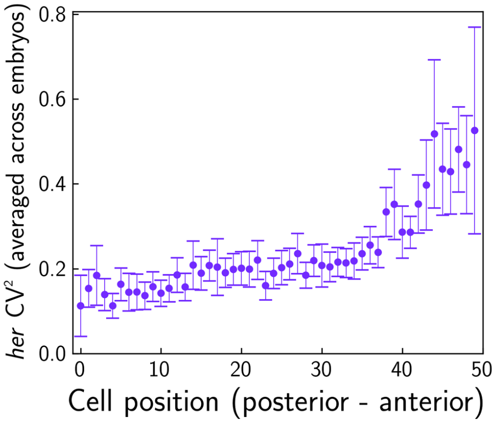
her CV² (averaged across embryos)
Cell position (posterior - anterior)

Figure S4. Expression noise displays an increasing profile along the unsegmented axis, related to Figure 4

(A) Expression noise is analyzed in the PSM for total *her* (*her1* + *her7*) mRNAs in wild-type (purple), *deltaC*$^{-/-}$ (green) and *deltaD*$^{-/-}$ (red) mutant embryos. Data is grouped based on average expression levels (left column for low, middle column for medium and right column for high mRNA). Noise is averaged across all embryos at a given spatial position in each genetic background. X-axis is spatial positions; error bars are two standard errors. (B) The clock gene expression noise displays a spatially increasing profile along the posterioanterior direction in the PSM even when the differences in cell volumes are taken into account.

A

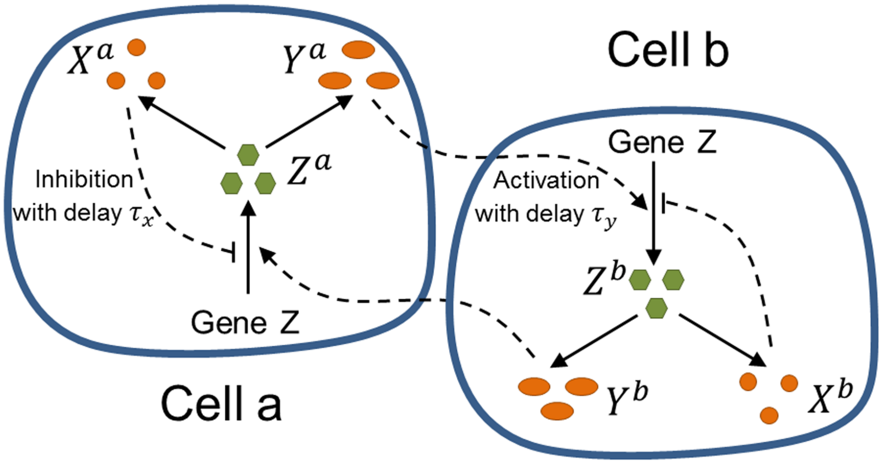

X^a
Y^a
Cell b
Z^a
Gene Z
Inhibition
with delay τ_x
Activation
with delay τ_y
Z^b
Gene Z
Cell a
Y^b
X^b

B

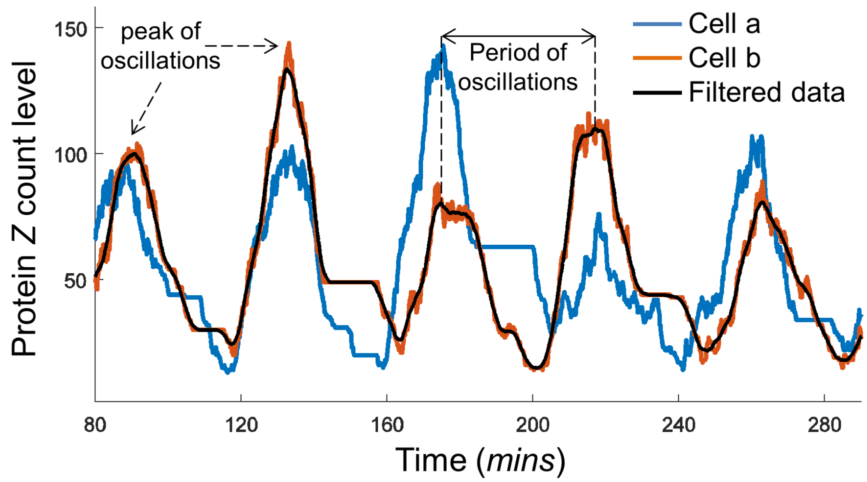

peak of
oscillations
Period of
oscillations
Cell a
Cell b
Filtered data
150
100
50
Protein Z count level
80
120
160
200
240
280
Time (mins)

Figure S5. The stochastic model simulates two coupled cells displaying synchronized oscillations, related to Figure 4

(A) A stochastically expressed protein Z activates production of two other proteins X and Y. The protein X inhibits the production of the protein Z in its own cell after a time delay. The protein Yactivates the production of the protein Z in the neighboring cell after another time delay. (B) Time trend of two cells oscillating in synchrony is used to calculate the noise and the period of oscillations. The period of oscillations is calculated by looking at the time intervals in which protein level in 'Cell b' reaches to its peak. The noise in protein is calculated for the points in which the protein level in 'Cell b' is at the peak of oscillations.

SUPPLEMENTARY TABLE:

**Table S1.**	***her1***		***her7***	
	Mean	Variance	Mean	Variance
**wildtype**	3.1	15.3	1.3	3.6
deltaC	3.9	12.7	1.3	2.6
deltaD	2.8	8.8	1.3	2.4
**DAPT**	4.4	3.0	1.0	1.3
**DMSO**	4.7	3.3	2.7	4.1

Table S1. Mean and variance of nonspecific smFISH staining, Related to Figure 1
