# Supplementary material for: Noise in the Vertebrate Segmentation Clock Is Boosted by Time Delays but Tamed by Notch Signaling
Source: Cell Rep. Author manuscript; Available in PMC 2018 Jun 6. (PMC5989725; doi:10.1016/j.celrep.2018.04.069)
Supplement: 2 [file NIHMS970664-supplement-2.zip › analysis_pipeline/ImageProcessingPipeline.docx]

**Image Processing Pipeline**

Pipeline described below is created for analyzing single molecule counting data coming from embryos in different genetic backgrounds. Python and MATLAB scripts are in bold and input and output files are italicized. Input and output Excel files shared across the scripts are color-coded. All figures are created with dpi of 300.

Below is the file structure of this package:

|__source: Source code for this package. Any “.py” or “.m” file should be stored here.

|__wildtypefulldataset

|______input: where experimental data files (named in the format: WT<embryo_index>.xlsx) are placed.

|______output:

|__________embryo<embryo_index>: folders containing the results of analysis of each individual embryo.

|__________All files containing the results of analysis that combine all embryos we collected in wild type genetic background.

|__deltacfulldataset: input and output files for analysis in deltaC^-/-^ genetic background. Same structure as wildtypefulldataset

|__deltadfulldataset: input and output files for analysis in deltaD^-/-^ genetic background. Same structure as wildtypefulldataset

|__DAPT: input and output files for analysis in DAPT genetic background. Same structure as wildtypefulldataset

|__DMSO: input and output files for analysis in DMSO genetic background. Same structure as wildtypefulldataset

|__compare_output: where output file of analysis that compare the behaviours across genetic backgrounds

Whenever you try to all input to this program, please follow this structure and add data in the right place in order to avoid the program crashing or not doing the proper analysis.

In this package, we used the following:

- Python 2.7.10
- Matlab 2016, stored in /Applications/MATLAB_R2016a.app/bin/matlab
- Python modules: [scipy](https://www.scipy.org/install.html), numpy (included in scipy), [xlrd](https://pypi.python.org/pypi/xlrd), [xlwt](https://pypi.python.org/pypi/xlwt), [matplotlib](https://matplotlib.org/users/installing.html)
- LaTeXiT

If you are analyzing a new set of embryos, you can either use the command-line arguments to directly run the Python and MATLAB scripts below or write a separate Python script like wildtype_analysis.py, deltac_analysis.py, or deltad_analysis.py that calls the scripts in order as explained below. We provided you with these files; if you add/delete/modify experimental data, you may want to look at these files and change accordingly.

**wildtype_analysis.py** (or **wildtype_volumcorrection.py** or **deltac_analysis.py** or **deltad_analysis.py** or **DAPT.py,** or **DMSO.py**)

- Calls other Python scripts to analyze all embryos in a given genetic background
- The following input values need to be specified at the top of the script. These values are fixed throughout our study, therefore, we did not allow them to be specified through command-line arguments. If you change these values in your study, you can change them in the code of wildtype_analysis.py; the code is thoroughly commented.
  - number of embryos to analyze (num_embryos)
  - background noise mean for her1 and her7 (CB, YB) and variance for her1 and her7 (VARCB, VARYB)
    - wild-type: CB = 3.08345, VARCB = 15.27150, YB = 1.31066, VARYB = 3.5518
    - deltaC: CB = 3.89040, VARCB = 12.71174, YB = 1.30271, VARYB = 2.59448
    - deltaD: CB = 2.82650, VARCB = 8.75354, YB = 1.28442, VARYB = 2.35525
  - name of the folder for all input and output files (folder) --we assume that all raw experimental data files to be in input folder; all result figures and Excel files will be saved in output folder
- The following scripts are called to analyze the embryos:

1. **embryo_analysis.py** for each embryo
   - Takes raw experimental data of an embryo and creates two Excel files containing data needed for future analyses
   - input: Excel file containing raw experimental data of an embryo (e.g. *WT6.xlsx*)
   - output: *slices.xls, cells.xls ,* *sliceInfo .xls* (inside ./wildtypefulldataset/output/embryo<embryo_index> folder)
   - command-line argument format: python embryo_analysis.py -i <input Excel file> -d <output directory> -l1 <left region first angle> -l2 <left region second angle> -r1 <right region first angle> -r2 <right region second angle> -n <number of sections> -f <0 or 1 to specify input format> -s <half threshold shift>
     1. angles: angels for slicing the embryos. We use angle measurement function y intercept as right angle, left angel=180-right angle(angle measurement function is generated by measuring all embryo clock left/right expression angle(<90°) and linear fit the date points, Figure S1g). For mutant backgrounds such as deltaC and deltaD whose expression angle are lost we use WT expression angle as input.
     2. number of sections: The Excel file containing raw experimental data is divided into multiple (e.g. four) sections. Open the Excel file and count the number of sections (defined in the first row).
     3. input format: In some cases (mostly if the data is new), embryo data needs to be split into left and right and shifted along the x-axis so that the sections do not overlap. Open each input Excel file and determine whether the embryo has been divided into left and right regions. If the file does not specify whether a section is in the left or right region, it means the data has not been split in half and shifted yet. If this is the case, write “-f 0”. If the file does specify whether a section is in the left or right region, write “-f 1”.
     4. half threshold shift (optional): If the data has not been split in half (i.e. you wrote “-f 0”) and the midpoint of the given data does not accurately split the embryo in half, we can specify how much we want to shift the threshold for left and right. For example, write -20 to shift the threshold to the right by 20 and write 10 to shift the threshold to the left by 10. If there is no need to shift the threshold, you can omit this option (because the default threshold is 0) or write “-s 0”. You may create the embryo’s heatmap (using create_heatmap.py) to gauge how much you want to shift the threshold.
     5. Left_axis_shift (-ly): Some mutants require the left section to be shifted along the y-axis. This specifies how much shift is needed. This parameter is only used in analyzing wild type embryos.
     6. Middle-section: wild-type embryos may contain middle sections. This parameter specifies how many middle sections are in each embryo.
   - example: python embryo_analysis.py -i ../wildtypefulldataset/input/new_WT1.xls -d ../wildtypefulldataset/output/embryo1 -a 44.23 -dA 0.039 -n 6 -f 0 -s -20
   - raw data format: The first line contains header information. 6 columns together contain data for one section and sections are laid out horizontally. Within a section, each line corresponds to a single cell. Second, third, and fourth columns correspond to cell position coordinates (x,y,z). Fifth and sixth columns correspond to the number of *her1* and *her7* mRNA molecules detected within the cell. Sections may contain different number of cells, resulting in different number of rows.
   - **note:** This script needs regions.py and slices.py (both written by us) in the source folder. Different embryos may require different command-line arguments because of their different format or number of sections. This is the only time we read the raw experimental data. The following scripts use the output files of this script, *cells.xls* and *slices.xls*. Background noise subtraction is not done here because some scripts filter the data differently. If a slice has fewer than three cells, we ignore the slice and write “Too few cells to analyze” to the *slice.xls*. **Fix angle analysis:** For angle input in embryo_analysis.py you can measure (counterclockwise from the positive x-axis) and give different expression angle for each embryo, and use the same angle to slices the whole tissue, in this case set delta_angle to 0, and give a pseudo value for ‘-a’.
2. **create_heatmap.py** for each embryo
   - Creates two heat maps for an individual embryo by drawing slice boundaries and plotting high expression cells with bold colors and low expression cells with light colors
   - input: *cells.xls,* *sliceInfo.xls*
   - output: *heatmap_her1.png, heatmap_her7.png*
   - command-line argument format: python create_heatmap.py -i <input Excel file> -d <output directory>
   - example: python create_heatmap.py -i ../wildtypefulldataset/output/embryo1/cells.xls -s ../wildtypefulldataset/output/embryo1/sliceInfo.xls -d ../wildtypefulldataset/output/embryo1
   - paper figure: *heatmap_her7.png* of wild-type embryo 2 is paper figure 1f.
   - note: Threshold for low vs. high expression is: minimum+0.3*(maximum-minimum). For wild-type embryos, make sure the slice boundaries are parallel to the observed stripe patterns shown in high expression cells. Examining the heatmaps is a good opportunity to make sure your input data was processed properly in embryo_analysis.py. After examining the heatmaps, you may need to rerun embryo_analysis.py to adjust the half threshold shift or other input values. This script may take more time than other scripts to finish running.
3. **plot_spatial_expression.py** for each embryo
   - Plots mRNA expression across space for an individual embryo (cell position 0 corresponding to posterior)
   - input: *slices.xls*
   - output: *left_spatial_expression.png, right_spatial_expression.png, left_raw_her1.png, left_raw_her7.png, right_raw_her1.png, right_raw_her7.png, spatial_expression.xls*
   - command-line argument format: python plot_spatial_expression.py -i <input Excel file> -d <output directory> -m1 <her1 background noise mean> -m7 <her7 background noise mean>
   - example: python plot_spatial_expression.py -i ../wildtypefulldataset/output/embryo1/slices.xls -d ../wildtypefulldataset/output/embryo1 -m1 3.083 -m7 1.311
   - paper figure: *left_raw_her7.png* of wild-type embryo 2 is paper figure 1g.
4. **combine_embryos.py** combining all embryos
   - Calls 9 Python and MATLAB scripts that combine data from all embryos.
   - input: all 20 *slices.xls* files
   - output: figures and Excel files (explained below)
   - command-line argument format: python combine_embryos.py -ne <number of embryos> -nb<number of bins to calculate binned noise> -d <output directory> -m1 <her1 background noise mean> -m7 <her7 background noise mean> -v1 <her1 background noise variance> -v7 <her7 background noise variance> -i <first embryo's slice.xls> <second embryo's slice.xls> ... <last embryo's slice.xls>
   - example: python combine_embryos.py -ne 20 -d ../wildtypefulldataset/output –nb 5 -m1 3.083 -m7 1.311 -v1 15.27150 -v7 3.5518 -i ../wildtypefulldataset/output/embryo1/slices.xls ../wildtypefulldataset/output/embryo2/slices.xls …/ wildtypefulldataset/output/embryo20/slices.xls
   - note: embryo_analysis.py must be called for each embryo before running this script. MATLAB will automatically start running during this script. The line opening MATLAB in this script may have to change based on which MATLAB version (default: R2016a) is available or where MATLAB is located (default: /Applications/MATLAB_R2016a.app/bin/matlab). Running this script should be the last step for analyzing embryos of a given genetic background. If you wish to run these scripts individually, the order shown below is recommended since some scripts are dependent on each other for input and output.
   - Below are the 6 scripts:
     1. **plot_spatial_amplitude.py**
        - Combines spatial expression data and calculates spatial amplitude of oscillations in gene expression
        - Plots:
          1. bar graph showing her1 and her7 average spatial amplitudes *(average_spatial_amplitude_bar.png)*
          2. line graph showing cell position vs. amplitude, combining both regions of all embryos at every five spatial locations *(spatial_amplitude_line.png)*
          3. cell position vs. expression level combining both regions of all embryos at each spatial location *(combined_spatial_expression.png)*
        - input: all 20 *slices.xls* files
        - output: *average_spatial_amplitude_bar.png, spatial_amplitude_line.png, combined_spatial_expression.png, spatial_amplitude.xls, combined_spatial_expression.xls*
        - command-line argument format: python plot_spatial_amplitude.py <number of embryos> <her1 background noise mean> <her7 background noise mean> <first embryo's slice.xls> <second embryo's slice.xls> ... <last embryo's slice.xls> <output directory>
        - example: python plot_spatial_amplitude.py 20 3.083 1.311 ../wildtypefulldataset/output/embryo1/slices.xls ../wildtypefulldataset/output/embryo2/slices.xls ... ../wildtypefulldataset/output/embryo20/slices.xls ../wildtypefulldataset/output
        - paper figure: *average_spatial_amplitude_bar.png* of wild-type embryos is paper figure 2b, figure 3 in *spatial_amplitude_line.png* is paper figure S2.
        - note: In addition to plotting amplitude, this script also plots spatial expression (*combined_spatial_expression.png*) from data combining all embryos to demonstrate and confirm where the amplitude values are calculated from.
     2. **plot_noise.py**
        - Calculates intrinsic, extrinsic, and total noise levels of each slice and plots mean expression vs. noise levels using four x-axes (her1, her7, her, and harmonic mean)
        - input: all 20 *slices.xls* files
        - output: *noise_her.png, noise_her1.png, noise_her7.png, noise_Harmonic mean.png, logNoise_her.tiff, logNoise_her1.tiff, logNoise_her7.tiff, logNoise_Harmonic mean.tiff, loglogHervsNoise_her.png, noise.xls,* combined_slices.xls
        - command-line argument format: python plot_noise.py <number of embryos> <number of bins> <her1 background noise mean> <her7 background noise mean> <first embryo's slice.xls> <second embryo's slice.xls> ... <last embryo's slice.xls> <output directory>
        - example: python plot_noise.py 20 3.083 1.311 ../wildtypefulldataset/output/embryo1/slices.xls ../wildtypefulldataset/output/embryo2/slices.xls … ../wildtypefulldataset/output/embryo20/slices.xls ../wildtypefulldataset/output
        - paper figure*: loglogHervsNoise_her.png* is figure 2f; *logNoise_her.png* of wild-type embryos is paper figure 2g.
        - **Note**: Number of bins used in these figures are fixed, and hard input in code. But we also have function for flexible number of bins in the code. It can be 5 (what we used to use) or any other number. Number of bins is specified in command-line arguments to combine_embryos.py. If want to use this function, the noise.xls export need to make change accordingly.

We also included background noises in these plots, to represent the scale of intrinsic and extrinsic noises compared to background noises in the system. Background noises for all bins are hard-coded in background_bin variable (on top of scripts in **plot_noise.py**). Any changes in background noise levels/number of bins and bins cut off should be addressed this part of the code.

- - 1. **plot_scatter_her1_her7.py**
       - Create scatter plots of *her1* vs. *her7* mRNA levels in all cells of all embryos in each genetic backgrounds. These plots can be created using binned data calculated in plot_noise.py (above), or using all data we collected of all cells. These plots will show the correlation behaviours of *her1* and *her7* gene expression levels.
       - Input: combined_slices.xls
       - Output: her1_her7_bin<bin_index: 1-5>.png, her1_her7_all.png
       - Command-line argument format: python plot_scatter_her1_her7.py <inputFile: combined_slices.xls-- result of plot_noise.py> <number of bins> <output directory to store the plots>
       - Example: python plot_scatter_her1_her7.py ../wildtypefulldataset/output/combined_slices.xls 5 ../wildtypefulldataset/output
       - Paper figure: figure *her1_her7_all.png* for wild type is figure S3 in the paper
    2. **plot_CVsquared.py**
       - Plots spatial noise
       - input: all 20 *slices.xls* files
       - output: *CVsquared_her1.png, CVsquared_her7.png, CVsquared_grouped_her.png, binned_cv_her_heatmap.png, CVsquared.xls*
       - command-line argument format: python plot_CVsquared.py <number of embryos> <her1 background noise mean> <her7 background noise mean> <first embryo's slice.xls> <second embryo's slice.xls> ... <last embryo's slice.xls> <output directory>
       - example: python plot_CVsquared.py 20 3.083 1.311 ../wildtypefulldataset/putput/embryo1/slices.xls ../wildtypefulldataset/output/embryo2/slices.xls ... ../wildtypefulldataset/output/embryo20/slices.xls ../wildtypefulldataset/output
       - paper figure: *binned_cv_her_heatmap.png*  for wild type is paper figure 4D.
    3. **create_raw_expression_excel.py**
       - Creates two Excel files containing mRNA expression levels of each slice before and after background subtraction
       - input: all 20 *slices.xls* files
       - output: *raw_expression_beforebackgroundsub.xls, raw_expression_afterbackgroundsub.xls*
       - command-line argument format: python create_raw_expression_excel.py <number of embryos> <her1 background noise mean> <her7 background noise mean> <first embryo's slice.xls> <second embryo's slice.xls> ... <last embryo's slice.xls> <output directory>
       - example: python create_raw_expression_excel.py 20 3.083 1.311 ../wildtypefulldataset/output/embryo1/slices.xls ../wildtypefulldataset/output/embryo2/slices.xls ... ../wildtypefulldataset/output/embryo20/slices.xls ../wildtypefulldataset/output
       - note: No figures are created. No data elimination is done here. Negative expression levels are shown.
    4. **create_raw_noise_excel.py**
       - Creates an Excel file containing noise levels for each slice in each embryo
       - input: all 20 *slices.xls* files
       - output: *raw_noise.xls*
       - command-line argument format: python create_raw_noise_excel.py <number of embryos> <her1 background noise mean> <her7 background noise mean> <first embryo's slice.xls> <second embryo's slice.xls> ... <last embryo's slice.xls> <output directory>
       - example: python create_raw_noise_excel.py 20 3.083 1.311 ../wildtypefulldataset/output/embryo1/slices.xls ../wildtypefulldataset/output/embryo2/slices.xls ... ../wildtypefulldataset/output/embryo20/slices.xls ../wildtypefulldataset/output
       - note: No figures are created. Blanks are written for invalid data.

**compare_geneticbackgrounds.py**

- Once the analysis for each genetic background is done as explained above, this script can be run to combine the data from different genetic backgrounds for comparison.
- Flexibility: This program is usually used to compare analysis results for 3 genetic backgrounds (wild-type, deltaC, and deltaD), but more genetic backgrounds can be added. Figure size may increase to accommodate larger data and figure legends may need to be edited to fit more than three symbols/names. The mutations data will normalized to Wildtype data.
- command-line argument format: python compare_geneticbackgrounds.py <number of genetic backgrounds> <number of bins for noise plots> <output_directory> <folders containing input files for each genetic background> <genetic background names for labeling> <genetic background colors for plotting>
- example: python compare_geneticbackgrounds.py 3 5 ../compare_output/WTdeltaCdeltaD ../wildtypefulldataset/output/ ../deltacfulldataset/output/ ../deltadfulldataset/output/ Wildtype DeltaC DeltaD \#722AFF g r
- note: All figures and Excel files will be saved in ./compare_output folder. Number of bins for noise plots should match the one from plot_noise.py (in our study: 5).
- The following scripts are called:

1. **compare_spatial_amplitude.py** can run only after running **plot_spatial_amplitude.py** for all genetic backgrounds
   - Creates a figure comparing *her1* and *her7* amplitudes in different genetic backgrounds.
   - input: three *spatial_amplitude.xls* from three genetic backgrounds
   - output: *compare_spatial_amplitude.png, compare_spatial_amplitude.xls*
   - command-line argument format: python compare_spatial_amplitude.py <number of genetic backgrounds> <output directory> <spatial_amplitude.xls from each genetic backgrounds> <genetic background names for labeling> <genetic background colors for plotting>
   - example: python compare_spatial_amplitude.py 3 ../compare_output/WTdeltaCdeltaD ../wildtypefulldataset/output/spatial_amplitude.xls ../deltacfulldataset/output/spatial_amplitude.xls ../deltadfulldataset/output/spatial_amplitude.xls Wildtype DeltaC DeltaD \#722AFF g r
   - note: Figure width will increase/decrease if you increase/decrease the number of genetic backgrounds. However, the figures may not be as beautifully aligned when you input number of genetic backgrounds other than 2 or 3.
2. **compare_noise.py** can run only after running **plot_noise.py** for all genetic backgrounds
   - Creates a figure comparing noise levels in different genetic backgrounds
   - input: three *noise.xls* from three genetic backgrounds
   - output: *compare_noise.png, compare_noise_log.png, compare_total_noise_log.png, compare_intrinsic_noise_log.png, compare_extrinsic_noise_log.png*
   - command-line argument format: python compare_noise.py <number of genetic backgrounds> <output_directory> <number of bins used in plot_noise.py> <noise.xls from each genetic backgrounds> <genetic background names for labeling> <genetic background colors for plotting>
   - example: python compare_noise.py 3 5 ../compare_output ../wildtypefulldataset/WTdeltaCdeltaD output/noise.xls ../deltacfulldataset/output/noise.xls ../deltadfulldataset/output/noise.xls Wildtype DeltaC DeltaD \#722AFF g r
   - paper figure: *compare_intrinsic_noise_log.png, compare_extrinsic_noise_log.png* for WT/DeltaC/DeltaD is paper figure 3c, 3d; *compare_intrinsic_noise_log.png, compare_extrinsic_noise_log.png* for DAPT/DMSO is paper figure 3g, 3h.
   - note: Number of bins should match the one from plot_noise.py. If the number of slices in a bin is less than 3 slices, compare_noise.py will not plot that bin.
3. **compare_noise_bar.py** can run only after running **create_raw_noise_excel.py** for all genetic backgrounds
   - Creates bar figured comparing average noise levels in different genetic backgrounds
   - input: three *raw_noise.xls* from three genetic backgrounds
   - output: *compare_raw_noise_bar.png, compare_nor_noise_bar.png, compare_noise_bar.xls*
   - command-line argument format: python compare_noise_bar.py <number of genetic backgrounds> <output directory> <raw_noise.xls from each genetic backgrounds> <genetic background names for labeling> <genetic background colors for plotting>
   - example: python compare_noise_bar.py 3 ../compare_output ../wildtypefulldataset/output/raw_noise.xls ../deltacfulldataset/output/raw_noise.xls ../deltadfulldataset/output/raw_noise.xls Wildtype DeltaC DeltaD \#722AFF g r
   - note: No Excel file created. Figure width will increase if you increase the number of genetic backgrounds.
4. **compare_grouped_CVsquared.py** can run only after running **plot_CVsquared.py** for all genetic backgrounds
   - Creates a figure comparing CV^2^ values in low, medium, and high expression slices in different genetic backgrounds
   - input: three *CVsquared.xls* from three genetic backgrounds
   - output: *compare_grouped_CVsquared.png, compare_grouped_CVsquared_her.xls*
   - command-line argument format: python compare_grouped_CVsquared.py <number of genetic backgrounds> <output_directory> <CVsquared.xls from each genetic backgrounds> <genetic background names for labeling> <genetic background colors for plotting>
   - example: python compare_grouped_CVsquared.py 3 ../compare_output ../wildtypefulldataset/output/CVsquared.xls ../deltacfulldataset/output/CVsquared.xls ../deltadfulldataset/output/CVsquared.xls Wildtype DeltaC DeltaD \#722AFF g r
   - paper figure: *compare_grouped_CVsquared.png* is paper figure S5.
   - note: Figure legend will have to be adjusted if you have more than three genetic backgrounds.
5. **compare_CVsquared.py** can run only after running **plot_CVsquared.py** for all genetic backgrounds
   - Creates a figure comparing CV^2^ values in different genetic backgrounds
   - Input: three *CVsquared.xls* from three genetic backgrounds
   - output: *compare_CVsquared.png, compare_CVsquared_her.png, compare_CVsquared.xls*
   - command-line argument format: python compare_CVsquared.py <number of genetic backgrounds> <output_directory> <CVsquared.xls from each genetic backgrounds> <genetic background names for labeling> <genetic background colors for plotting>
   - example: python compare_CVsquared.py 3 ../compare_output ../wildtypefulldataset/output/CVsquared.xls ../deltacfulldataset/output/CVsquared.xls ../deltadfulldataset/output/CVsquared.xls Wildtype DeltaC DeltaD \#722AFF g r
   - paper figure: *compare_CVsquared_her.png* is paper figure 4b.
   - note: Unlike compare_grouped_CVsquared.py, this does not divide the slices into low, medium, and high expression groups. Figure legend will have to be modified if you have more than three genetic backgrounds.

**compareDAPTandDMSO.py**

- This function will do exactly the same things as compare_geneticbackgrounds.py. besides using DMSO instead of WT data to normalize result. It compares all noise and oscillation features from DAPT and DMSO genetic backgrounds; all output excel files and figures will be stored inside ./compare_output/DAPTandDMSO. You can always do everything that this function does by calling the following command:

```

python compare_geneticbackgrounds.py 2 5 ../compare_output/DAPTandDMSO ../DAPT/output ../DMSO/output DAPT DMSO “#0000FF” “#FA5858”

```

This program is created solely to help us reduce the amount of typing we have to do when we call compare_geneticbackgrounds.py using the above command.

- Command-line argument format: python comareDAPTandDMSO.py <number of bins used to calculate binned noise>
- Example: python compareDAPTandDMSO.py 5

**plot_normalized_five_backgrounds.py**

- This program creates plots that compare the level of noises and spatial amplitudes of all five genetic backgrounds: wildtype, deltaC, deltaD, DAPT, DMSO. Wildtype, deltaC, deltaD ‘s data are all normalized based on wild type data; DAPT and DMSO’s data are all normalized based on DMSO’s data. The normalized noise levels and spatial amplitude levels of all these five genetic backgrounds will be plotted into one plot.
- Input: raw_noise.xls from each genetic background, spatial_amplitude.xls from each genetic background
- Output: normalized_herAmp_5bg.png, normalized_herAmp_five_backgrounds.xls, normalized_noise_5bg.png, normalized_noise_five_backgrounds.xls
- Command-line argument format: python compare_geneticbackgrounds.py <number of genetic backgrounds (should be 5)> <output directory> <path to input files for each genetic background> <genetic background names for labeling> <genetic background colors for plotting>
- Example: python plot_normalized_five_backgrounds.py 5 ../compare_output ../wildtypefulldataset/output/ ../deltacfulldataset/output/ ../deltadfulldataset/output/ ../DMSO/output/ ../DAPT/output/ Wildtype DeltaC DeltaD DMSO DAPT '#722AFF' g r '#FA5858' '#0000FF'
- Paper figure: *normalized_herAmp_5bg.png* is figure 3b.

Helpful links for plotting, reading, and writing data:

- Matplotlib pyplot api: <http://matplotlib.org/api/pyplot_api.html>
- Matplotlib pyplot tutorial: <http://matplotlib.org/users/pyplot_tutorial.html>
- xlrd: <https://blogs.harvard.edu/rprasad/2014/06/16/reading-excel-with-python-xlrd/>
- xlwt: <http://www.blog.pythonlibrary.org/2014/03/24/creating-microsoft-excel-spreadsheets-with-python-and-xlwt/>
  - Cannot write more than 256 columns

**Data hierarchy**

Genetic background (wild-type, deltaC, deltaD, DAPT, DMSO) > embryo > region (left, right) > slice > cell > mRNA molecule (*her1*, *her7*).

**Data processing**

We filter our data twice. First, we discard slices with fewer than three cells (in embryo_analysis.py). Second, we perform background noise subtraction (explained below).

**Background noise subtraction**

Background noise mean for *her1* and *her7* (CB, YB) are subtracted from each cell’s *her1* and *her7* gene expression levels. After background subtraction, we discard cells with negative expression levels in either *her1* or *her7*. Since variance in gene expression among cells within a slice is needed for calculating noise levels, we then discard slices with fewer than three cells after background noise subtraction. Unlike the rest of the analysis, when analyzing spatial expression or amplitude (plot_spatial_amplitude.py, plot_spatial_expression.py), negative expression level of any cell is set to zero and not discarded.

**Statistical analysis**

Many (but not all) of the Excel output files written contain formatted data for one-way ANOVA statistical analysis and Tukey post hoc test. Some of them have sheets specifically titled ‘spss_onewayANOVA’. Most of the time all you need to do is copy and paste two columns of data from the Excel file to SPSS or MATLAB. Columns on the left usually contains group numbers (explained below) so that SPSS or MATLAB knows which group the data belongs to. Columns on the right usually contains the actual data (noise level, amplitude, etc).

- Group numbers
  - Noise type: 1: intrinsic noise, 2: extrinsic noise
  - Regions: 1: posterior, 2: anterior
  - Genetic backgrounds: 1: wild-type, 2: deltaC, 3: deltaD or 1: DMSO, 2: DAPT
  - Expression groups: 1:low expression, 2: high expression
- Helpful links:
  - One-way ANOVA and Tukey post hoc test in SPSS: <https://statistics.laerd.com/premium/spss/owa/one-way-anova-in-spss.php>
  - One-way ANOVA and Tukey post hoc test in MATLAB (specify the multiple comparsion test to be Tukey): <http://www.mathworks.com/help/stats/anova1.html>

**Logarithmic scale**

Because the noise levels we plot are so close to zero, matplotlib’s [log or symlog scale](http://stackoverflow.com/questions/3305865/what-is-the-difference-between-log-and-symlog/3513150#3513150) does not properly plot the data points and error bars in logarithmic scale. Therefore, in our scripts we manually calculate and plot the log of each data point and error bar. More information on plotting in log scale can be found here: <http://labs.physics.dur.ac.uk/skills/skills/logscales.php>

**Complete list of scripts in alphabetical order (32 in total)**

| Script | Description |
| --- | --- |
| combine_embryos.py | Calls all scripts that combine data from all embryos in a genetic background. Does not combine data from different genetic backgrounds. |
| compare_geneticbackgrounds.py | Calls all scripts that compare and plot data from different genetic backgrounds. |
| compare_CVsquared.py | Plots spatial noise (cell position vs. CV^2^) in different genetic backgrounds. |
| compareDAPTandDMSO.py | Calls all scripts that compare and plot data from 2 genetic backgrounds: DAPT and DMSO |
| compare_grouped_CVsquared.py | Plots spatial noise (cell position vs. CV^2^) from low, medium, and high expression groups in different genetic backgrounds. |
| compare_noise_bar.py | Plots a bar graph comparing average noise in different genetic backgrounds. |
| compare_noise.py | Plots mean expression vs. noise in different genetic backgrounds. |
| compare_spatial_amplitude.py | Plots spatial amplitude in different genetic backgrounds. |
| create_heatmap.py | Plots two heatmaps (*her1* and *her7*) of an embryo. Called for a single embryo, not multiple embryos. |
| create_labeled_noise_diagram.py | Takes the three diagrams generated from create_noise_diagram.py and creates a combined diagram (paper figure 2c) with labels. |
| create_noise_diagram.py | Creates three diagrams showing the effects of intrinsic and extrinsic noise. Not included in the pipeline. Uses random number generator. |
| create_raw_expression_excel.py | Writes gene expression data (before and after background noise subtraction) from all embryos in a genetic background. |
| create_raw_noise_excel.py | Writes noise data from all embryos in a genetic background. |
| deltac_analysis.py | Calls all scripts that analyze and combine data from all deltaC embryos. |
| deltad_analysis.py | Calls all scripts that analyze and combine data from all deltaD embryos. |
| embryo_analysis.py | Processes raw experimental data of an embryo. Called for a single embryo, not multiple embryos. |
| plot_CVsquared.py | Plots spatial noise (cell position vs. CV^2^) based on all embryos in a genetic background. |
| plot_noise.py | Plots mean expression vs. noise in linear and logarithmic scale based on all embryos in a genetic background. |
| plot_normalized_five_backgrounds.py | Plots average noise levels and spatial amplitudes levels from all five genetic backgrounds that we study in this project, with WT, deltaC, deltaD’s data being normalized with the average WT’s levels and DAPT, DMSO’s data being normalized with the average DMSO’s levels |
| plot_spatial_amplitude.py | Combines spatial expression data, calculates spatial amplitude of oscillations in gene expression, and plots spatial amplitude and expression based on all embryos in a genetic background. |
| plot_spatial_expression.py | Plots cell position vs. expression for an embryo. Called for a single embryo, not multiple embryos. |
| regions.py | Defines class Region. Needed for embryo_analysis.py. |
| shared.py | Needed for almost all python scripts. Includes function ‘ensureDir’ that creates an output folder if it doesn’t exist yet and two functions ‘isFloat’ and ‘isInt’ that check if a given string can be converted into a float or an integer. |
| slices.py | Defines class Slice. Needed for regions.py (and therefore embryo_analysis.py). |
| wildtype_analysis.py | Calls all scripts that analyze and combine data from all wild-type embryos. |

**List of paper figures**

| Figure | Corresponding Python script: corresponding output file |
| --- | --- |
| 1f | create_heatmap.py: *heatmap_her7.png* of wild-type embryo 2 |
| 1g | plot_spatial_expression.py: *left_raw_her7.png* of wild-type embryo 2 |
| 2b | plot_spatial_amplitude.py: *average_spatial_amplitude_bar.png* of wild-type embryos |
| 2f | plot_noise.py: *loglogHervsNoise_her.png* of wild-type embryos |
| 2g | plot_noise.py: *logNoise_her.png* of wild-type embryos |
| 3b | plot_normalized_five_backgrounds.py: *normalized_herAmp_5bg.png* of all five genetic backgrounds |

| 3c | compare_noise.py:  *compare_extrinsic_noise_log.png* of wild-type, deltaC, and deltaD embryos |
| --- | --- |
| 3d | compare_noise.py: *compare_intrinsic_noise_log.png* of wild-type, deltaC, and deltaD embryos |
| 3g | compare_noise.py: *compare_extrinsic_noise_log.png* of DAPT and DMSO embryos |
| 3h | compare_noise.py: *compare_intrinsic_noise_log.png* of DAPT and DMSO embryos |
| 4b | compare_CVsquared.py: *compare_CVsquared_her.png* of wild-type, deltaC, deltaD embryos |
| 4d | plot_CV_squared.py: *binned_cv_her_heatmap.png*  for wild type embryos |
| S2a | plot_spatial_amplitude.py: figure 3 in *spatial_amplitude_line.png*  for wild type embryos |
| S2b | wildtype_volumcorrection.py :  *logNoise_her.png* of wild-type embryos |
| S3 | plot_scatter_her1_her7.py: *her1_her7_all.png* of wild-type/deltaC/deltaD embryos |
| S4 | plot_scatter_her1_her7.py: *her1_her7_all.png* of DMSO/DAPT embryos |
| S5a | compare_grouped_CVsquared.py: *compare_grouped_CVsquared.png* for wild type, deltaC^-/-^, deltaD^-/-^ embryos |
| S5b | wildtype_volumcorrection.py *: CVsquared_her7.png* for wild type |

Written in July 2016 by Soo Bin Kwon (skwon94@ucla.edu), updated in July 2017 by Ha Vu ([havu73@mail.g.ucla.edu](mailto:havu73@mail.g.ucla.edu)), updated in Feb 2018 by Qiyuan Hong(Qiyuan.hong@cchmc.org)
