# Supplementary material for: Noise in the Vertebrate Segmentation Clock Is Boosted by Time Delays but Tamed by Notch Signaling
Source: Cell Rep. Author manuscript; Available in PMC 2018 Jun 6. (PMC5989725; doi:10.1016/j.celrep.2018.04.069)
Supplement: 4 [file NIHMS970664-supplement-4.pdf]

Cell Reports

Article

Noise in the Vertebrate Segmentation Clock Is Boosted by Time Delays but Tamed by Notch Signaling

Graphical Abstract

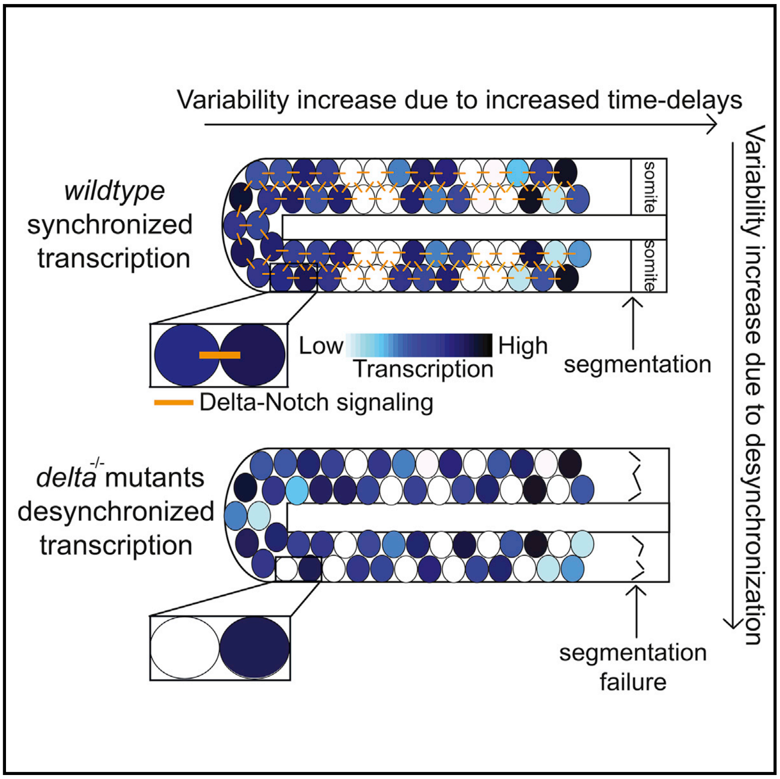

Highlights

- Clock gene transcription levels display low amplitude and high heterogeneity
- Cell-to-cell coupling via Notch signaling tames primarily gene extrinsic noise
- Gene expression noise increases along the posteroanterior axis
- Spatial gradients of time delays contribute to the noise gradient along the axis

Authors

Sevdenur Keskin,
Gnanapackiam S. Devakanmalai,
Soo Bin Kwon, ..., Abhyudai Singh,
Ahmet Ay, Ertuğrul M. Özbudak

Correspondence

ertugrul.ozbudak@cchmc.org

In Brief

Keskin et al. show that segmentation clock transcription levels display low amplitude and high heterogeneity. Clock gene expression variability is primarily driven by gene extrinsic sources, which is suppressed by Notch signaling. Gene expression noise increases along the posteroanterior axis. Spatial gradients of time delays contribute to the noise gradient along the axis.

Keskin et al., 2018, Cell Reports *23*, 2175–2185
May 15, 2018 © 2018 The Author(s).
https://doi.org/10.1016/j.celrep.2018.04.069

CellPress

Cell Reports
Article

OPEN ACCESS
CellPress

Noise in the Vertebrate Segmentation Clock Is Boosted by Time Delays but Tamed by Notch Signaling

Sevdenur Keskin,[1] Gnanapackiam S. Devakanmalai,[1] Soo Bin Kwon,[2] Ha T. Vu,[4] Qiyuan Hong,[5,6] Yin Yeng Lee,[7] Mohammad Soltani,[3] Abhyudai Singh,[3] Ahmet Ay,[4] and Ertuğrul M. Özbudak[1,5,6,8,*]
[1]Department of Genetics, Albert Einstein College of Medicine, Bronx, NY 10461, USA
[2]Department of Computer Science, Colgate University, Hamilton, NY 13346, USA
[3]Department of Electrical and Computer Engineering, Biomedical Engineering and Mathematical Sciences, University of Delaware, Newark, DE 19716, USA
[4]Departments of Biology and Mathematics, Colgate University, Hamilton, NY 13346, USA
[5]Department of Pediatrics, University of Cincinnati College of Medicine, Cincinnati, OH 45229, USA
[6]Division of Developmental Biology, Cincinnati Children's Hospital Medical Center, Cincinnati, OH 45229, USA
[7]Department of Pharmacology and Systems Physiology, University of Cincinnati College of Medicine, Cincinnati, OH 45229, USA
[8]Lead Contact
*Correspondence: ertugrul.ozbudak@cchmc.org
https://doi.org/10.1016/j.celrep.2018.04.069

SUMMARY

Taming cell-to-cell variability in gene expression is critical for precise pattern formation during embryonic development. To investigate the source and buffering mechanism of expression variability, we studied a biological clock, the vertebrate segmentation clock, controlling the precise spatiotemporal patterning of the vertebral column. By counting single transcripts of segmentation clock genes in zebrafish, we show that clock genes have low RNA amplitudes and expression variability is primarily driven by gene extrinsic sources, which is suppressed by Notch signaling. We further show that expression noise surprisingly increases from the posterior progenitor zone to the anterior segmentation and differentiation zone. Our computational model reproduces the spatial noise profile by incorporating spatially increasing time delays in gene expression. Our results, suggesting that expression variability is controlled by the balance of time delays and cell signaling in a vertebrate tissue, will shed light on the accuracy of natural clocks in multi-cellular systems and inspire engineering of robust synthetic oscillators.

INTRODUCTION

Gene expression is inevitably a highly stochastic process due to fluctuations in the complex stoichiometry and reaction kinetics of the biochemical reactions, and it leads to substantial cell-to-cell variability (Balázsi et al., 2011; Elowitz et al., 2002; Kaern et al., 2005; Ozbudak et al., 2002). The resulting phenotypic fluctuations can only be detected and quantified at the single-cell level within isogenic populations. One of the most intriguing questions in science is how developmental pattern formation is executed so robustly despite unavoidable fluctuations in gene expression. This precision necessitates several mechanisms buffering stochastic gene expression. Few studies to date have quantified stochastic gene expression in multi-cellular systems during development (Boettiger and Levine, 2013; Ji et al., 2013; Little et al., 2013; Raj et al., 2010), when buffering the process is critical for the precise and reproducible development of an adult organism, mainly because of technical difficulties posed by quantitative single-cell measurements (Boettiger and Levine, 2013; Ji et al., 2013; Little et al., 2013; Raj et al., 2010).

The study of somitogenesis provides us with an opportunity to investigate the regulation of spatiotemporal precision in pattern formation due to the tight coupling of space and time. The anterior-posterior axis of all vertebrates is patterned as a fixed number of repeating units, the vertebrae. The precursors of vertebrae are derived from somite segments that lay adjacent to the neural tube. Segmentation of somites is dictated by the period of a gene-expression oscillator, called the vertebrate segmentation clock, which is active in unsegmented cells. Oscillatory expression of the *Hes/her* genes is conserved in vertebrates, and disrupting their oscillatory expression results in vertebral segmentation defects (Pourquié, 2011).

The period of the zebrafish segmentation clock is short: 30 min. At the conclusion of each cycle, a cohort of ~200 cells buds from the unsegmented tissue to form a new somite. It remains unknown how segmentation clock factors accumulate to sufficient levels to orchestrate synchronized segmentation of groups of cells. Not only is this process robust and reproducible, but it also undergoes a set number of cycles in a given organism; in zebrafish, segmentation repeats 33 times to form the full-length body axis. Given these robust characteristics, the amplitude of oscillations should be tightly controlled. There is good understanding of the genetic circuitry of the segmentation clock (Pourquié, 2011). However, the field is lacking quantitative measurements of (1) the amplitude of clock gene oscillations, (2) the variability (noise) in clock gene expression between concurrently oscillating cells, (3) how this noise is

This is an open access article under the CC BY license (http://creativecommons.org/licenses/by/4.0/).

OPEN
ACCESS
CellPress

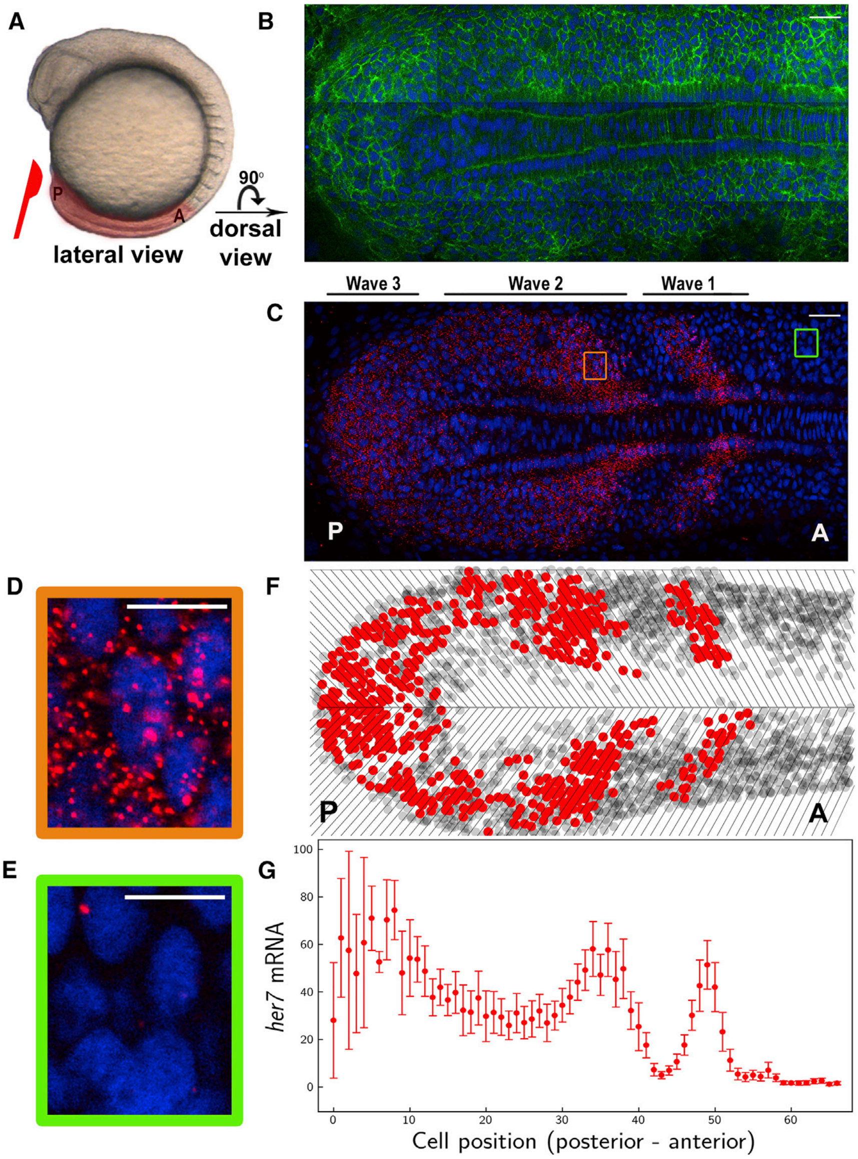

Figure 1. Single RNA Molecules Are Counted in Single-Phase-Binned Cells in the Intact Zebrafish Presomitic Mesoderm

(A) A wild-type zebrafish embryo at the 10-somite stage. PSM, highlighted in pink, is dissected from the embryo. Posterior (P) is to the left, and anterior (A) is to the right.

(B and C) A single z-section of a smFISH picture in a flat-mounted PSM from a wild-type embryo. Cell membrane (green in B), *her7* mRNA (red in C) and nuclear staining (blue in B and C). PSM images are rotated 90 degree compared to that in (A).

(D and E) The images are zoomed on a high-expression stripe (orange square) (D) or a low-expression stripe (green square) (E).

(F) Tissue is divided in single-cell-wide disks along the axis corresponding to different oscillation phases. Cells containing RNA higher or lower than an arbitrary threshold are plotted as red or gray circles, respectively. Top is left half of PSM, and bottom is right half of PSM.

(G) RNA levels from left half of PSM are plotted along the posterior-to-anterior direction. Each dot corresponds to the average RNA number in a spatial phase-binned cell population; error bars indicate 2 SEs.

Scale bars mark 30 and 10 μm in (B) and (C) and in (D) and (E), respectively. See also Figure S1 and Table S1.

suppressed in wild-type embryos but unrestrained in certain mutants, and (4) how the magnitude of variability differs spatially among phase-linked cells across a tissue.

To address these questions, we counted RNA molecules transcribed by two master segmentation clock genes (*her1* and *her7*) using single-molecule fluorescence *in situ* hybridization (smFISH) (STAR Methods) in intact zebrafish embryos. We found low amplitudes and high noise of *her1* and *her7* transcription in wild-type embryos. Cell-to-cell variability of clock gene expression is dominated by gene extrinsic noise. In Notch signaling mutants, amplitudes of oscillations decreased, while variability increased. Strikingly, transcriptional noise increased spatially from the posterior progenitor zone toward the anterior segmentation zone in both wild-type and Notch signaling mutant embryos. By computational modeling, we showed that the spatially increasing profile of gene expression noise can be recapitulated by the spatially increasing gene expression time delays in the clock network.

RESULTS

Segmentation Clock Oscillations Have Low Amplitudes

We combined two strategies in our approach. First, we grouped cells based on their oscillation phases. The oscillation period of segmentation clock genes increases smoothly along the posterior-to-anterior (tail-to-head) direction of the presomitic mesoderm (PSM) but remains constant along the left-to-right and dorsal-to-ventral axes (Giudicelli et al., 2007; Gomez et al., 2008). The slowdown of oscillations along the posterior-anterior axis causes a phase delay in cells located in the anterior PSM compared to those located in the posterior PSM. As a result, one sees the different phases of the oscillator cycle mapped out in space along the length of the PSM. Hence, two to three kinematic waves of gene expression can be detected at any moment of an oscillation cycle (Figures 1A–1C). In other words, all cells located at the same posterior-anterior position in a 2-dimensional, single-cell-wide cross-section are in the same phase of oscillations (Mara et al., 2007; Ozbudak and Lewis, 2008; Riedel-Kruse et al., 2007). Exploiting this unique spatial-to-temporal correlation of the segmentation clock, we collected precise, quantifiable data describing the system in detail (Figure 1). Second, we quantified mRNA numbers in single cells in an intact tissue. We used smFISH to quantify the number of

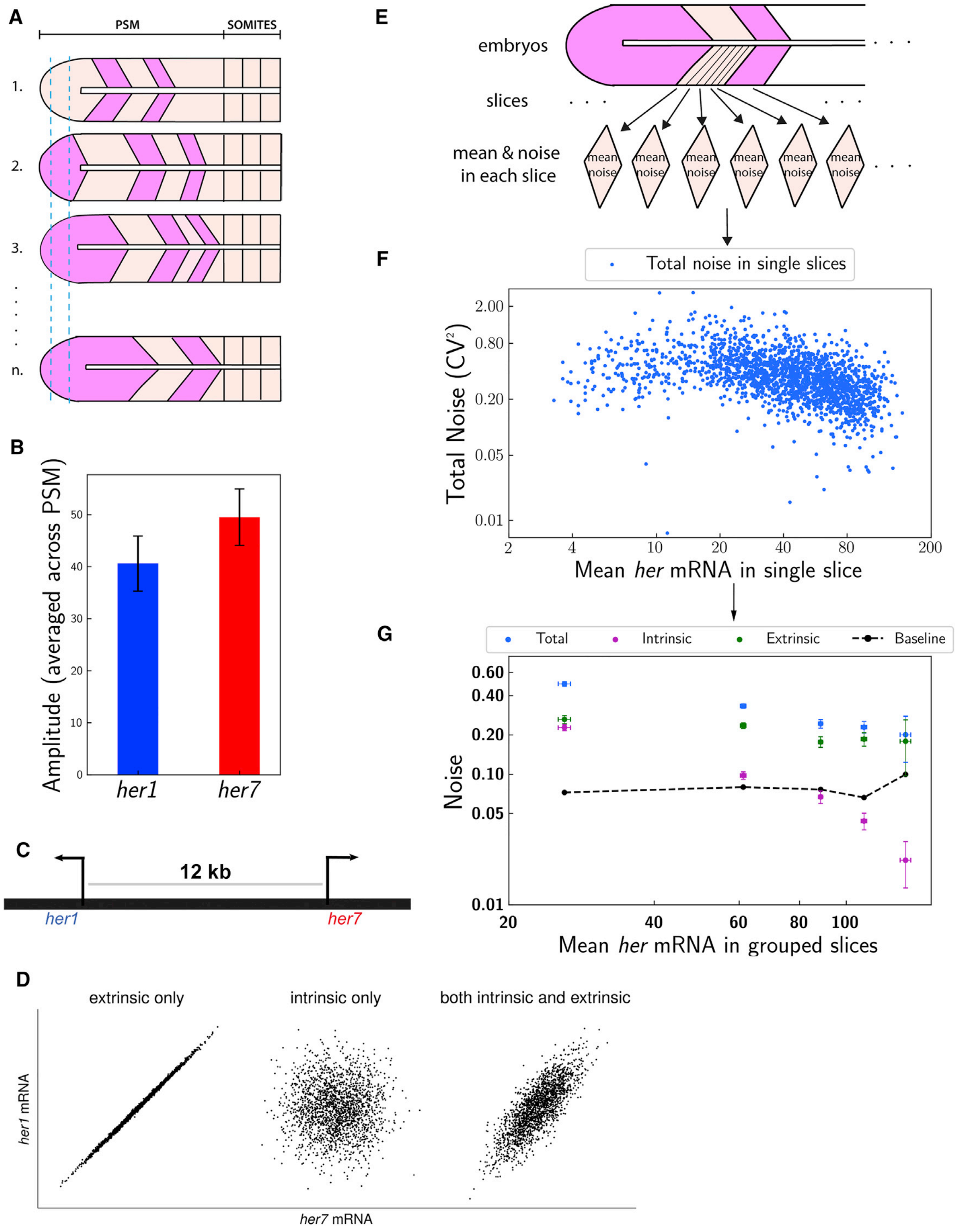

Figure 2. Extrinsic Noise Dominates Cell-to-Cell Expression Variability

(A) Amplitudes of oscillations are measured comparing peak (pink) and through (cream) mRNA levels in cells corresponding to same position (such as marked between the vertical blue dashed lines) in the PSM of different embryos.

(B) *her1* and *her7* have similar RNA amplitudes (averaged over all positions in the PSM).

(C) Duplicated paralogous *her1* and *her7* genes are located in chromosome 5 adjacent to each other; distance between *her1* and *her7* gene loci is about 12 kb.

(D) The expected profiles of her1 and *her7* RNA numbers if the variability is dominated by gene extrinsic sources (left panel), intrinsic sources (middle panel), or both.

(E) The PSM is divided into single-cell-diameter spatial slices, grouping cells in the same clock phases. Mean expression and noise values are calculated for each slice in each embryo.

(legend continued on next page)

RNA molecules in phase-grouped cells along the posterior-anterior axis (Gross-Thebing et al., 2014; Wang et al., 2012). Single cells in the intact tissue were distinguished by jointly analyzing nuclear (DAPI) and cell membrane (membrane-localized GFP) markers (Figures 1B and S1). Serial sections of fluorescent images of flat-mounted embryos were captured by using a 63× (numerical aperture [NA] = 1.4) objective at 0.240 μm intervals for up to 30 μm (STAR Methods). Thereby, each RNA molecule was detected as a diffraction-limited bright fluorescent dot in the cell (Figures 1C–1G). The number of clock RNAs per cell did not systematically depend on the z axis, suggesting uniform RNA detection efficiency along the tissue depth (Figure S1F). To calculate background staining, we used segmented somites, in which expression of clock genes switches off, as a negative control. The background staining was very low (3 and 1 dots per cell for *her1* and *her7*, respectively). To assess the efficiency of RNA detection, we compared the total number of *her1* RNA molecules in the PSM of wild-type embryos detected by smFISH to that detected by qRT-PCR (ratio = 1.08 ± 0.37) (STAR Methods). These results validated that a single fluorescent dot in smFISH images corresponds to a single RNA molecule.

We carried out a two-color smFISH experiment to count the number of *her1* and *her7* RNAs simultaneously in 10- to 14-somite-staged wild-type embryos. In total, we used 18 wild-type embryos. Each embryo captured a particular snapshot of the kinematic waves of the segmentation clock and hence displayed on-off stripes at different positions. The amplitude of oscillations was calculated by subtracting the RNA counts at each spatial position of embryos with low-expressing cells from that of embryos with high-expressing cells (Figure S2A) and taking the average across spatial positions (Figure 2A). Although prior computational modeling studies assumed larger amplitude oscillations in the segmentation clock (Ay et al., 2013, 2014), our data showed that *her1* and *her7* RNAs have average amplitudes of 41 ± 9 and 49 ± 9, respectively (Figure 2B). The number of clock transcripts is an order of magnitude lower than those of developmental genes controlling patterning and morphogenesis in *Drosophila* and housekeeping genes in mammalian cells (Boettiger and Levine, 2013; Little et al., 2013; Padovan-Merhar et al., 2015). It is remarkable that embryos can robustly accomplish segmentation by transcribing two master oscillating genes at such low levels, where they would be more vulnerable to gene expression noise (Balázsi et al., 2011; Elowitz et al., 2002; Ozbudak et al., 2002). Next, we investigated the magnitude of expression noise in the clock genes.

Gene Extrinsic Noise Dominates the Total Cell-to-Cell Expression Variability

The two master segmentation clock genes (*her1* and *her7*) are adjacent but separated by a common regulatory sequence and transcribed in opposite directions (Figure 2C). These genes are paralogous duplicates and code for transcriptional repressors that function as hetero- or homo-dimers. Expression of clock genes is repressed by their protein products, which create a negative feedback loop that is thought to be the pacemaker of the segmentation clock (Harima et al., 2013; Lewis, 2003). *her1* and *her7* have similar transcriptional time delays (Hanisch et al., 2013) and RNA half-lives (Giudicelli et al., 2007). Transcription of *her1* and *her7* is therefore concomitant (Gajewski et al., 2003; Oates and Ho, 2002), and our preceding results showed that they have comparable amplitudes (Figure 2B). Intrinsic fluctuations are those due to the randomness inherent to transcription; being random, they should affect the transcription of *her1* and *her7* independently, producing uncorrelated variations in respective mRNA levels (Figure 2D) (Elowitz et al., 2002). Other molecular species in the cell, e.g., RNA polymerases and upstream transcriptional regulators, are gene products and therefore will also vary over time and from cell to cell. These variations cause correlated fluctuations in the expression of clock genes and are defined as extrinsic noise (Elowitz et al., 2002). To separate the contributions of intrinsic and extrinsic sources in the expression variability of these two clock genes, we took an approach similar to earlier works (Figure S3; Supplemental Experimental Procedures) (Elowitz et al., 2002; Rhee et al., 2014).

We first calculated expression variability among phase-grouped cells (single-cell-diameter spatial slices) (Figures 2E and 2F) and then further grouped the slices into five bins based on mean clock mRNA levels (Figure 2G). Our results showed that the total expression variability of segmentation clock genes, as described by CV^2 ($[SD/mean]^2$), is 0.49 (i.e., CV = 0.7) at the lowest transcription state and 0.20 (i.e., CV = 0.45) at the highest transcription state (Figure 2G), which is substantially higher than the measurement error (Figure 2G; Supplemental Experimental Procedures). The total clock gene expression noise (Figure 2G) is as high as the levels observed in single-cell systems (Golding et al., 2005; Raj et al., 2006; Taniguchi et al., 2010) but much higher than those of developmental genes in *Drosophila* (Boettiger and Levine, 2013; Little et al., 2013), likely due to lower transcript levels (Figure 2B). The gene extrinsic noise contributes most to total variability in transcription ($p < 0.001$), while the gene intrinsic noise is only high at low transcript levels and decreases proportionally to the total clock transcript level (Figure 2G). Slight differences in the promoter strengths of *her1*/*her7* genes and transcriptional time delays (Hanisch et al., 2013) and half-lives of *her1*/*her7* mRNAs (Giudicelli et al., 2007) might have slightly increased gene intrinsic noise in our calculations. However, our conclusion that extrinsic noise dominates clock transcriptional variability holds true. In yeast, noise in gene expression is primarily extrinsic in origin (Becskei et al.,

(F) Total noise (CV^2 = $[SD/mean]^2$) versus mean levels of total *her* (*her1* + *her7*) RNA is plotted. Each blue dot represents the values in a single slice. All slice data from all wild-type embryos are pooled.
(G) Total noise split into intrinsic and extrinsic components for each slice and all slice data reported in (F) grouped into five bins based on mean *her* RNA levels. The intrinsic noise decreases with the average RNA levels (magenta). Extrinsic noise (green) is the dominant component of total expression noise (blue), $p < 0.001$ (low and high expression). Total measurement error due to RNA counting, cell segmentation, and phase grouping of cells is plotted as a baseline (dashed black curve). y axis is noise; x axis is mean levels of total *her* (*her1* + *her7*) RNA in grouped slices. The graph is in log-log scale; error bars indicate 2 SEs.
See also Figures S2 and S3.

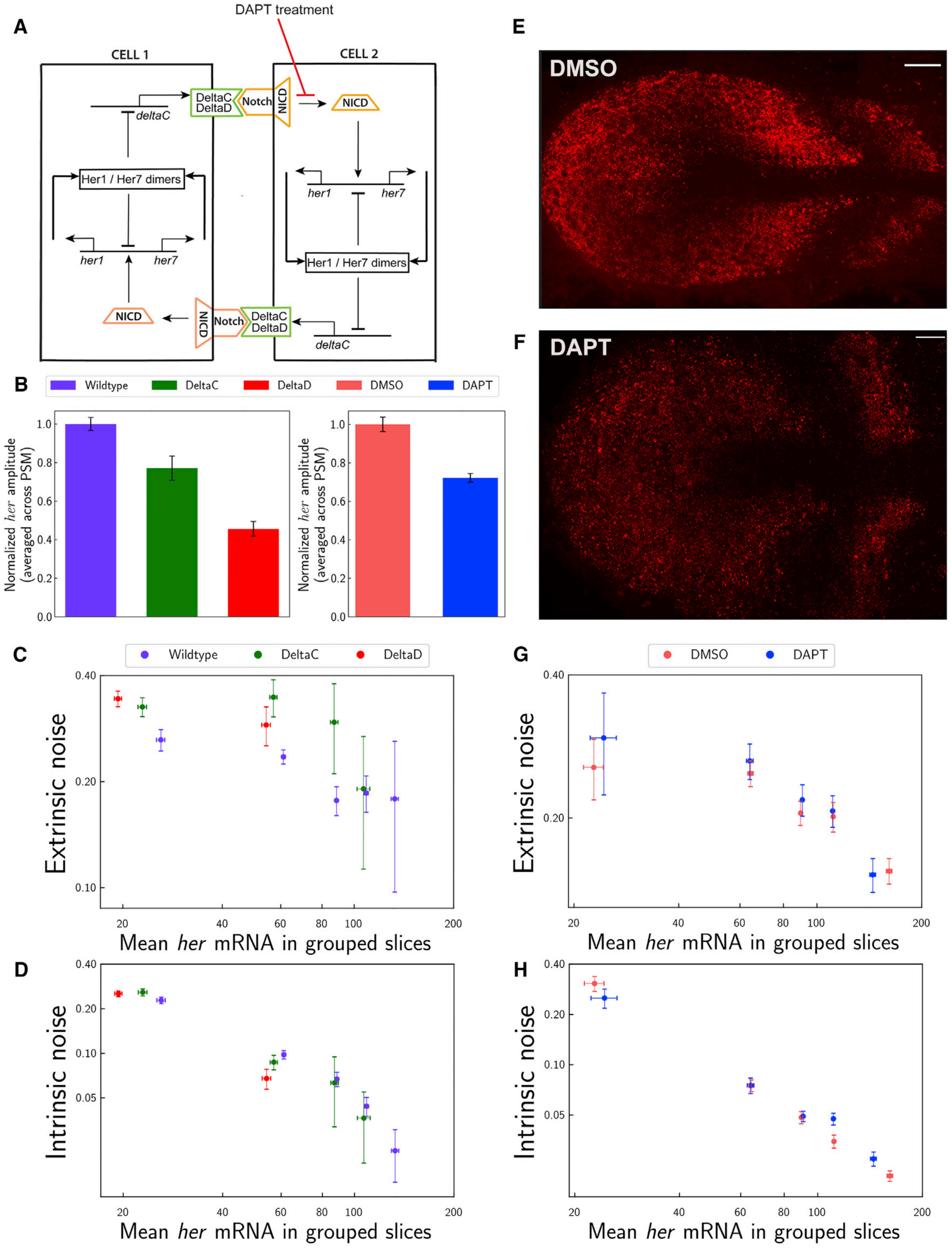

Figure 3. Notch Signaling Tames Extrinsic Noise

(A) Cartoon model for Notch-mediated cell-to-cell coupling. Her dimers repress transcription of her-family genes and *deltaC*. DeltaC (and DeltaD) are membrane-bound ligands (green) that bind and activate Notch (orange) receptor in neighboring cells. Intracellular cleaved domain of Notch (NICD) activates transcriptions of her-family genes.

(B) Total RNA amplitudes decreased in *deltaC*$^{-/-}$ (green) and *deltaD*$^{-/-}$ (red) mutants compared to wild-type embryos (purple) and in DAPT-treated embryos (blue) compared to DMSO-treated control embryos (pink) ($p < 0.001$).

(legend continued on next page)

OPEN ACCESS
CellPress

2005; Colman-Lerner et al., 2005; Raser and O'Shea, 2004; Volfson et al., 2006); however, the intrinsic noise is found to dominate for *hb* patterning gene in *Drosophila* (Little et al., 2013). Cell cycle is less likely to contribute to extrinsic noise, because the segmentation clock period is much smaller than cell cycle and only a small population of cells experience mitosis during an oscillation cycle in the zebrafish PSM (Horikawa et al., 2006). We next assessed whether variation in cell size could underlie the dominant extrinsic noise. We reanalyzed our data by normalizing transcript counts by cell volume. The results showed that cell volume has only a minor contribution on the expression noise and extrinsic noise is the dominant portion of clock variability (Figure S2B). Because *her1* and *her7* genes are located adjacent to each other, common upstream factors (Becskei et al., 2005; Volfson et al., 2006) or local chromatin modifications are likely the main factors resulting in dominant extrinsic noise, as shown in yeast (Becskei et al., 2005), but one cannot exclude variations in epigenetic and metabolic states.

Notch Signaling Buffers Mostly Gene Extrinsic Noise

After finding that extrinsic noise dominates the gene expression variability in the segmentation clock, we investigated the mechanism that might limit its magnitude. In *Drosophila*, noise in nascent transcription of *hb* gene is as high as that of the zebrafish segmentation clock genes, but noise in *Drosophila* patterning genes is averaged out by spatial averaging in syncytial embryo and by temporal averaging due to longer half-life of RNA (Little et al., 2013). However, spatial averaging is not possible in the vertebrate PSM cells, because they are not syncytial, and the short half-lives of RNAs (3–8 min) (Giudicelli et al., 2007) prevent simple temporal averaging.

Oscillatory membrane-bound DeltaC ligands activate Notch receptors in neighboring cells; hence, Notch signaling acts at a short distance (Figure 3A). The activated intracellular domain of the Notch receptor, in turn, activates transcription of *her*-family genes. This short-distance coupling synchronizes oscillation phases among cells located in the same anterior-posterior position in the PSM (Delaune et al., 2012; Horikawa et al., 2006; Jiang et al., 2000; Mara et al., 2007; Ozbudak and Lewis, 2008; Riedel-Kruse et al., 2007). Based on experimental observations and computational modeling, Notch signaling has been hypothesized to minimize gene expression noise (Delaune et al., 2012; Horikawa et al., 2006; Jiang et al., 2000; Mara et al., 2007; Ozbudak and Lewis, 2008; Riedel-Kruse et al., 2007), but gene expression noise has not been quantified so far. To quantify noise, binary descriptions such as "synchronous versus asynchronous (salt-and-pepper)" are insufficient; one must count single molecules (Raj and van Oudenaarden, 2009) and quantify their distributions in cells that are grouped according to their oscillation phases. To investigate the impact of Notch signaling-mediated cell-to-cell coupling on expression noise, we carried out two-color smFISH experiments in two mutant lines—*deltaC*$^{-/-}$ (Jülich et al., 2005) and *deltaD*$^{-/-}$ (Holley et al., 2000)—in which Notch signaling is disrupted and stripy expression patterns are lost due to desynchronized oscillations. The amplitude of oscillations and mean RNA levels decreased (ranging from 23% to 54%) in two mutants compared to wild-type embryos ($p < 0.006$) (Figures 3B and S3). Because transcriptional noise depends on mean RNA levels (Figure 2G), we compared noise between mutants and wild-type embryos at similar mean RNA levels. The mutants lacked higher expression groups due to reduced clock transcription. Total expression noise increased (more than 30%) in two mutants compared to wild-type embryos due to extrinsic ($p < 0.001$), but not intrinsic, noise (Figures 3C and 3D). Extrinsic noise was elevated predominantly in the lower expression states. However, the mutant data reflect the steady-state response and might miss the initial outcome of loss of Notch signaling. To investigate this issue further, we transiently blocked Notch signaling by treating embryos with the γ-secretase inhibitor *N*-[N-(3,5-difluorophenacetyl)-L-alanyl]-S-phenylgly cine t-butyl ester (DAPT) or DMSO (as a control) for 1.5 hr. Earlier work showed that long-term DAPT treatment recapitulated segmentation defects in mutants (Mara et al., 2007; Ozbudak and Lewis, 2008; Riedel-Kruse et al., 2007). Two-color smFISH experiments showed that blocking of Notch signaling for only 1.5 hr did not disrupt the stripe expression patterns (Figures 3E and 3F) as reported previously, suggesting that the global phase synchrony is not disrupted with short-term DAPT treatment (Mara et al., 2007; Ozbudak and Lewis, 2008; Riedel-Kruse et al., 2007). However, the amplitude of oscillations decreased (28%, Figures 3B and S3) while the expression noise slightly increased in the DAPT-treated embryos compared to DMSO-treated embryos. As expected, the elevation of noise in DAPT- versus DMSO-treated embryos (Figures 3G and 3H) is much lower than that in mutants versus wild-type embryos (Figures 3C and 3D) due to the transient nature of drug treatment. Increased noise would then accumulate in time to cause both amplitude variability and phase drifting among neighboring cells and result in higher variability in mutant (*deltaC*$^{-/-}$ and *deltaD*$^{-/-}$) embryos. These results affirmed the long-standing hypothesis that the cause of segmentation defects in Notch signaling mutants is untamed gene expression noise in the segmentation clock genes (Jiang et al., 2000) and discovered that the main function of Notch signaling is to dampen extrinsic noise.

Although Notch signaling is an activator of clock transcription (Figure 3B), total noise did not primarily increase due to the decreased clock transcription in Notch mutants for the following

(C and D) Extrinsic noise (C) increased in *deltaC*$^{-/-}$ (green) and *deltaD*$^{-/-}$ (red) mutants compared to wild-type embryos (purple) ($p < 0.001$ in both lower and higher groups). Intrinsic noise levels (D) are shown in *deltaC*$^{-/-}$ (green) and *deltaD*$^{-/-}$ (red) mutants and wild-type embryos (purple). x axis is mean levels of total *her* (*her1* + *her7*) RNA in grouped slices. Clock transcription decreased in notch mutants. Therefore, slices could only be grouped for the lower expression bins compared to wild-type data.
(E and F) A single z-section of a smFISH picture for *her7* mRNA (red) in DMSO-treated embryos (as a control, E) and DAPT-treated embryos (F). The stripy expression pattern of *her7* mRNA is not disrupted after 1.5 hr DAPT treatment (F). Scale bars mark 30 μm.
(G and H) Extrinsic (G) and intrinsic (H) noise levels in DAPT-treated embryos (blue) and DMSO-treated control embryos (pink).
Error bars indicate 2 SEs. See also Figure S3.

reasons. First, gene intrinsic noise displays an inverse relationship with mean expression levels (Elowitz et al., 2002; Kaern et al., 2005) (Figure 2G). However, total noise increased in mutants due to increased extrinsic, but not intrinsic, noise (Figures 3C and 3D). Second, the amplitude of oscillations reduced twice in *deltaD*$^{-/-}$ mutant compared to *deltaC*$^{-/-}$ mutant, but intrinsic noise was not higher in *deltaD*$^{-/-}$ mutant than in *deltaC*$^{-/-}$ mutant. Altogether, these results show that total noise is elevated in Notch pathway mutants due to both desynchronized oscillations and decreased amplitudes, and surprisingly, these two factors increase only extrinsic, not intrinsic, noise. Extrinsic noise is the dominant component of the transcriptional variability in wild-type embryos (Figure 2G), which implies that transcriptions of *her1* and *her7* genes fire nearly simultaneously in each PSM cell. When Notch signaling is disrupted, extrinsic noise is elevated further (Figures 3C and 3D). This result shows that Notch signaling is not the source of co-transcription of two clock genes but rather actively tames gene extrinsic noise. We hypothesize that high extrinsic noise is likely due to the cell-to-cell variability in either the levels of other transcriptional regulators or the epigenetic state of the chromosomal locus.

Gene Expression Noise Increases from the Progenitor to the Segmentation and Differentiation Zone

Afterward, we assessed how noise in clock gene expression varies in space from the posterior PSM (progenitor zone) to the anterior PSM (where cells initiate segmentation) by pooling the noise values at each spatial position from all wild-type embryos (Figure 4A) (see STAR Methods for details). Strikingly, we found that expression noise increases toward the anterior PSM in wild-type embryos ($p < 0.001$) (Figure 4B). In Notch signaling mutants (*deltaC*$^{-/-}$ and *deltaD*$^{-/-}$), the spatial profile of gene expression noise does not flatten but rather elevated throughout the PSM ($p < 0.001$) (Figure 4B). Because total noise (CV^2) depends on average expression levels (Figure 2G), we assessed the spatial change of noise by grouping data based on mean expression levels (see STAR Methods for details). Transcriptional noise is higher in cells located in anterior PSM than those in posterior PSM at comparable mean RNA levels ($p < 0.001$) (Figures 4C, 4D, and S4; STAR Methods). The results showed that the total noise is minimized by Notch signaling throughout the PSM in wild-type embryos but is boosted in Notch signaling mutants, and Notch signaling does not play a role in the anteriorly increasing trend of noise in wild-type embryos (Figure 4).

These results could be perceived as unexpected, because cell-to-cell variability should be minimized for cohorts of cells to segment robustly at the end of anterior PSM. This paradox can be explained by our earlier results, in which we have shown that the segmentation clock functions primarily in cells located in the posterior PSM. The segmentation clock relays its periodic information to downstream genes in the middle of the PSM (Giudicelli et al., 2007); thereafter, the clock is not needed for the segmentation process to be completed, although it continues to be expressed in the anterior PSM. It has been puzzling why expression of the segmentation clock became functionally irrelevant for cells located in the anterior PSM. Our results demonstrating increased gene expression variability of clock genes in anterior PSM reveal why cells could not rely on noisy clock expression in the anterior PSM. Collectively, our results show that there is selection pressure to minimize noise in clock gene expression only in the posterior PSM (Figure 4B). These results are the first demonstration of spatial variation of gene expression noise during vertebrate development.

We further investigated the mechanism driving spatial gradient of clock genes' expression noise. A candidate mechanism is the spatially increasing effective gene expression time delays along the posterior-anterior direction (Ay et al., 2014). We built a simple stochastic computational model to test the impact of time-delay gradients on the expression noise in the tissue (Figures 4E and S5; Supplemental Experimental Procedures). In our model, we simulated synchronized oscillations in two neighboring cells. Cell-autonomous oscillations are generated due to an intracellular negative feedback loop (with an effective time delay of τ_x), and the clocks are synchronized due to an intercellular positive feedback loop (with an effective time delay of τ_y). We then repeated simulations by increasing the time delays gradually up to 4.5-fold—a value that matches to the experimentally quantified increase in effective time delays along the PSM (Ay et al., 2014). The simulations recapitulated the same spatially increasing trend in gene expression noise as time delays are increased along the tissue (Figure 4F).

DISCUSSION

Many studies of stochastic gene expression have been performed with synthetic regulatory networks or promoter reporters for natural networks by using long-lived *GFP* mRNA and protein. However, many mRNAs coding for critical transcription factors governing embryonic development and adult homeostasis are short lived (Schwanhäusser et al., 2011). Here, by studying short-lived segmentation clock RNAs ($t_{1/2}$ = 3–5 min) (Giudicelli et al., 2007), we investigated gene expression noise for the first time at the fastest dynamic scale (30-min period) in an intact vertebrate tissue.

Earlier work evaluated her/hes-family gene expression in embryos by simple binary scoring (signal present versus absent) (Delaune et al., 2012; Horikawa et al., 2006; Jenkins et al., 2015; Jiang et al., 2000; Mara et al., 2007; Ozbudak and Lewis, 2008; Riedel-Kruse et al., 2007), by arbitrary fluorescence units instead of absolute molecular counting (Webb et al., 2016), or in cell culture in which spatial information and Notch coupling were lost (Phillips et al., 2016; Webb et al., 2016). Here we used single-molecule counting in an intact vertebrate tissue to analyze the amplitude and variability of the segmentation clock genes in different genetic backgrounds and spatial positions. Our results showed that the amplitudes of transcriptional oscillations during somitogenesis are very low compared to RNA levels of developmental genes in other systems (Boettiger and Levine, 2013; Little et al., 2013) and extrinsic noise contributes the most to the high variability of clock gene expression (Figure 2). The period of oscillations is short (~30 min), and transcriptional time delays make up a significant portion of the period (Giudicelli et al., 2007). This would restrict the number of proteins that can be translated from a transcript in a given clock cycle. Unlike constitutively expressed and stable proteins, the segmentation clock proteins are short lived (Ay et al., 2013). Therefore, clock protein levels

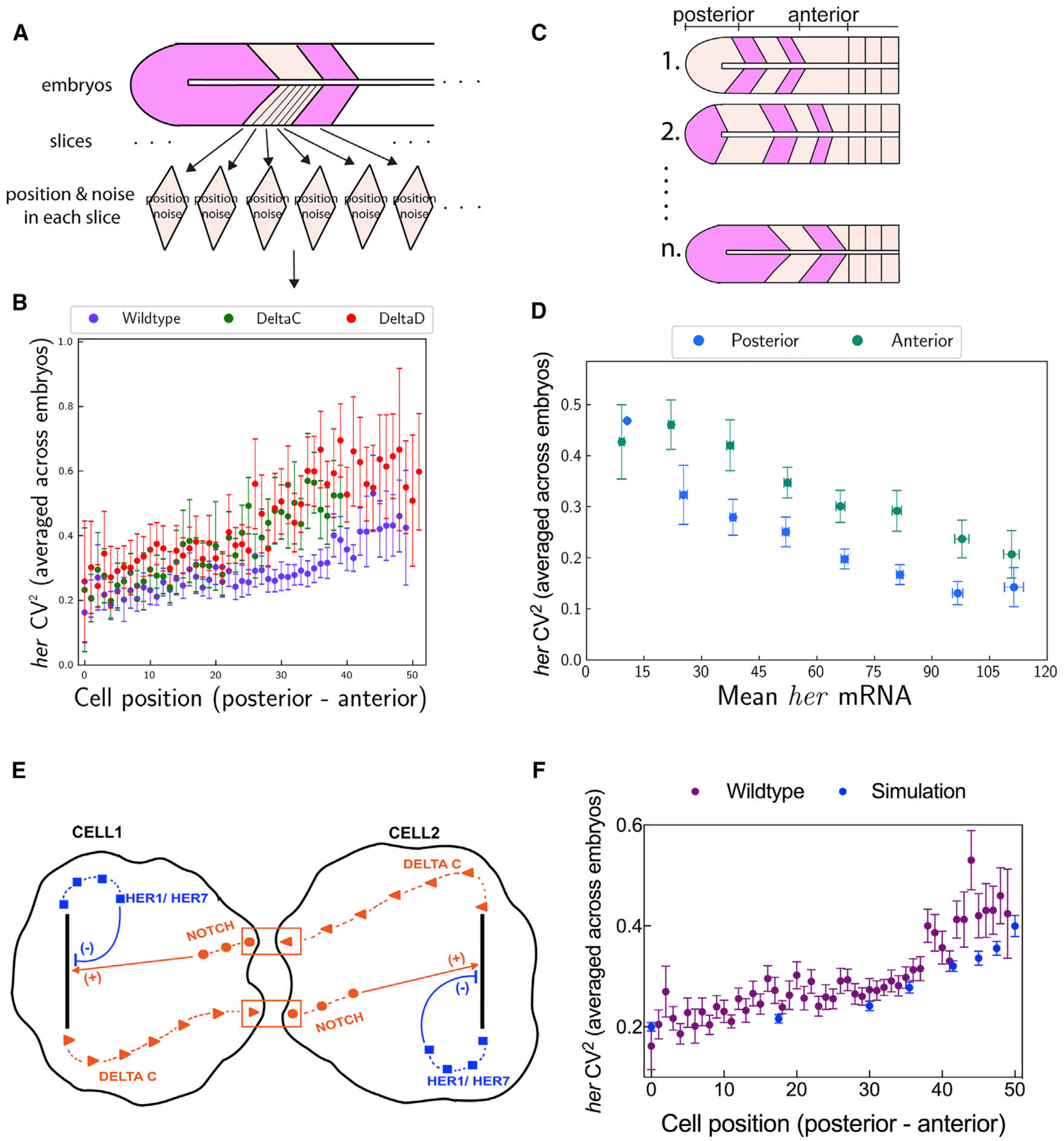

Figure 4. Expression Noise Displays an Increasing Profile along the PSM

(A) Expression noise is analyzed in the PSM.

(B) The total *her* expression noise increased in *deltaC*$^{-/-}$ (green) and *deltaD*$^{-/-}$ (red) mutants compared to that in wild-type (purple) throughout the PSM, $p < 0.001$ (wild-type and *deltaC*$^{-/-}$ in posterior and anterior), $p < 0.001$ (wild-type and *deltaD*$^{-/-}$ in posterior and anterior). x axis is spatial positions.

(C) Representative sketches of n embryos at different oscillation snapshots are shown. Noise values from posterior and anterior PSM of all embryos are grouped in the first and second groups, respectively.

(D) Expression noise is higher in cells located in the posterior versus the anterior PSM, $p < 0.001$. Expression noise (y axis) decreases as expression levels (x axis) increases, as shown in Figure 2G. However, at a given expression level, cells located in the anterior PSM have higher expression noise for the clock genes than cells located in the posterior PSM. The spatially increasing trend of expression noise (B) is independent of mean expression levels.

(E) A cartoon figure shows intracellular and intercellular time delays.

(F) The simulation result (blue) recapitulates increased total expression noise throughout the PSM (posterior to anterior) in wild-type embryos (purple).

In (A)–(D) and (F), posterior is to the left and anterior is to the right. Error bars indicate 2 SEs. See also Figures S4 and S5.

closely follow mRNA levels. Furthermore, clock RNAs and proteins establish a transcriptional negative feedback loop. All these features make clock mRNA levels the best reporters of clock protein levels. Here, we hypothesize that a major portion of transcriptional extrinsic noise is due to variability in clock protein levels. Although clock proteins have not been counted yet, published data revealed extensive cell-to-cell variability in Her1-Venus reporter levels (Delaune et al., 2012). In this study, we

have investigated the precision in the amplitude of oscillations. Investigating the precision in the timing of oscillations (Morelli and Jülicher, 2007) would require carrying out single-molecule RNA or protein counting in real time. The levels of Her1/Her7 proteins should be above a threshold to repress many target genes, including *deltaC*; precision in their levels is therefore important for the successful segmentation of somites.

The timely and precise progression of a genetic program is critical to achieving reproducible embryonic development. Unlike other systems (Little et al., 2013; Shah and Tyagi, 2013), in somitogenesis, gene expression variability is not averaged in time or space. Here, we showed that vertebrate embryos use Notch signaling-mediated cell-to-cell coupling to minimize expression variability throughout the PSM (Figure 3). In the absence of Notch signaling, increased gene expression noise (Figure 3) desynchronizes clock oscillations and results in segmentation defects (Mara et al., 2007; Ozbudak and Lewis, 2008; Riedel-Kruse et al., 2007) and congenital scoliosis in patients (Pourquié, 2011). Lastly, we found that transcription noise increases spatially as cells approach the segmentation zone, likely due to spatially increasing gene expression time delays but independent of Notch signaling (Figure 4). Because time delays are inevitable in the expression of every gene, understanding the role of time delays in expression noise is significant. We showed that the spatial gradient of effective time delays in the PSM (Ay et al., 2014) likely contributes to the spatially increasing expression noise of the segmentation clock genes along the axis (Figure 4F).

Oscillations are widespread in biological systems. Notch signaling plays critical roles in controlling the switch from proliferation to differentiation in almost every tissue throughout the metazoan. *Hes/her* family genes are direct targets of Notch signaling. The segmentation clock was the first discovered developmental oscillator (Palmeirim et al., 1997). However, Hes/Her protein levels also oscillate in neural progenitors, embryonic stem cells, and ovarian cells, thereby controlling the switch from proliferation to differentiation in several tissues (Kobayashi and Kageyama, 2014). Furthermore, Hes/Her proteins are highly expressed in several types of human tumors, while their inhibition has been shown to restore differentiation (Kobayashi and Kageyama, 2014; Liu et al., 2015; Sang et al., 2010). We anticipate that future studies may lead to developing new ways to control stem cell proliferation and differentiation in various tissues, developing therapies against certain cancer types, understanding the precision of other natural oscillators, and engineering synthetic oscillators.

STAR★METHODS

Detailed methods are provided in the online version of this paper and include the following:

- KEY RESOURCES TABLE
- CONTACT FOR REAGENT AND RESOURCE SHARING
- EXPERIMENTAL MODEL AND SUBJECT DETAILS
  - Fish stocks
- METHOD DETAILS
  - smFISH and imaging
  - Quantifying RNA numbers in single cells
  - qRT-PCR
  - Calculating the efficiency of RNA detection by smFISH
  - Calculating the measurement error in RNA counting by smFISH
  - Calculating the measurement error in cell segmentation
  - Grouping cells in space based on oscillation phases
  - Calculating the measurement error in phase grouping of cells
  - Calculating the total measurement error
- QUANTIFICATION AND STATISTICAL ANALYSIS
  - Heatmaps

SUPPLEMENTAL INFORMATION

Supplemental Information includes Supplemental Experimental Procedures, five figures, one table, and two data files and can be found with this article online at https://doi.org/10.1016/j.celrep.2018.04.069.

ACKNOWLEDGMENTS

We thank Stephan Knierer and Dong Mai for technical assistance. We thank Claude Desplan for critically reading the manuscript. This work was funded by NIH grants (GM111987 and GM122956) to E.M.Ö. and a Colgate Picker Interdisciplinary Science Institute grant to E.M.Ö. and A.A.

AUTHOR CONTRIBUTIONS

S.K. and G.S.D. performed the experiments. S.B.K., H.T.V., Q.H., Y.Y.L., A.A., and E.M.Ö. analyzed the data. M.S. and A.S. contributed the computational model. E.M.Ö. designed and supervised the project and wrote the manuscript.

DECLARATION OF INTERESTS

The authors declare no competing interests.

Received: October 19, 2017
Revised: March 5, 2018
Accepted: April 16, 2018
Published: May 15, 2018

SUPPORTING CITATIONS

The following references appear in the Supplemental Information: Gillespie (1976).

REFERENCES

Ay, A., Knierer, S., Sperlea, A., Holland, J., and Özbudak, E.M. (2013). Short-lived Her proteins drive robust synchronized oscillations in the zebrafish segmentation clock. Development *140*, 3244–3253.

Ay, A., Holland, J., Sperlea, A., Devakanmalai, G.S., Knierer, S., Sangervasi, S., Stevenson, A., and Ozbudak, E.M. (2014). Spatial gradients of protein-level time delays set the pace of the traveling segmentation clock waves. Development *141*, 4158–4167.

Balázsi, G., van Oudenaarden, A., and Collins, J.J. (2011). Cellular decision making and biological noise: from microbes to mammals. Cell *144*, 910–925.

Becskei, A., Kaufmann, B.B., and van Oudenaarden, A. (2005). Contributions of low molecule number and chromosomal positioning to stochastic gene expression. Nat. Genet. *37*, 937–944.

Boettiger, A.N., and Levine, M. (2013). Rapid transcription fosters coordinate snail expression in the *Drosophila* embryo. Cell Rep. *3*, 8–15.

Colman-Lerner, A., Gordon, A., Serra, E., Chin, T., Resnekov, O., Endy, D., Pesce, C.G., and Brent, R. (2005). Regulated cell-to-cell variation in a cell-fate decision system. Nature *437*, 699–706.

Cooper, M.S., Szeto, D.P., Sommers-Herivel, G., Topczewski, J., Solnica-Krezel, L., Kang, H.C., Johnson, I., and Kimelman, D. (2005). Visualizing morphogenesis in transgenic zebrafish embryos using BODIPY TR methyl ester dye as a vital counterstain for GFP. Dev. Dyn. *232*, 359–368.

Delaune, E.A., François, P., Shih, N.P., and Amacher, S.L. (2012). Single-cell-resolution imaging of the impact of Notch signaling and mitosis on segmentation clock dynamics. Dev. Cell *23*, 995–1005.

Elowitz, M.B., Levine, A.J., Siggia, E.D., and Swain, P.S. (2002). Stochastic gene expression in a single cell. Science *297*, 1183–1186.

Gajewski, M., Sieger, D., Alt, B., Leve, C., Hans, S., Wolff, C., Rohr, K.B., and Tautz, D. (2003). Anterior and posterior waves of cyclic her1 gene expression are differentially regulated in the presomitic mesoderm of zebrafish. Development *130*, 4269–4278.

Gillespie, D.T. (1976). A general method for numerically simulating the stochastic time evolution of coupled chemical reactions. J. Comp. Physiol. *22*, 403–434.

Giudicelli, F., Ozbudak, E.M., Wright, G.J., and Lewis, J. (2007). Setting the tempo in development: an investigation of the zebrafish somite clock mechanism. PLoS Biol. *5*, e150.

Golding, I., Paulsson, J., Zawilski, S.M., and Cox, E.C. (2005). Real-time kinetics of gene activity in individual bacteria. Cell *123*, 1025–1036.

Gomez, C., Ozbudak, E.M., Wunderlich, J., Baumann, D., Lewis, J., and Pourquié, O. (2008). Control of segment number in vertebrate embryos. Nature *454*, 335–339.

Gross-Thebing, T., Paksa, A., and Raz, E. (2014). Simultaneous high-resolution detection of multiple transcripts combined with localization of proteins in whole-mount embryos. BMC Biol. *12*, 55.

Hanisch, A., Holder, M.V., Choorapoikayil, S., Gajewski, M., Özbudak, E.M., and Lewis, J. (2013). The elongation rate of RNA polymerase II in zebrafish and its significance in the somite segmentation clock. Development *140*, 444–453.

Harima, Y., Takashima, Y., Ueda, Y., Ohtsuka, T., and Kageyama, R. (2013). Accelerating the tempo of the segmentation clock by reducing the number of introns in the Hes7 gene. Cell Rep. *3*, 1–7.

Holley, S.A., Geisler, R., and Nüsslein-Volhard, C. (2000). Control of her1 expression during zebrafish somitogenesis by a delta-dependent oscillator and an independent wave-front activity. Genes Dev. *14*, 1678–1690.

Horikawa, K., Ishimatsu, K., Yoshimoto, E., Kondo, S., and Takeda, H. (2006). Noise-resistant and synchronized oscillation of the segmentation clock. Nature *441*, 719–723.

Jenkins, R.P., Hanisch, A., Soza-Ried, C., Sahai, E., and Lewis, J. (2015). Stochastic Regulation of her1/7 Gene Expression Is the Source of Noise in the Zebrafish Somite Clock Counteracted by Notch Signalling. PLoS Comput. Biol. *11*, e1004459.

Ji, N., Middelkoop, T.C., Mentink, R.A., Betist, M.C., Tonegawa, S., Mooijman, D., Korswagen, H.C., and van Oudenaarden, A. (2013). Feedback control of gene expression variability in the *Caenorhabditis elegans* Wnt pathway. Cell *155*, 869–880.

Jiang, Y.J., Aerne, B.L., Smithers, L., Haddon, C., Ish-Horowicz, D., and Lewis, J. (2000). Notch signalling and the synchronization of the somite segmentation clock. Nature *408*, 475–479.

Jülich, D., Hwee Lim, C., Round, J., Nicolaije, C., Schroeder, J., Davies, A., Geisler, R., Lewis, J., Jiang, Y.J., and Holley, S.A.; Tübingen 2000 Screen Consortium (2005). beamter/deltaC and the role of Notch ligands in the zebrafish somite segmentation, hindbrain neurogenesis and hypochord differentiation. Dev. Biol. *286*, 391–404.

Kaern, M., Elston, T.C., Blake, W.J., and Collins, J.J. (2005). Stochasticity in gene expression: from theories to phenotypes. Nat. Rev. Genet. *6*, 451–464.

Kobayashi, T., and Kageyama, R. (2014). Expression dynamics and functions of Hes factors in development and diseases. Curr. Top. Dev. Biol. *110*, 263–283.

Lewis, J. (2003). Autoinhibition with transcriptional delay: a simple mechanism for the zebrafish somitogenesis oscillator. Curr. Biol. *13*, 1398–1408.

Little, S.C., Tikhonov, M., and Gregor, T. (2013). Precise developmental gene expression arises from globally stochastic transcriptional activity. Cell *154*, 789–800.

Liu, Z.H., Dai, X.M., and Du, B. (2015). Hes1: a key role in stemness, metastasis and multidrug resistance. Cancer Biol. Ther. *16*, 353–359.

Mara, A., Schroeder, J., Chalouni, C., and Holley, S.A. (2007). Priming, initiation and synchronization of the segmentation clock by deltaD and deltaC. Nat. Cell Biol. *9*, 523–530.

Morelli, L.G., and Jülicher, F. (2007). Precision of genetic oscillators and clocks. Phys. Rev. Lett. *98*, 228101.

Oates, A.C., and Ho, R.K. (2002). Hairy/E(spl)-related (Her) genes are central components of the segmentation oscillator and display redundancy with the Delta/Notch signaling pathway in the formation of anterior segmental boundaries in the zebrafish. Development *129*, 2929–2946.

Ozbudak, E.M., and Lewis, J. (2008). Notch signalling synchronizes the zebrafish segmentation clock but is not needed to create somite boundaries. PLoS Genet. *4*, e15.

Ozbudak, E.M., Thattai, M., Kurtser, I., Grossman, A.D., and van Oudenaarden, A. (2002). Regulation of noise in the expression of a single gene. Nat. Genet. *31*, 69–73.

Padovan-Merhar, O., Nair, G.P., Biaesch, A.G., Mayer, A., Scarfone, S., Foley, S.W., Wu, A.R., Churchman, L.S., Singh, A., and Raj, A. (2015). Single mammalian cells compensate for differences in cellular volume and DNA copy number through independent global transcriptional mechanisms. Mol. Cell *58*, 339–352.

Palmeirim, I., Henrique, D., Ish-Horowicz, D., and Pourquié, O. (1997). Avian hairy gene expression identifies a molecular clock linked to vertebrate segmentation and somitogenesis. Cell *91*, 639–648.

Phillips, N.E., Manning, C.S., Pettini, T., Biga, V., Marinopoulou, E., Stanley, P., Boyd, J., Bagnall, J., Paszek, P., Spiller, D.G., et al. (2016). Stochasticity in the miR-9/Hes1 oscillatory network can account for clonal heterogeneity in the timing of differentiation. eLife *5*, e16118.

Pourquié, O. (2011). Vertebrate segmentation: from cyclic gene networks to scoliosis. Cell *145*, 650–663.

Raj, A., and van Oudenaarden, A. (2009). Single-molecule approaches to stochastic gene expression. Annu. Rev. Biophys. *38*, 255–270.

Raj, A., Peskin, C.S., Tranchina, D., Vargas, D.Y., and Tyagi, S. (2006). Stochastic mRNA synthesis in mammalian cells. PLoS Biol. *4*, e309.

Raj, A., Rifkin, S.A., Andersen, E., and van Oudenaarden, A. (2010). Variability in gene expression underlies incomplete penetrance. Nature *463*, 913–918.

Raser, J.M., and O'Shea, E.K. (2004). Control of stochasticity in eukaryotic gene expression. Science *304*, 1811–1814.

Rhee, A., Cheong, R., and Levchenko, A. (2014). Noise decomposition of intracellular biochemical signaling networks using nonequivalent reporters. Proc. Natl. Acad. Sci. USA *111*, 17330–17335.

Riedel-Kruse, I.H., Müller, C., and Oates, A.C. (2007). Synchrony dynamics during initiation, failure, and rescue of the segmentation clock. Science *317*, 1911–1915.

Sanft, K.R., Wu, S., Roh, M., Fu, J., Lim, R.K., and Petzold, L.R. (2011). StochKit2: software for discrete stochastic simulation of biochemical systems with events. Bioinformatics *27*, 2457–2458.

Sang, L., Roberts, J.M., and Coller, H.A. (2010). Hijacking HES1: how tumors co-opt the anti-differentiation strategies of quiescent cells. Trends Mol. Med. *16*, 17–26.

Schwanhäusser, B., Busse, D., Li, N., Dittmar, G., Schuchhardt, J., Wolf, J., Chen, W., and Selbach, M. (2011). Global quantification of mammalian gene expression control. Nature *473*, 337–342.

Shah, K., and Tyagi, S. (2013). Barriers to transmission of transcriptional noise in a c-fos c-jun pathway. Mol. Syst. Biol. *9*, 687.

Taniguchi, Y., Choi, P.J., Li, G.W., Chen, H., Babu, M., Hearn, J., Emili, A., and Xie, X.S. (2010). Quantifying E. coli proteome and transcriptome with single-molecule sensitivity in single cells. Science *329*, 533–538.

Volfson, D., Marciniak, J., Blake, W.J., Ostroff, N., Tsimring, L.S., and Hasty, J. (2006). Origins of extrinsic variability in eukaryotic gene expression. Nature *439*, 861–864.

Wang, F., Flanagan, J., Su, N., Wang, L.C., Bui, S., Nielson, A., Wu, X., Vo, H.T., Ma, X.J., and Luo, Y. (2012). RNAscope: a novel *in situ* RNA analysis platform for formalin-fixed, paraffin-embedded tissues. J. Mol. Diagn. *14*, 22–29.

Webb, A.B., Lengyel, I.M., Jörg, D.J., Valentin, G., Jülicher, F., Morelli, L.G., and Oates, A.C. (2016). Persistence, period and precision of autonomous cellular oscillators from the zebrafish segmentation clock. eLife *5*, e08438.

STAR★METHODS

KEY RESOURCES TABLE

REAGENT or RESOURCE	SOURCE	IDENTIFIER
Antibodies		
Rabbit anti-GFP primary antibody	Life Technologies	Cat#A6455; RRID:AB_221570
Goat anti-Rabbit IgG Alexa 488 Secondary Antibody	Thermo Fisher Scientific	Cat#A-11034; RRID:AB_2576217
Chemicals, Peptides, and Recombinant Proteins		
RNAscope Fluorescent Multiplex Detection Reagents	Advanced Cell Diagnostics	Cat#320851
Hoechst trihydrochloride, trihydrate	Invitrogen	Cat#33342
ProLong Gold antifade reagent	Life Technologies	Cat#P36934
SP6 mMessage mMachine	Life Technologies	Cat#AM1340
Research Quick RNA MiniPrep Kit	Zymo	Cat#R1054
SuperScript IV Reverse Transcriptase	Invitrogen	Cat#18090010
RNaseOUT	Invitrogen	Cat#10777-019
dNTP	Roche	Cat#05081955001
SYBR Select Master Mix	Life Technologies	Cat#4472908
N-[N-(3,5-Difluorophenacetyl-L-alanyl)]-S-phenylglycine t-Butyl Ester	Calbiochem	Cat#565770
RNAscope Probe- Dr-her1-LE1	Advanced Cell Diagnostics	Cat No. 433191
RNAscope Probe- Dr-her1-LE2-C3	Advanced Cell Diagnostics	Cat No. 433201-C3
RNAscope Probe- Dr-her7	Advanced Cell Diagnostics	Cat No. 428611
Experimental Models: Organisms/Strains		
Zebrafish: Tg(*Ola.Actb:Hsa.HRAS-EGFP*)	Cooper et al., 2005	ZFIN ID: ZDB-ALT-061107-2
Zebrafish: $dlc^{tw212b/tw212b}$	Jülich et al., 2005	ZFIN ID: ZDB-FISH-150901-28480
Zebrafish: $dld^{tr233/tr233}$	Holley et al., 2000	ZFIN ID: ZDB-ALT-980203-1047
Oligonucleotides		
Reverse Transcriptase primer: (5′-ACG TCT CGA GTC ACC AGG GTC TCC ACA AAG GCTG-3′)	This paper	N/A
q-PCR reactions forward primer: 5′-TGG AAG AAC TGC GAA CGC TT-3′	This paper	N/A
q-PCR reactions reverse primer: 5′-CGGAGGTTTTGG ATCATGCG-3′	This paper	N/A
Software and Algorithms		
Imaris 8.1.2	Bitplane	http://www.bitplane.com/imaris/imaris; RRID:SCR_007370
Python Programming Language, version 2.7.10	Python Software Foundation	http://www.python.org/; RRID:SCR_008394
Matlab_R2016a	Mathworks	http://www.mathworks.com/products/matlab/; RRID:SCR_001622
StochKit2	Sanft et al., 2011	https://sourceforge.net/projects/stochkit/files/
ImageJ		https://imagej.nih.gov/ij/; RRID:SCR_003070
GraphPad Prism 7	GraphPad	http://www.graphpad.com/; RRID:SCR_002798
Image Processing Pipeline	This paper	Data S1
Stochastic Simulations Script	This paper	Data S2

CONTACT FOR REAGENT AND RESOURCE SHARING

Further information and requests for reagents may be directed to the Lead Contact Ertugrul Ozbudak (Ertugrul.Ozbudak@cchmc.org).

EXPERIMENTAL MODEL AND SUBJECT DETAILS

Fish stocks

All the fish experiments were performed under the ethical guidelines of Albert Einstein College of Medicine (AECOM) and Cincinnati Children's Hospital Medical Center, and animal protocols were reviewed and approved by the respective Institutional Animal Care and Use Committees (Protocol # 20150704 and Protocol # 2017-0048). Membrane-localized-GFP Tg(*b-actin:mgfp*)^{vu119} (Cooper et al., 2005) transgenic line was used as wild-type, and *dlc*$^{tw212b/tw212b}$ (Jülich et al., 2005) and *dld*$^{tr233/tr233}$ (Holley et al., 2000) were used as Notch signaling mutants. Temporal loss of function of Notch signaling was accomplished by treating embryos with 100 μM of the γ-secretase inhibitor, *N*-[N-(3,5-difluorophenacetyl)-L-alanyl]-S-phenylgly cine t-butyl ester (DAPT) while DMSO is used as a control (Ozbudak and Lewis, 2008). Fish were bred and maintained at 28.5°C on a 14–10 hr light/dark cycle.

METHOD DETAILS

smFISH and imaging

We have used the protocol developed by Advanced Cell Diagnostics (Wang et al., 2012), which allows for simultaneous detection of two differentially labeled transcripts in zebrafish (Gross-Thebing et al., 2014). 0.9 nL of membrane-localized GFP RNA was injected in one cell stage mutant embryos. Afterward, embryos were incubated at 23°C until they reach 10-14 somite stage, then embryos are fixed in 4% PFA in PBS for 1 hr at room temperature (RT), washed with 0.1% PBSTw (0.1% Tween-20 in PBS), then with gradually increasing methanol (MeOH) concentration (50% MeOH - 50 PBSTw, 100% MeOH), each wash for 5 min. Dechorionation was performed when they were in 50% MeOH - 50% PBSTw solution. Afterward, embryos were stored in 100% MeOH at −30°C overnight (O/N). The next day, embryos were air-dried for 30 min at RT and processed with Pretreat 3 (Advanced Cell Diagnostic RNAScope Pretreatment Reagents, 320842) for protease digestion for 20 min at RT. Then, they were washed with 0.01% PBSTw (0.01% Tween-20 in PBS) for 3 times each for 5 min. Probe-Dr-her7 and Dr-her1-LE2-C3 probes were mixed in 50:1 ratio, respectively, and probe mix was warmed at 40°C for 10 min in the oven, cooled down to RT, and added on embryos. Embryos were incubated in hybridization oven at 40°C for O/N. The next day all washing and signal amplification steps with RNAscope Fluorescent Multiplex Detection Reagents (Advanced Cell Diagnostics 320851) were performed according the RNAscope Protocol for Zebrafish (Gross-Thebing et al., 2014), with only few changes. Two additional washing steps were added both after the removal of probe and after the pre-amplifier hybridization step to decrease the background signal (5 times for 15 min) and each reagent was used as 1 drop. For labeling Amp4 Alt-B reagent was used. After the labeling step with Amp4, embryos were fixed in 4% PFA in PBS at 4°C for O/N. The following day, embryos were washed with 1% PBSTX (1% Triton X-100 in PBS) for 3 times each for 5 min, and permeabilized in 1.5% Triton X-100 for 1 hr at RT. Embryos were then incubated in blocking buffer (1% Triton X-100, 2%BSA, 5% Goat Serum) for 2 hr at RT. Then, embryos were incubated with Rabbit anti-GFP primary antibody (Life Technologies A6455) in blocking buffer (1:100) at 4°C for O/N. The following day, embryos were washed with 1% PBSTX 3 times for 10 min followed by a wash with blocking buffer for 10 min. Then, embryos were incubated in blocking buffer with 1:400 Hoechst trihydrochloride, trihydrate (Invitrogen 33342) and 1:200 Goat anti-Rabbit IgG Alexa 488 Secondary Antibody (A-11034, Life Technologies) at 4°C in dark for O/N. Then, embryos were washed with 0.2X SSCT (0.01% Tween-20 in 0.2X SSC) for 15 min at 4°C and fixed in 4% PFA in PBS. Embryos were mounted in 0.2X SSCT solution. ProLong Gold antifade reagent (Life Technologies P36934) was used to prepare slides. Images were captured by using a 63X (NA = 1.4) objective at the Zeiss Imager Z2. Serial sections of fluorescent images were taken at 0.240 μm intervals for up to 30 μm with AxioCamMRm camera, apotome and Axiovision software 4.8.2 Release. Images of single embryos were tiled along the x-y axis and stitched by the Axiovision software.

Quantifying RNA numbers in single cells

Imaris 8.1.2 Cell Module was used to analyze the images. First, the tissues surrounding the PSM (notochord, neural tube and lateral plate mesoderm were filtered by using the surface tool. Manual surface creation tool was selected to mark the regions covering undesired tissues and the voxels inside were set to zero. Individual PSM cells were identified by using the cell tool. Nuclei diameter threshold was set to 3 μm with smoothing width of 0.3 μm, background subtraction sphere diameter of 1.2 μm and split nuclei by seed points. Then, number of voxels filter was used to select the seed points and an intensity threshold was used for background subtraction, finally nucleus number of voxels was set to select single nucleus. Afterward, detect cell boundary from cell membrane option with 0.25 μm membrane width was selected. The cell volume filter was set between 150-450 μm^3. Finally, spots tool was used to count the total number of RNA molecules in the PSM. Estimated diameter was set to 0.5 μm and background subtraction option was selected. Quality score filter was set to separate RNA dots from background. The cell-membrane signal (GFP) got weaker in the deepest z-layers, and thus we could not successfully separate cells and collect data on the ventral-most part (Figure S1). Therefore, we have only included data from cells that we could successfully segment by image analysis. The number of clock RNAs per successfully segmented cell do not systematically depend on z axis (Figure S1F).

qRT-PCR

Full-length *her1* mRNA standard was synthesized from her1 pCS2+ clone. The plasmid was linearized by Not1 and SP6 mMessage mMachine (Life Technologies AM1340) was used for *in vitro* transcription. RNA was purified with Zymo Research Quick RNA

MiniPrep Kit (R1054). The loss of RNA during column purification was calculated by measuring the concentration of RNA by Qubit (Life Technologies) both before and after the column purification. Tails of five 13-14 somite-staged embryos were dissected and pooled in L15 media under a dissecting microscope and purified with Zymo Research Quick RNA MiniPrep Kit. Five biological replicates of this experiment were carried out. RT reactions were performed by using SuperScript IV Reverse Transcriptase (Invitrogen 18090010), RNaseOUT (Invitrogen 10777-019), dNTP (Roche 05 081 955 001) and the reverse primer: (5′-ACG TCT CGA GTC ACC AGG GTC TCC ACA AAG GCTG-3′). q-PCR reactions were performed by using the forward primer: 5′-TGG AAG AAC TGC GAA CGC TT-3′, reverse primer: 5′-CGGAGGTTTTGGATCATGCG-3′ and SYBR Select Master Mix (Life Technologies 4472908) with the Applied Biosystems StepOnePlus Real-Time PCR System. Standard RNA dilution series were prepared by 10-fold dilutions and the efficiency of primers was calculated by fitting a standard curve in the StepOne Software v2.3 (Slope: −3.32, R^2: 0.98, Y-intercept: 33.423, Efficiency: 100%). The numbers of total *her1* mRNA in tail samples were calculated by comparing their C_T values to that of the standard *her1* RNA sample. We have detected 82264 ± 10816 *her1* mRNAs per single embryo.

Calculating the efficiency of RNA detection by smFISH

We counted the total RNA numbers in the whole PSM by smFISH and compared the results to that obtained by qRT-PCR. Spots tool was used to count the total number of *her1* RNA molecules in the PSM of 18 wild-type embryos. Estimated diameter was set to 0.5 μm and background subtraction option was selected. Quality score filter was set to above 70. We subtracted the nonspecific smFISH staining as follows: Surface tool was used to count the total number of nuclei (cells) in the PSM by using the DAPI channel data. Smooth tool was selected. Surface area detail level was set to 0.3 μm. Background subtraction with local contrast was used and the diameter of largest sphere which fits into the object was set to 1.2 μm. The threshold for background subtraction was set to 100. Split touching objects was enabled with seed point diameter as 3 μm. The number of voxels filter was used to separate the nuclei from smaller objects. The total number of cells was multiplied with the background smFISH staining and the resultant values were subtracted from that obtained with the spot detection tool to obtain the total number of *her1* mRNA molecules in a single embryo: 75862 ± 24061. The ratio of the number of *her1* mRNA molecules detected by qRT-PCR versus smFISH is: 1.08 ± 0.37.

Calculating the measurement error in RNA counting by smFISH

Control smFISH experiments were carried out in wild-type embryos by using two colors of probes (Dr-her1-LE1-C1 and Dr-her1-LE2-C3) directed against the *her1* mRNA. The probe sets are designed in an alternating tiled manner complementary to the *her1* mRNA. In total, we have used 8 wild-type embryos and imaged 21-35 sections in each embryo. The number of RNAs in each cell is measured as described in the above sections. Since binding of one probe set to RNA would be independent of the other probe set, the measurement error in counting single RNA molecules can be calculated by using an equation mimicking intrinsic noise: $(1/2)<((her1C1/<her1C1>) - (her1C3/<her1C3>))^2>$. The measurement error in RNA counting is: 0.03.

Calculating the measurement error in cell segmentation

One of the sources of measurement error would be incorrectly segmenting cells from each other, i.e., failing to assign image voxels unambiguously to a single cell. To estimate the cell segmentation error, we in total selected 38 image sections from 7 different wild-type embryos. We manually segmented 255 cells from this dataset by ImageJ. For each cell, we compared the manually segmented areas in ImageJ with the automatically segmented areas in Imaris. The measurement error of misassigning mRNAs to single cells is calculated as follows:

Let x_i be an independent and identically distributed (iid) random variable denoting the actual mRNA count in a cell with mean $\langle x \rangle$ and coefficient of variation squared CV^2. Assuming each cell has a normalized area of 1, the measurement is given by

$$x_i(1-a_i)+c_i x_j, \quad (1)$$

where a_i is an iid random variable denoting the fractional area ignored, x_j is the mRNA count in the neighboring cell, and c_i is an iid random variable denoting the fractional area gained of the neighboring cell. The mean and variance of a_i is given by $\langle a \rangle$ and σ_a^2, respectively. Similarly, the mean and variance of c_i is defined as $\langle c \rangle$ and σ_c^2. Then, the error in the measurement would be

$$x_i(1-a_i)+c_i x_j - x_i = c_i x_j - a_i x_i. \quad (2)$$

Taking the variance of (2) and dividing by $\langle x \rangle^2$ quantifies the error in terms of the coefficient of variation squared:

$$CV^2\left(\langle a \rangle^2+\langle c \rangle^2\right)+\left((\langle a \rangle-\langle c \rangle)^2+\sigma_a^2+\sigma_c^2\right). \quad (3)$$

We calculated the segmentation error at different total *her* mRNA levels (Figure 2G). The measurement error values in cell segmentation ranged between 0.02 and 0.03.

Grouping cells in space based on oscillation phases

The position of each cell and the number of *her1* and *her7* RNA molecules in each cell were measured in each embryo. Each embryo was divided into left and right halves by visual inspection (Figure 1F). Within each half PSM tissue, cells were grouped (sliced) based on their oscillation phases, which vary smoothly along the axis (Giudicelli et al., 2007). The slice width was set to 8 μm, which

corresponds to the diameter of cells in the PSM. The angles of slices were fixed to the angle of expression stripes of the segmentation clock in the PSM. The angle of expression stripes of the segmentation clock gene changes incrementally along the posterioanterior direction in the PSM. We first measured the stripe angles along the axis in all wild-type and DAPT-/DMSO-treated samples. We then fitted an equation to the data (Figure S1G). We obtained the following equations: wild type angle = $0.039 * \text{Distance} + 44.23$, DMSO angle = 0.057 Distance + 43.45, and DAPT angle = 0.123 Distance + 21.43, where distance (μm) is measured from the tail end of embryos. Later on, we used these equations to incrementally change the angles of slices along the PSM in all embryos in each background. For *deltaC* and *deltaD* mutant embryos, we have used the angle function of the wild-type embryos. We assigned each cell into a slice when the center of the cell is located within a spatial slice.

Calculating the measurement error in phase grouping of cells

The calculation of expression noise depends on assignment of cells to correct oscillation phase bins. Part of the extrinsic noise could be due to slight errors in the assessment of cell positions rather than coregulation. To address this issue, the anterior stripe expression angles for wild-type embryos are calculated manually by two different experimentalists. We calculated two total expression noise values at different total *her* mRNA levels by using these two different angle sets. The measurement error is set to be the difference between total expression noise values obtained by using two independent measurements of expression angles. The measurement error in phase grouping of cells ranged between 0.01 and 0.05.

Calculating the total measurement error

We added the measurement errors due to RNA counting, cell segmentation and phase grouping of cells to obtain the total measurement error in our experimental and analysis pipeline at five total *her* mRNA levels (Figure 2G). The total measurement errors were between 0.066 and 0.098. We plotted the total measurement error in Figure 2G as a baseline to biological variability (expression noise) that can be unambiguously assayed.

QUANTIFICATION AND STATISTICAL ANALYSIS

Heatmaps

For visual aid, we created heatmaps for each embryo by grouping the cells into two groups: cells with low or high mRNA numbers (Figure 1F). The threshold for this grouping was chosen as follows. First, the minimum (N) and maximum (M) mRNA levels per cell were determined in the embryo. Then the threshold was chosen as N+0.3*(M-N) by visual inspection. The cells below this threshold were assigned as low expression cells, and the rest were assigned as high expression cells. The heatmaps were created by plotting high expression cells with bold colors and low expression cells with light colors. Slice boundaries were shown in each heatmap (as in Figure 1F).

The details of the data analysis and computational modeling can be found in Supplemental Experimental Procedures.

Cell Reports, Volume *23*

Supplemental Information

Noise in the Vertebrate Segmentation Clock Is Boosted by Time Delays but Tamed by Notch Signaling

Sevdenur Keskin, Gnanapackiam S. Devakanmalai, Soo Bin Kwon, Ha T. Vu, Qiyuan Hong, Yin Yeng Lee, Mohammad Soltani, Abhyudai Singh, Ahmet Ay, and Ertuğrul M. Özbudak

SUPPLEMENTARY FILES

SUPPLEMENTAL METHODS

Subtracting nonspecific staining

In each genetic background, we quantified the nonspecific smFISH staining in the segmented somites, where *her1* and *her7* genes are not expressed. Mean and variance of nonspecific staining were given in Table S1.

Mean nonspecific staining levels were subtracted from each cell's mean *her1* and *her7* mRNA levels. Afterwards, cells with negative *her1* or *her7* mRNA levels were removed from the data. Slices with fewer than three cells after background subtraction were also removed from the data.

Spatial gene expression dynamics

Raw data was processed differently for this analysis. If a slice had negative mean of either *her1* or *her7* mRNA after background subtraction, the negative mean expression level was set to zero and the slice was not eliminated. For left and right halves of each embryo, *her1* and *her7* mean mRNA levels (y-axis) at each spatial location (slice) were plotted (x-axis). Spatial location 0 corresponded to the posterior (tail) end of embryos (Figure 1G).

Amplitude measurement

Slices with negative mean mRNA level were not eliminated but rather mRNA levels were set to zero. Amplitudes of *her1* and *her7* gene expression levels were calculated as follows:

Mean mRNA levels of each slice from all embryos were used to calculate amplitude for each genetic background. In this approach, we first combined the data from individual embryos in each genetic background as follows. First, we aligned the posterior ends of all embryos. Then, we combined the data from the *i*th slices from both left and right halves of all embryos. This iterative process was performed until one of the embryos ran out of slices. By this way, we collected 36, 28, 36, 18 and 24 data points for each cell position from 18 wild-type, 14 *deltaC*$^{-/-}$ mutant, 18 *deltaD*$^{-/-}$ mutant, 9 DAPT-treated, and 12 DMSO-treated embryos, respectively. To reduce the effect of outliers: a) We grouped data from every five consecutive spatial locations. Within each spatial group, mRNA levels of all slices were ranked from largest to smallest. b) Mean of the top 10% of the group was assigned as the peak and mean of the bottom 10% was assigned as the trough. The difference between the peak and trough in each group was defined as the amplitude at that location. We tested the effect of binning on amplitude values by varying the number of spatial groupings from 4 to 8 and top/bottom groupings from 5% to 10%. These modifications did not change the amplitude values more than 16%. The spatial profile of total *her* mRNA amplitude is plotted in Figure S2A. We then averaged the amplitude values across the space and plotted the average amplitudes in Figure 2B.

Noise formulas

The intrinsic and extrinsic noise levels for each slice were computed using the equations below. Total noise was calculated as the sum of intrinsic and extrinsic noise.

$$intrinsic\ noise = \frac{1}{2} < \left(\frac{her1}{< her1 >} - \frac{her7}{< her7 >}\right)^2 >$$

$$extrinsic\ noise = \frac{< her1\ \cdot her7 > - < her1 >< her7 >}{< her1 >< her7 >}$$

$$\mathrm{CV}^2 = total\ noise = \ intrinsic\ noise + \ extrinsic\ noise$$

For each genetic background, the three noise levels were plotted at different mean *her* (*her1*+*her7*) mRNA levels. We have reported how gene expression noise varies with respect to mean *her* expression levels among phase grouped cells (single-cell diameter spatial slices) in wild-type embryos in Figure 2F. Later on, we have grouped the noise data of individual slices (Figure 2F) into 5 bins according to their mean *her* RNA numbers (Figure 2G).

The contribution of differences in cell volumes on expression noise

We calculated the concentration of mRNAs in each cell by dividing the number of mRNAs in each cell by the cell volume. Later, we normalized the resulting values to report the number of mRNAs per 4 pl volume. After this normalization, the noise plots reported in Figure 2G and Figure S2B had comparable ranges on the x-axis.

Spatial variation in noise

The CV^2 data from all slices in all embryos in each genetic background was combined as described in the "Amplitude measurement" section. CV^2 values of the *i*th slices from both left and right halves of all embryos were combined. The mean and standard error of CV^2 values were plotted at different spatial positions (Figure 4B).

To control the dependency of spatial CV^2 values on expression levels, we carried out two further analyses:

1) The data were grouped into three categories depending on its expression level: low, medium and high. Slices were first ranked from the highest to the lowest gene expression levels. Then, they were divided into three equally sized groups: bottom 33.33% (low expression), middle 33.33% and top 33.33% (high expression). For each spatial location, three different CV^2 values (at low, medium and high mRNA levels) and their standard errors were calculated by using the sum of *her1* and *her7* mRNA levels at each genetic background (wild-type, *deltaC*$^{-/-}$ and *deltaD*$^{-/-}$; Figure S4A).

2) The embryos' slices are placed into 8 groups based on their mean total *her* expression levels. Slices with expression levels from 0 to 15 formed the first group, and slices from 105 to 120 formed the last group. The slices above 120 are considered to be outliers, and eliminated from the analysis. Afterwards, for each expression group, the data coming from slices located in the posterior 33% and anterior 33% of the PSM are selected. Then, the mean plus two standard error of expression level, and mean plus two standard error of CV^2 in posterior and anterior slices are calculated. The figure summarizing this analysis is provided in Figure 4C, D.

Statistical analysis

One-way ANOVA was conducted to determine the statistical significance of the differences between amplitude and noise in different genetic backgrounds and/or spatial locations. Normality was assessed by visual inspection of histograms and normal Q-Q plots. Homogeneity of variances was assessed by comparing standard deviations of different groups.

Computational Model

We built a computational model to simulate synchronized oscillation in two neighboring cells. In this model, a cell (Cell a) produces a protein Z^a from a constitutive gene as shown in Figure S5A. This protein activates the production of two other proteins X^a and Y^a. The protein X^a inhibits the production of the protein Z^a after a time-delay τ_x, while the protein Y^a activates the production of Z in the neighboring cell (Cell b) after another delay τ_y. The same analogy is true for the neighboring cell. Overall, the production rate of protein Z^a at time t is given by

$$k_z - k_x x^a(t-\tau_x) + k_y y^b(t-\tau_y), \quad (1)$$

where x^a (y^b) denotes the count level of X^a (Y^b) at time $t-\tau_x$ ($t-\tau_y$). Further k_z, k_x, and k_y are basal production rate, negative feedback strength, and positive feedback strength, respectively. Finally, protein Z decays with rate γ.

The biological representation of the aforementioned time-delays is as follows: The time-delays in Figure S5A are implemented by considering that the proteins X and Y will be activated after some time. Hence, we consider that the protein Z^a produces two intermediate molecules X_1^a and Y_1^a. The intermediate molecule X_1^a (Y_1^a) is converted into its active form X_n^a (Y_m^a) after undergoing a series of n (m) first-order conversion reactions with the rate n/τ_x (m/τ_y). Assuming that the timing of first-order reactions are similar and exponentially distributed, the conversion process creates a gamma-distributed delay in the activation of the proteins with mean time-delays τ_x and τ_y. In addition, noise in time-delays quantified by Coefficient of Variation (CV) squared is

$$CV_{\tau_x}^2 = \frac{\langle \tau_x^2 \rangle - \langle \tau_x \rangle^2}{\langle \tau_x \rangle^2} = \frac{1}{n}, \quad CV_{\tau_y}^2 = \frac{\langle \tau_y^2 \rangle - \langle \tau_y \rangle^2}{\langle \tau_y \rangle^2} = \frac{1}{m}, \quad (2)$$

where $\langle . \rangle$ denotes the expected value. The activated protein X^a inhibits the expression of the protein Z^a and the activated protein Y^a enhances the production of protein Z^b in the neighboring cell. It means that now the production rate of protein Z^a is

$$k_z - k_x x_n^a(t) + k_y y_m^b(t), \tag{3}$$

where $x_n^a(t)$ and $y_m^b(t)$ denote the count levels of X_n^a and Y_m^b at time t, respectively. Finally, the protein Z decays with rate γ. The overall model in Cell a consists of the following chemical reactions:

$$\begin{gathered}
\emptyset \xrightarrow{k_z - k_x x_n^a(t) + k_y y_m^b(t)} Z^a, \qquad Z^a \xrightarrow{\gamma} \emptyset, \\
Z^a \xrightarrow{n/\tau_x} Z^a + X_1^a, \\
X_i^a \xrightarrow{n/\tau_x} X_{i+1}^a, \quad i = \{1, \dots, n-1\}, \\
X_n^a \xrightarrow{n/\tau_x} \emptyset, \\
Z^a \xrightarrow{m/\tau_y} Z^a + Y_1^a, \\
Y_i^a \xrightarrow{m/\tau_y} Y_{i+1}^a, \quad i = \{1, \dots, m-1\}, \\
Y_m^a \xrightarrow{m/\tau_y} \emptyset.
\end{gathered} \tag{4}$$

The model in Cell b is identical to that of Cell a.

We run Monte Carlo simulations to test our model numerically. Our code is written based on SSA algorithm (Gillespie, 1976) and we use the software StochKit2 to simulate the model (Sanft et al., 2011). In our code, we checked a wide range of time-delays. We start with time-delays $\tau_x = 12.15\ mins$ and $\tau_y = 24.3\ mins$ and we increased the time-delays up to $\tau_x = 54\ mins$ and $\tau_y = 108\ mins.$, i.e. ≈ 4.5 fold change in time-delays as measured previously (Ay et al., 2014). We considered that $m = n = 10$, which means noise in time-delays is $CV_{\tau_x}^2 = CV_{\tau_y}^2 = 0.1$. Further note that, to maintain the oscillations of two cells in synchrony, we increase the time-delays with the same ratio. The protein degradation rate is $\gamma = 0.2/\ min$, which means the protein halflife is 3.46 $mins$ as measured previously (Ay et al., 2013). Finally, we select the

rates as $k_x = 0.6$ and $k_y = 0.2$, and by changing delay, we also change k_z to keep the mean of protein Z at 55 ± 1.

Figure S5B illustrates that this model can replicate the synchronized oscillations by interplay between intracellular negative feedback and intercellular positive feedback. Our goal is to calculate the noise in a population of synchronized cells, however due to model size, increasing the number of cells results in increasing the complexity of model. Hence, instead of increasing the number of cells in our code, we used the peak values of oscillations in a cell as a proxy of a population of synchronized cells. To do so, after simulating the model using StochKit, we used a MATLAB code to process the data. In this code, we first read the data from the files that StochKit made. In the next step, we smoothened the data to remove the fluctuations contributed from random production and degradation events. The smoothened data was used to find the peak times. The difference between these times gave the period of oscillations. Afterwards, we extracted the peak levels from the unfiltered data associated with these peak times. The expression variability at the peak levels was defined as expression noise. The StochKit and MATLAB codes used in computational modeling are provided in supplementary information.

SUPPLEMENTARY FIGURES and FIGURE LEGENDS:

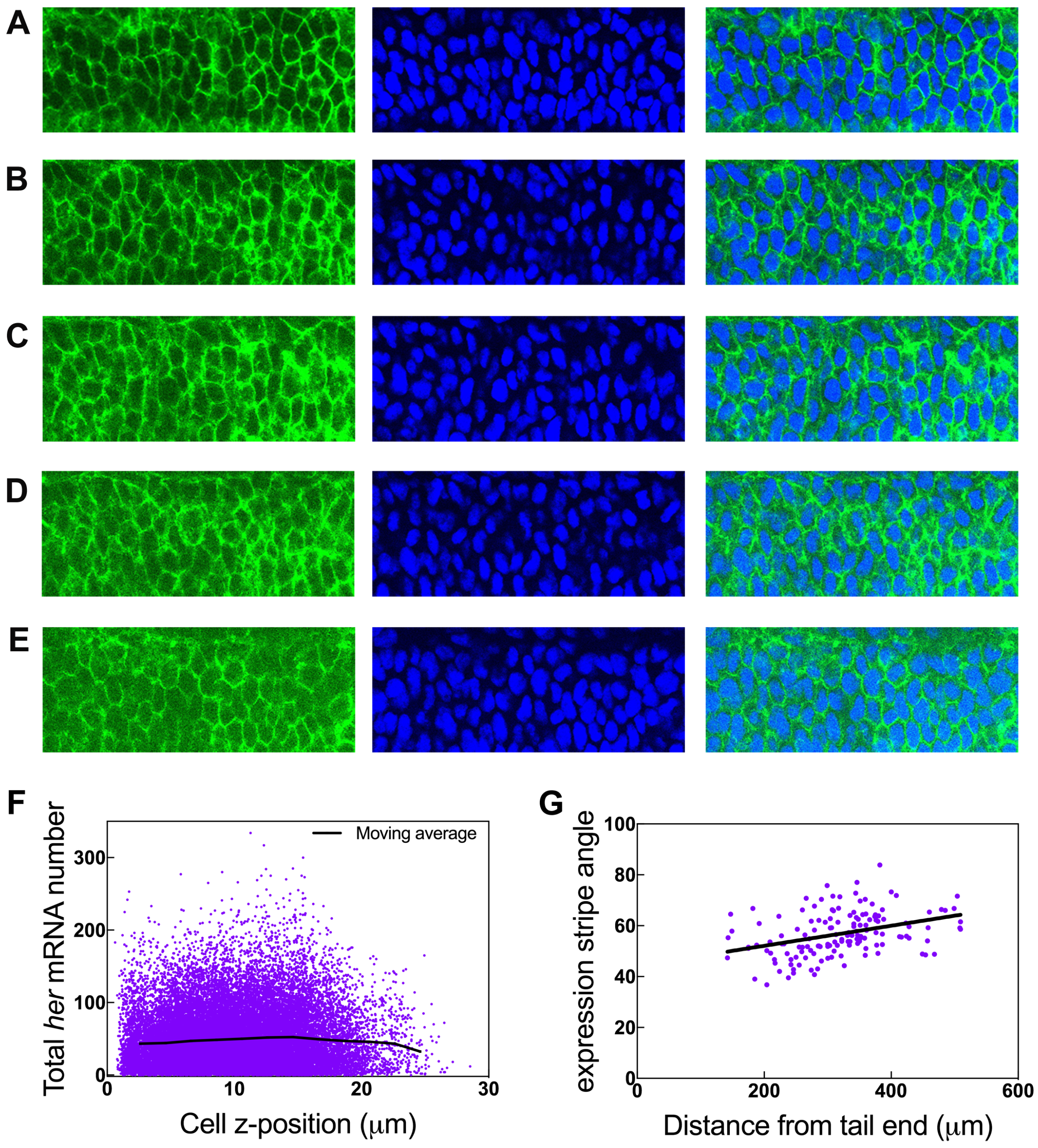

Figure S1. Representative z-sections of an smFISH image and the gradual change in the angle of clock expression stripes, related to Figure 1

(A-E) Different z-sections of an smFISH image show the cell membrane (green), nuclear (DAPI) and merge images in a wild-type embryo, respectively. Images belong to sections 25, 35, 45, 55 and 65 in z-axis, respectively. (F) Total *her* (*her1+her7*) RNA in each cell and the position of each cell are plotted for all cells from all wild-type embryos. Tissue sections further away from the microscopy objective have higher z-values. Successful detection of RNAs does not systematically depend on z-position in microscopy images. The data is first grouped according to z-position with 2μm bin size. Then, a moving average is calculated by using 3 bins. The centered moving average is plotted in the graph. (G) The angle between the clock expression stripes and the AP axis increases incrementally in the posterioanterior direction (away from the tail end of the embryos). The angle data from wild-type embryos is shown here. A linear function ($y = 0.039 * x + 44.23$) fits to the data ($R^2 = 0.14$).

A

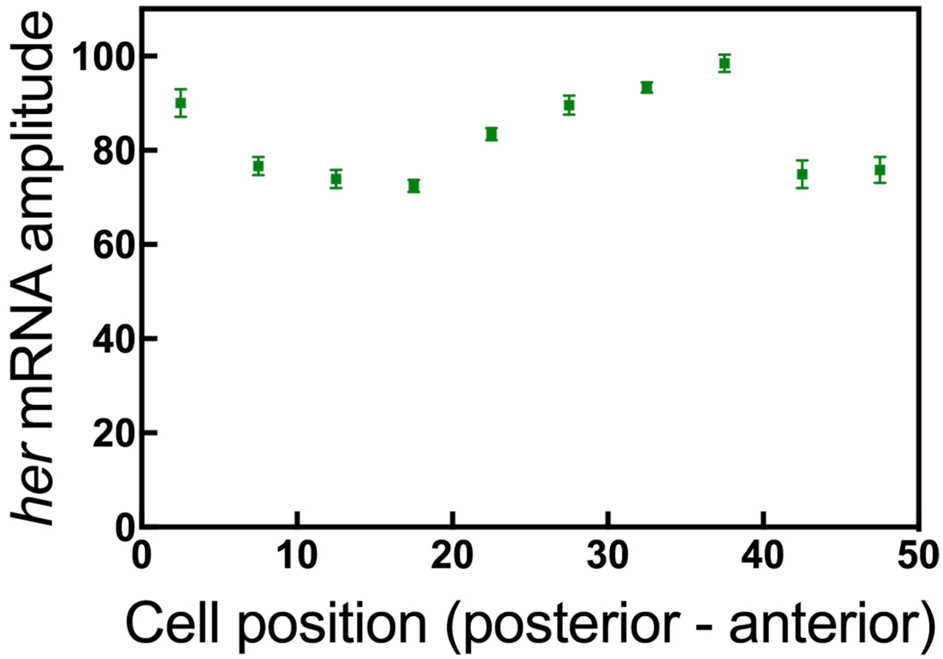

her mRNA amplitude
0
20
40
60
80
100
0
10
20
30
40
50
Cell position (posterior - anterior)

B

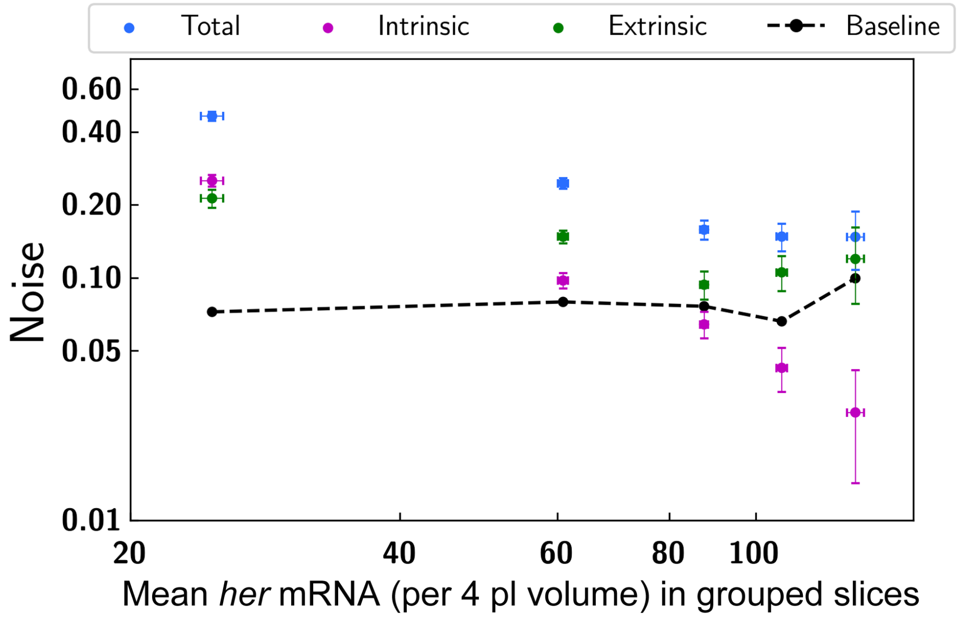

Total
Intrinsic
Extrinsic
Baseline
Noise
0.60
0.40
0.20
0.10
0.05
0.01
20
40
60
80
100
Mean her mRNA (per 4 pl volume) in grouped slices

Figure S2. Spatial profile of total *her* mRNA amplitudes and the effect of cell volume on expression noise, related to Figure 2

(A) Oscillation amplitudes of total *her* RNA (*her1*+*her7*) do not change drastically along the PSM. Error bars are two standard errors. (B) Differences in cell volumes have a mild contribution in clock gene expression noise. y-axis is noise; x-axis is mean levels of total *her* (*her1*+*her7*) RNA (per 4 pl volume) in grouped slices.

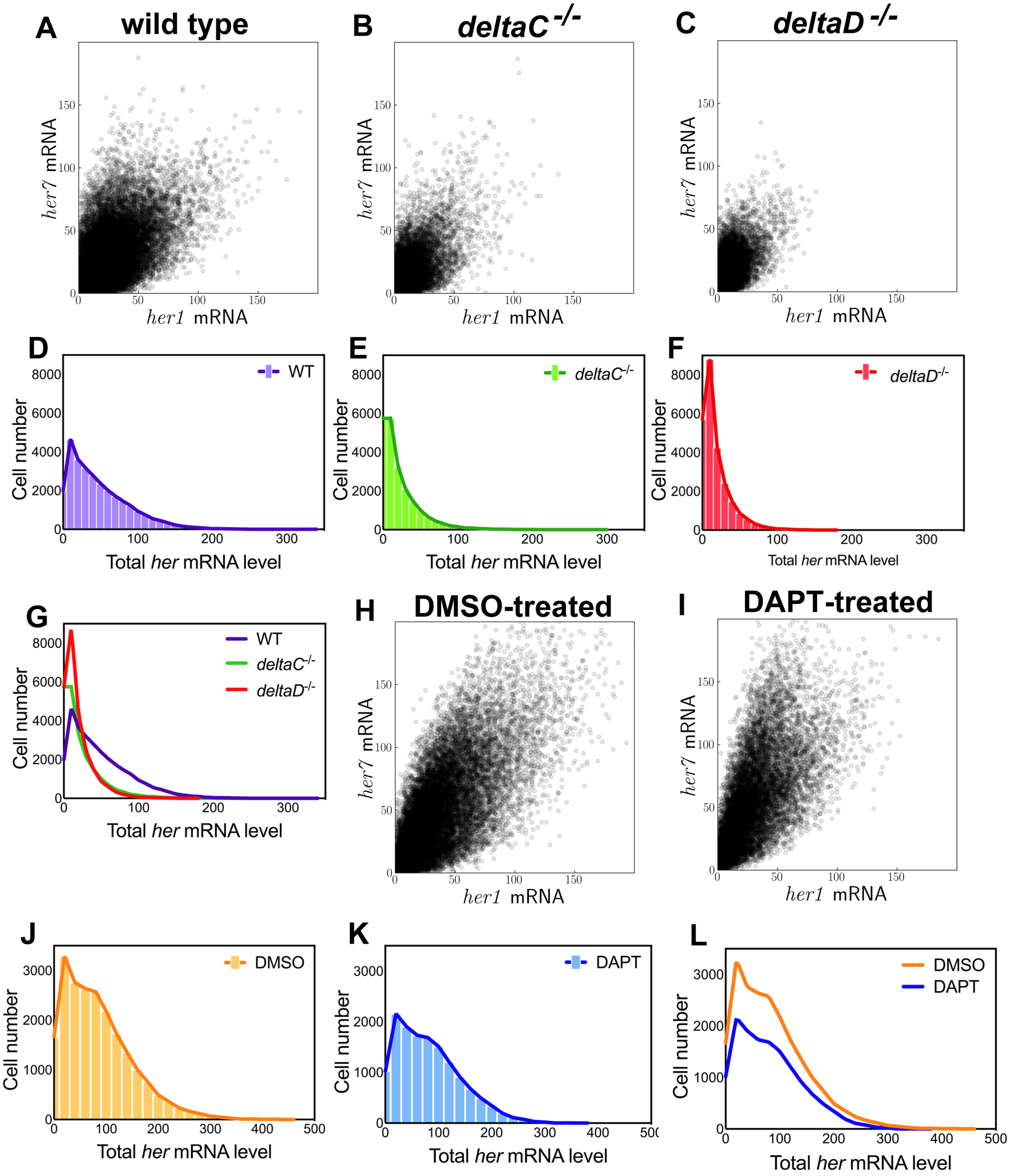
A
wild type
B
deltaC -/-
C
deltaD -/-
her7 mRNA
her1 mRNA
D
E
F
Cell number
Total her mRNA level
WT
deltaC-/-
deltaD-/-
G
H
DMSO-treated
I
DAPT-treated
J
K
L
DMSO
DAPT

Figure S3. *her1* versus *her7* RNA numbers and the distribution of RNA numbers in each cell in different genetic and drug-treated backgrounds, related to Figures 2 and 3

(A-C) Scatter plot of *her1* versus *her7* RNA levels in each cell in all embryos in wild-type (A), *deltaC*$^{-/-}$ (B) and *deltaD*$^{-/-}$ (C) backgrounds, respectively. (D-G) The distribution of cell numbers with a given total *her* (*her1*+*her7*) RNA number in wild-type (D), *deltaC*$^{-/-}$ (E), *deltaD*$^{-/-}$ (F), and overlaid (G) backgrounds, respectively. The data came from 28904 cells from 18 wild-type, 21560 cells from 14 *deltaC*$^{-/-}$ mutant and 24997 cells from 18 *deltaD*$^{-/-}$ mutant embryos. (H, I) Scatter plot of *her1* versus *her7* RNA levels in each cell in all DMSO-treated and DAPT-treated embryos. (J-L) The distribution of cell numbers with a given total *her* (*her1*+*her7*) RNA number in DMSO-treated embryos (J), in DAPT-treated embryos (K) and overlaid (G), respectively. The data came from 14091 cells from 9 DAPT-treated and 21497 cells from 12 DMSO-treated embryos.

A

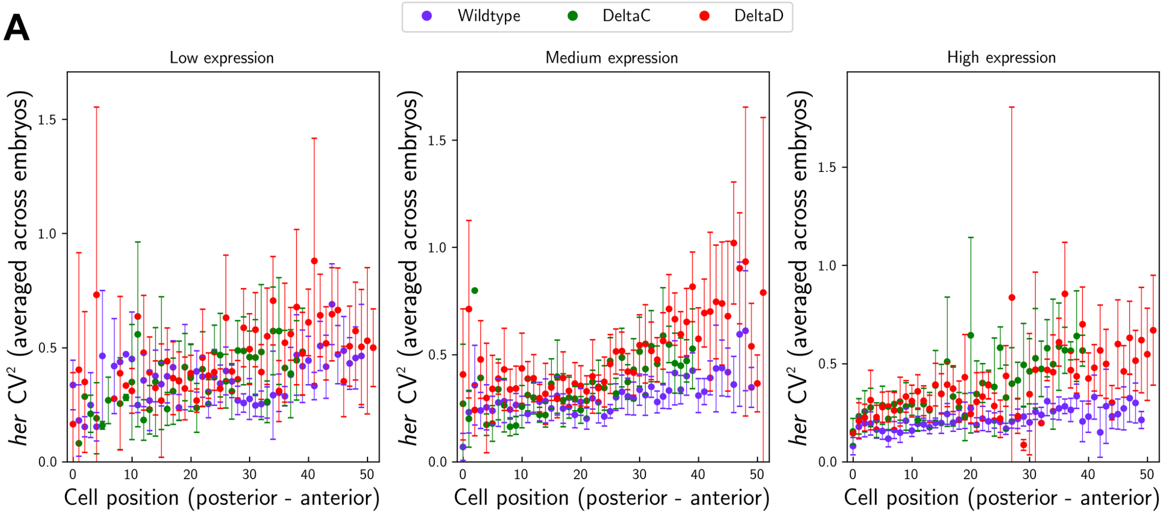
Wildtype
DeltaC
DeltaD
Low expression
Medium expression
High expression
her CV² (averaged across embryos)
Cell position (posterior - anterior)

B

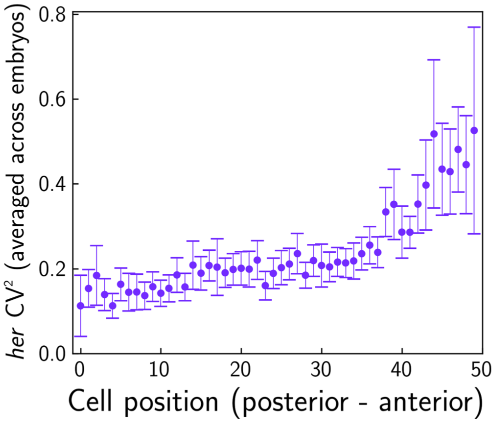
her CV² (averaged across embryos)
Cell position (posterior - anterior)

Figure S4. Expression noise displays an increasing profile along the unsegmented axis, related to Figure 4

(A) Expression noise is analyzed in the PSM for total *her* (*her1* + *her7*) mRNAs in wild-type (purple), *deltaC*$^{-/-}$ (green) and *deltaD*$^{-/-}$ (red) mutant embryos. Data is grouped based on average expression levels (left column for low, middle column for medium and right column for high mRNA). Noise is averaged across all embryos at a given spatial position in each genetic background. X-axis is spatial positions; error bars are two standard errors. (B) The clock gene expression noise displays a spatially increasing profile along the posterioanterior direction in the PSM even when the differences in cell volumes are taken into account.

A

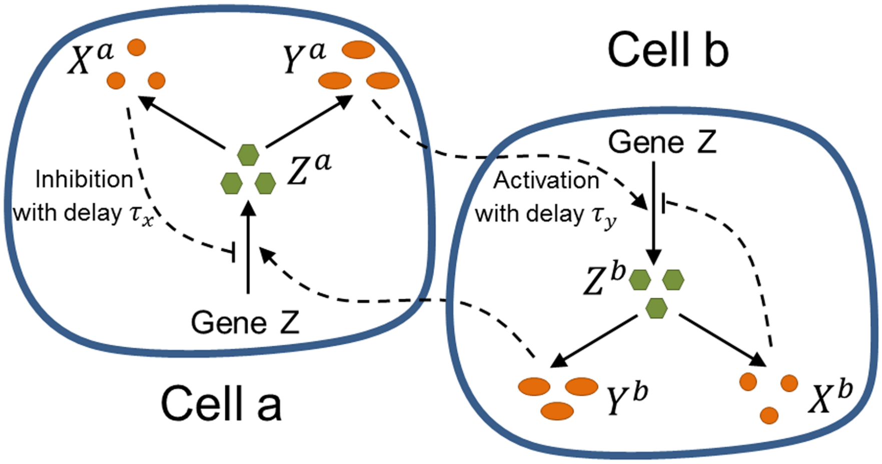

X^a
Y^a
Z^a
Inhibition
with delay τ_x
Gene Z
Cell a
Cell b
Gene Z
Activation
with delay τ_y
Z^b
Y^b
X^b

B

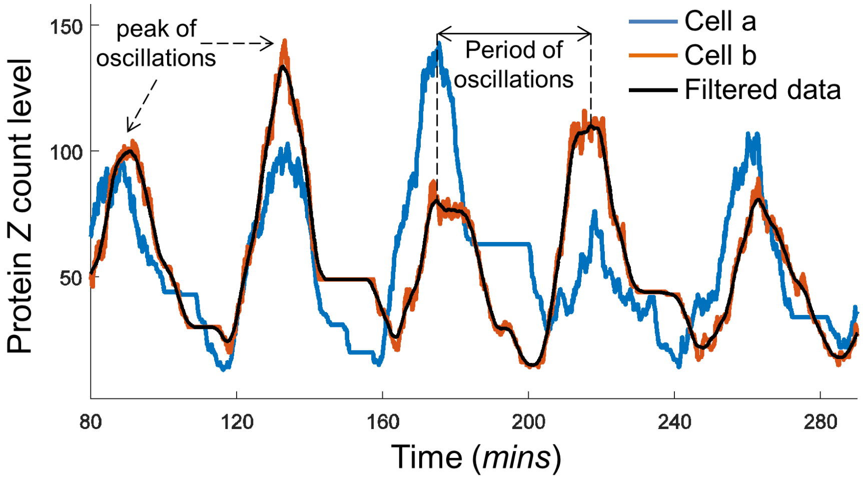

peak of
oscillations
Period of
oscillations
Cell a
Cell b
Filtered data
Protein Z count level
150
100
50
80
120
160
200
240
280
Time (mins)

Figure S5. The stochastic model simulates two coupled cells displaying synchronized oscillations, related to Figure 4

(A) A stochastically expressed protein Z activates production of two other proteins X and Y. The protein X inhibits the production of the protein Z in its own cell after a time delay. The protein Yactivates the production of the protein Z in the neighboring cell after another time delay. (B) Time trend of two cells oscillating in synchrony is used to calculate the noise and the period of oscillations. The period of oscillations is calculated by looking at the time intervals in which protein level in 'Cell b' reaches to its peak. The noise in protein is calculated for the points in which the protein level in 'Cell b' is at the peak of oscillations.

SUPPLEMENTARY TABLE:

**Table S1.**	***her1***		***her7***	
	Mean	Variance	Mean	Variance
**wildtype**	3.1	15.3	1.3	3.6
deltaC	3.9	12.7	1.3	2.6
deltaD	2.8	8.8	1.3	2.4
**DAPT**	4.4	3.0	1.0	1.3
**DMSO**	4.7	3.3	2.7	4.1

Table S1. Mean and variance of nonspecific smFISH staining, Related to Figure 1
